# Geministatins: new depside antibiotics from the fungus *Austroacremonium gemini*

**DOI:** 10.1038/s41429-024-00755-x

**Published:** 2024-06-26

**Authors:** Andrew Crombie, John A. Kalaitzis, Rachel Chen, Daniel Vuong, Alastair E. Lacey, Ernest Lacey, Roger G. Shivas, Yu Pei Tan, Nicolau Sbaraini, Yit-Heng Chooi, Andrew M. Piggott

**Affiliations:** 1 Microbial Screening Technologies, Smithfield, NSW 2164 Australia; 2 School of Natural Sciences, Macquarie University, Sydney, NSW 2109 Australia; 3 Department of Agriculture and Fisheries, Plant Pathology Herbarium, Dutton Park, QLD 4102 Australia; 4 Centre for Crop Health, University of Southern Queensland, Toowoomba, QLD 4350 Australia; 5 School of Molecular Sciences, The University of Western Australia, Perth, WA 6009 Australia

**Keywords:** Structure elucidation, Soil microbiology

## Abstract

Two new depside antibiotics, geministatins A (**1**) and B (**2**), were isolated from the fungus *Austroacremonium gemini* MST-FP2131 (*Sordariomycetes*, *Ascomycota*), which was recovered from rotting wood in the wet tropics of northern Australia. The structures of the geministatins were elucidated by detailed spectroscopic analysis, chemical degradation and comparison with literature values. Chemical degradation of **1** and **2** yielded three new analogues, geministatins C–E (**3**–**5**), as well as a previously reported compound dehydromerulinic acid A (**6**). Compounds **1**, **2** and **6** exhibited antibacterial activity against the Gram-positive bacteria *Bacillus subtilis* (MIC 0.2–1.6 µg mL^−1^) and *Staphylococcus aureus* (MIC 0.78–6.3 µg mL^−1^), including methicillin-resistant *S. aureus* (MRSA), while **4** exhibited antifungal activity against the yeast *Saccharomyces cerevisiae* (MIC 13 µg mL^−1^).

## Introduction

The microbial biosphere has always been a rich source of new antibiotics. The discovery of penicillin from *Penicillium italicum* triggered a cascade of new antibiotic discovery that spanned over 70 years [1]. Thousands of new antibiotics have been reported with hundreds reaching commercial development for human and animal health use globally [2]. Since originally sparking this therapeutic revolution in the 20th century, fungi have been relatively minor contributors compared to bacteria, with only three main classes (penicillins, cephalosporins and pleuromutilin) emerging to prominence [2–4]. Whether isolated from fungi or bacteria, almost all novel antibiotics owe their discovery to the intertwined philosophy that chemical novelty is a function of taxonomic uniqueness. For example, teixobactin [5] is a first-in-class depsipeptide antibiotic targeting the lipid II component of bacterial cell walls [6], which was isolated with the aid of iChip technology from a previously unculturable Gram-negative bacterium, *Eleftheria terrae*. Malacidins A and B [7] are macrocyclic lipopeptide antibiotics that target lipid II in a calcium-dependent manner, which were isolated by culture-independent heterologous expression of biosynthetic genes recovered from environmental DNA. Clearly, new taxa continue to present innovative antibiotic candidates, offering an unbroken pipeline from the microbiome.

During the course of our research into the taxonomic novelty of Australian fungi associated with wood rot in natural habitats, we discovered a new fungal genus and species, *Austroacremonium gemini* (MST-FP2131), belonging to the *Sordariomycetes* [8]. *Austroacremonium* is monotypic, with the type species being *A. gemini*. The crude MeOH extract of a small-scale culture of *A. gemini* grown on malt extract agar (MEA) showed noteworthy antibiotic activity against *Bacillus subtilis*, prompting a more comprehensive investigation into the secondary metabolites produced by this fungus. Large-scale cultivation of *A. gemini* followed by chromatographic separation and structure elucidation led to the identification of two new depside antibiotics, which we named geministatins A (**1**) and B (**2**). In this paper, we describe the cultivation process we used for *A. gemini*, together with isolation, structural elucidation, chemical degradation and biological screening of the geministatins.

**Fig. 1:**
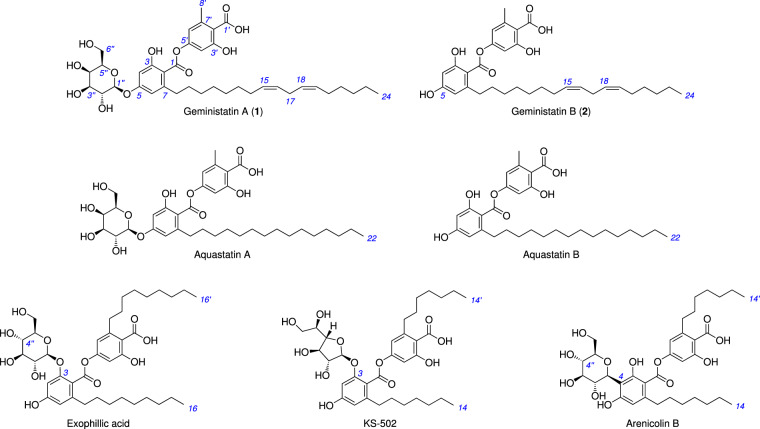
Structures of geministatins A (**1**) and B (**2**), as well as closely related known metabolites

Depsides are a family of polyketides consisting of two or more ester-linked hydroxybenzoic acid monomers. The geministatins are structurally related to several previously reported fungal depsides, including the aquastatins [9] from *Fusarium aquaeductuum*, exophillic acid [10] from *Exophiala* sp., KS-502 [11] from *Sporothrix* sp. and the arenicolins [12] from *Penicillium arenicola* (Fig. 1). These metabolites exhibited a range of noteworthy biological activities, with aquastatin A inhibiting mammalian ATPases as well as enoyl-acyl carrier protein reductase [13], exophillic acid inhibiting HIV-1 integrase and preventing cellular entry of hepatitis B and D viruses [14], KS-502 inhibiting calcium and calmodulin-dependent cyclic-nucleotide phosphodiesterase [15] and arenicolin A exhibiting cytotoxicity against mammalian cancer cell lines [12].

## Experimental procedure

### Instrumentation

Analytical HPLC was performed on a gradient Agilent 1260 Infinity quaternary HPLC system. The column was an Agilent Zorbax SB-C_18_ (2.1 × 50 mm; 1.8 μm) eluted with a 0.6 mL min^−1^ gradient of 10–100% MeCN/H_2_O (0.01% TFA) over 11 min. Preparative HPLC was performed on a gradient Shimadzu HPLC system comprising two LC-20AP preparative liquid pumps with static mixer, SPD-M10AVP diode array detector and CBM-20A system controller with standard Rheodyne injection port. The columns used in the purification of the metabolites were either an Agilent Zorbax SB-C_18_ column (50 × 150 mm; 5 µm) eluted isocratically at 60 mL min^−1^ or an Agilent Zorbax SB-C_18_ column (21.2 × 250 mm; 5 µm) eluted isocratically at 20 mL min^−1^, eluted isocratically with MeCN/H_2_O with either 0.01% TFA or 0.1% TFA modifier, as described for each separation. LCMS was performed on an Agilent 1260 Infinity series HPLC equipped with an Agilent 6120 Infinity series single quadrupole mass detector in both positive and negative ion modes. High resolution electrospray ionisation mass spectra (HRESIMS) were obtained using an Agilent 1260 Infinity series HPLC equipped with an Agilent 6230 LC/TOF. Ozone was produced using a GL-3189A ozone generator with an ozone output of 10 mg min^−1^. Optical rotations were recorded on a Jasco P-2000 polarimeter using a 10 mm quartz cell. UV-vis spectra were recorded on a Jasco V-730 UV-Visible spectrophotometer. IR spectra were recorded using a Jasco FT/IR-4700 with an ATR ProONE (ZnSe crystal) attachment. NMR data were recorded in DMSO-*d*_6_ on a Bruker Avance II DRX-600K spectrometer. All NMR spectra were recorded at 25 °C, processed using Bruker Topspin 4 software and referenced to residual solvent signals (DMSO-*d*_6_: *δ*_H_ 2.49/*δ*_C_ 39.5).

### Strain taxonomy and identification

*A. gemini* was isolated from a piece of rotting wood collected from woodland in tropical northern Australia in October 1975. Multi-locus phylogenetic analysis using marker sequences extracted from the assembled genome identified the fungal isolate as the founding member of a new genus *Austroacremonium* belonging to the taxonomic class *Sordariomycetes* with the type species named as *A. gemini* [8]. *A. gemini* is only known from the ex-type isolate MST-FP2131.

### Media optimisation

A preserved culture of *A. gemini* (MST-FP2131) was recovered onto MEA plates, which were incubated for 7 d at 24 °C. Squares of agar from the MEA recovery plates were used as inoculum for solid media optimisation on various agar and grains. After incubating for 7 d at 24 °C, two discs (each 2 cm diameter) were cut from the agars and were extracted with MeOH (2 mL), while subsamples of the grains (5 g) were extracted with MeOH (10 mL). The MeOH extracts were analysed by analytical HPLC to assess their secondary metabolite production and relative yields.

### Preparative cultivation & isolation

*A. gemini* (MST-FP2131) was grown on sterilised (121 °C for 40 min) jasmine rice in 85 × 250-mL Erlenmeyer flasks each containing 50 g of rice. Agar squares from a 7-d culture on Petri plates were used as inoculum for the flasks. The cultures were incubated at 24 °C for 14 d and the grains were pooled and extracted with acetone (2 × 4 L). The combined extracts were reduced *in vacuo* to produce an aqueous slurry (1 L). The slurry was partitioned against EtOAc (2 × 4 L) and the combined EtOAc layer was dried *in vacuo* to give a crude extract (42 g). The extract was redissolved in 90% MeOH/H_2_O (500 mL) and defatted with hexane (2 × 500 mL) to provide an enriched extract (38 g). The enriched extract was adsorbed onto silica gel (85 g) and dry-loaded onto a silica gel column (100 g; 300 × 50 mm). The column was washed once with hexane, then eluted with 50% CHCl_3_/hexane, 75% CHCl_3_/hexane and 100% CHCl_3_, followed by a stepwise gradient of 1, 2, 4, 8, 16, 32 and 100% MeOH/CHCl_3_ (500 mL each step), to yield 11 fractions (Fr 1–11). A subsample of Fr 9 (2.3 g) was purified by isocratic preparative HPLC (Zorbax C_18_, isocratic 70% MeCN/H_2_O containing 0.1% TFA, 60 mL min^–1^) to yield geministatin A (**1**) (*t*_R_ 18.42 min; 203 mg). Fr 7 (430 mg) was purified by isocratic preparative HPLC (Zorbax C_18_, isocratic 90% MeCN/H_2_O containing 0.1% TFA, 20 mL min^–1^) to yield geministatin B (**2**) (*t*_R_ 24.68 min; 7.9 mg).

### Chemical degradation studies

#### Preparation of geministatin B (**2**)

A solution of geministatin A (**1**; 50 mg) in acetone (4 mL) was treated with aqueous HCl (10 M; 2 mL) and incubated at 25 °C for 24 h. The reaction mixture was diluted with H_2_O (50 mL), adsorbed onto C_18_ silica (5 g), washed with H_2_O (50 mL) and eluted with MeCN (50 mL). The MeCN eluate was purified by preparative HPLC (Zorbax C_18_; isocratic 100% MeCN, 20 mL min^–1^) to yield geministatin B (**2**; *t*_R_ 11.19 min; 20.0 mg, 40%).

#### Preparation of geministatin C (**3**)

A solution of geministatin A (**1**; 20 mg) in MeOH (2 mL) was heated at 80 °C in a sealed vial for 4 h. The reaction mixture was purified by preparative HPLC (Zorbax C_18_; isocratic 95% MeCN/H_2_O, 20 mL min^–1^) to yield geministatin C (**3**; *t*_R_ 10.60 min; 5.9 mg, 30%).

#### Preparation of geministatin D (**4**)

A solution of geministatin A (**1**; 20 mg) in 95% acetone/H_2_O (2 mL) was heated at 80 °C for 4 h. The reaction mixture was purified by preparative HPLC (Zorbax C_18_; isocratic 95% MeCN/H_2_O, 20 mL min^–1^) to yield geministatin D (**4**; *t*_R_ 8.69 min; 4.2 mg, 21%).

#### Preparation of geministatin E (**5**)

A solution of geministatin B (**2**; 20.0 mg) in MeOH (2 mL) was heated at 80 °C in a sealed vial for 30 min. The reaction mixture was purified by preparative HPLC (Zorbax C_18_; isocratic 100% MeCN, 20 mL min^–1^) to yield geministatin E (**5**; *t*_R_ 8.44 min; 6.8 mg, 34%).

#### Preparation of dehydromerulinic acid A (**6**)

A solution of geministatin B (**2**; 12.1 mg) in 95% acetone/H_2_O (2 mL) was heated at 80 °C in a sealed vial for 1 h. The reaction mixture was purified by preparative HPLC (Zorbax C_18_, isocratic 95% MeCN/H_2_O containing 0.01% TFA, 20 mL min^–1^) to yield dehydromerulinic acid A (**6**; *t*_R_ 11.41 min; 8.3 mg, 69%).

#### Ozonolysis of geministatin A (**1**)

Geministatin A (**1**; 150 mg) was dissolved in MeOH (40 mL) and ozone was bubbled through the solution for 3 min at a rate of 10 mg min^–1^. The reaction mixture was purified by preparative HPLC (Zorbax C_18_; isocratic 40% MeCN/H_2_O containing 0.01% TFA, 20 mL min^–1^) to yield geministatin A ozonolysis product (**7**; *t*_R_ 5.69 min; 11.7 mg, 7.8%).

### Description of physicochemical properties

#### Geministatin A (**1**)

White powder; [α]_D_^24^ ‒28.7 (*c* 1.00, MeOH); UV (MeCN) λ_max_ (log ε) 215 (4.49), 265 (4.14), 305 (3.86) nm; IR (ATR) ν_max_ 3673, 2987, 2883, 1795, 1634, 1529, 1385, 1219, 1162, 1112, 945, 905, 818, 644 cm^–1^; ^1^H and ^13^C NMR see Table 1 and Table S2. HR-ESI(‒)-MS *m/z* 699.3389; calcd for C_38_H_51_O_12_^‒^ [M ‒ H]^–^, 699.3386.

**Table 1:** ^1^H (600 MHz) and ^13^C (150 MHz) NMR data for **1** and **2** in DMSO-*d*_6_

Pos.	Geministatin A (**1**)		Geministatin B (**2**)	
	*δ*_C_, type	*δ*_H_, mult (*J* in Hz)	*δ*_C_, type	*δ*_H_, mult (*J* in Hz)
1	166.2, C		166.8, C	
2	113.0, C		109.2, C	
3	157.3, C		158.8, C	
3-OH		10.20, s		10.12, s
4	101.4, CH	6.46, d (2.2)	100.5, CH	6.23, d (2.1)
5	159.6, C		160.5, C	
5-OH				9.84, s
6	108.4, CH	6.43, d (2.2)	108.5, CH	6.17, d (2.1)
7	143.1, C		144.2, C	
8	33.5, CH_2_	2.60, m	33.9, CH_2_	2.59, m
9	30.9, CH_2_	1.54, m	31.0, CH_2_	1.52, m
10	28.6^a^, CH_2_	1.23–1.27^d^, m	28.9^b^, CH_2_	1.25^i^, m
11	29.0^a^, CH_2_	1.23–1.27^d^, m	29.0^b^, CH_2_	1.24–1.26^i^, m
12	28.7^a^, CH_2_	1.23–1.27^d^, m	28.6^c^, CH_2_	1.24–1.26^i^, m
13	28.7^a^, CH_2_	1.23–1.27^d^, m	28.7^c^, CH_2_	1.26^i^, m
14	26.6, CH_2_	1.98^e^, m	26.6, CH_2_	1.97^j^, m
15	129.7, CH	5.30^f^, m	129.7, CH	5.29^k^, m
16	127.7, CH	5.27^f^, m	127.7, CH	5.26^k^, m
17	25.2, CH_2_	2.70, br dd (6.7, 6.7)	25.2, CH_2_	2.70, br dd (6.5, 6.5)
18	127.7, CH	5.27^f^, m	127.7, CH	5.26^k^, m
19	129.7, CH	5.30^f^, m	129.7, CH	5.29^k^, m
20	26.6, CH_2_	1.98^e^, m	26.6, CH_2_	1.97^j^, m
21	28.9^a^, CH_2_	1.23–1.27^d^, m	28.7^b^, CH_2_	1.24–1.26^i^, m
22	30.8, CH_2_	1.20, m	30.9, CH_2_	1.20, m
23	21.9, CH_2_	1.23, m	21.9, CH_2_	1.23, m
24	13.9, CH_3_	0.82, t (6.9)	13.9, CH_3_	0.81, t (7.2)
1ʹ	170.6, C		170.6, C	
1ʹ-OH		13.32, br s		13.33, br s
2ʹ	116.6, C		116.2, C	
3ʹ	159.0, C		159.1, C	
3ʹ-OH		11.31, br s		11.12, br s
4ʹ	107.2, CH	6.58, d (2.2)	107.2, CH	6.57, d (2.3)
5ʹ	152.4, C		152.5, C	
6ʹ	114.4, CH	6.52, br d (2.2)	114.4, CH	6.52, dd (2.3, 0.6)
7ʹ	139.6, C		139.6, C	
8ʹ	21.0, CH_3_	2.36, s	21.0, CH_3_	2.36, br s
1″	100.6, CH	4.81^g^, d (7.8)		
2″	70.2, CH	3.54^h^, m		
3″	73.2, CH	3.41, dd (9.7, 3.2)		
4″	67.9, CH	3.71, br d (3.2)		
5″	75.4, CH	3.55^h^, m		
6″	60.0, CH_2_	3.56, m		
		3.48, m		
2″-OH		5.15, br s		
3″-OH		4.81^g^, br s		
4″-OH		4.49, br s		
6″-OH		4.63, br s		

^a–c^assignments interchangeable, ^d–k^overlapping resonances

#### Geministatin B (**2**)

White powder; UV (MeCN) λ_max_ (log ε) 215 (4.55), 265 (4.19), 305 (3.95) nm; IR (ATR) ν_max_ 3346, 3041, 2987, 2880, 2584, 1798, 1633, 1530, 1388, 1288, 1213, 1165, 1092, 939, 873, 820, 765, 639 cm^–1^; ^1^H and ^13^C NMR see Table 1 and Table S3; HR-ESI(‒)-MS *m/z* 537.2863; calcd for C_32_H_41_O_7_^‒^ [M ‒ H]^‒^, 537.2858.

#### Geministatin C (**3**)

White powder; [α]_D_^24^ ‒26.5 (*c* 1.00, MeOH); UV (MeCN) λ_max_ (log ε) 215 (3.99), 265 (3.63), 305 (3.20) nm; IR (ATR) ν_max_ 3036, 2986, 2878, 1801, 1682, 1633, 1598, 1487, 1354, 1286, 1230, 1196, 1152, 1115, 931, 822, 609 cm^–1^; ^1^H and ^13^C NMR see Table S4; HR-ESI(‒)-MS *m/z* 563.3224; calcd for C_31_H_47_O_9_^‒^ [M ‒ H]^‒^, 563.3226.

#### Geministatin D (**4**)

White powder; [α]_D_^24^ –34.7 (*c* 0.250, MeOH); UV (MeCN) λ_max_ (log ε) 215 (3.91), 265 (3.38), 305 (3.12) nm; IR (ATR) ν_max_ 3964, 3681, 3032, 2985, 2881, 1767, 1527, 1392, 1325, 1197, 1156, 1115, 1053, 928, 874, 822, 745, 653, 603 cm^–1^; ^1^H and ^13^C NMR see Table S5; HR-ESI(‒)-MS *m/z* 549.3075; calcd for C_30_H_45_O_9_^‒^ [M ‒ H]^‒^, 549.3069.

#### Geministatin E (**5**)

Pale yellow oil; UV (MeCN) λ_max_ (log ε) 215 (4.38), 265 (4.08), 305 (3.67) nm; IR (ATR) ν_max_ 3101. 2986, 2880, 1963, 1841, 1634, 1535, 1406, 1356, 1289, 1220, 1183, 1126, 1056, 981, 889, 784, 641 cm^–1^; ^1^H and ^13^C NMR see Table S6; HR-ESI(‒)-MS *m/z* 401.2702; calcd for C_25_H_37_O_4_^‒^ [M ‒ H]^‒^, 401.2697.

#### Ozonolysis Product (**7**)

White powder; [α]_D_^24^ ‒32.7 (*c* 0.500, MeOH); UV (MeCN) λ_max_ (log ε) 215 (4.68), 265 (4.33), 305 (4.06) nm; IR (ATR) ν_max_ 3679, 2984, 2891, 1771, 1632, 1523, 1388, 1217, 1159, 1109, 873, 819, 736, 645 cm^–1^; ^1^H and ^13^C NMR see Table S8; HR-ESI(‒)-MS *m/z* 639.2302; calcd for C_30_H_39_O_15_^‒^ [M ‒ H]^‒^, 639.2294.

## Results and discussion

*A. gemini* was cultivated on a range of agars [glycerol casein agar (GCA), Czapek’s agar (CZA), oatmeal agar (OMA), malt extract agar (MEA) and yeast extract sucrose agar (YES)] and grains [cracked wheat (BL), pearl barley (PB), jasmine rice (JR)] commonly used for cultivation of fungi with a broad saprophytic diet [16]. HPLC analysis of the cultures revealed consistent secondary metabolite profiles across the media (Fig. S1), with JR achieving the highest level of productivity (Table S1). LCMS analysis of the secondary metabolites produced by this fungus did not match any known actives in our in-house standards library, which contains >12,000 metabolites. The UV spectra of several peaks showed similarity with phanerosporic acid, previously reported as a metabolite with antibiotic activity produced by the wood rot fungus, *Phanerochaete chrysosporium* [17]. However, a comparison of the metabolite profiles of *A. gemini* and *P. chrysosporium* revealed differences in retention times, UV-vis spectra and molecular weights of the metabolites (Fig. S2), thus warranting further investigation. *A. gemini* was cultivated on jasmine rice (4.2 kg) for 14 d at 24 °C. The culture was then extracted with acetone and partitioned into ethyl acetate to give a crude extract, which was fractionated by silica gel chromatography and then reversed-phase preparative HPLC to yield geministatins A (**1**) and B (**2**).

HR-ESI( − )-MS analysis of **1** revealed a deprotonated molecule indicative of the molecular formula C_38_H_52_O_12_, requiring thirteen double bond equivalents (DBEs). The ^1^H, ^13^C and HSQC NMR data for **1** (Table 1 and Table S2) revealed two pairs of *meta*-coupled aromatic methine doublets (*δ*_H_ 6.46/6.43 and *δ*_H_ 6.58/6.52; *J* = 2.2 Hz) and twelve aromatic carbon resonances, suggesting the presence of two tetrasubstituted benzenes. The NMR data also revealed two pairs of almost identical olefinic methines (*δ*_H_ 5.30/5.27; *δ*_C_ 129.7/127.7), suggesting the presence of two unsubstituted double bonds. The presence of resonances attributable to six oxygenated carbons, including one putative anomeric carbon (*δ*_C_ 100.6, 75.4, 73.2, 70.2, 67.9, 60.0), suggested the presence of a hexopyranoside moiety. Two downfield ^13^C resonances (*δ*_C_ 166.2 and 170.6) attributable to ester/carboxylic acid carbonyl groups accounted for the final two DBE. Upfield resonances included an aromatic methyl singlet (*δ*_H_ 2.36, *δ*_C_ 21.0), twelve aliphatic methylenes and a terminal methyl triplet (*δ*_H_ 0.82, *J* = 6.9 Hz, *δ*_C_ 13.9). Taken together these data accounted for all carbon atoms, 45 of 52 protons, and the 13 DBE required by the chemical formula of **1**. The remaining seven ^1^H resonances (*δ*_H_ 13.32, 11.31, 10.20, 5.15, 4.81, 4.63, 4.49) were assigned as exchangeable protons using HSQC and ^1^H NMR data, thus confirming the chemical formula of **1**.

**Fig. 2:**
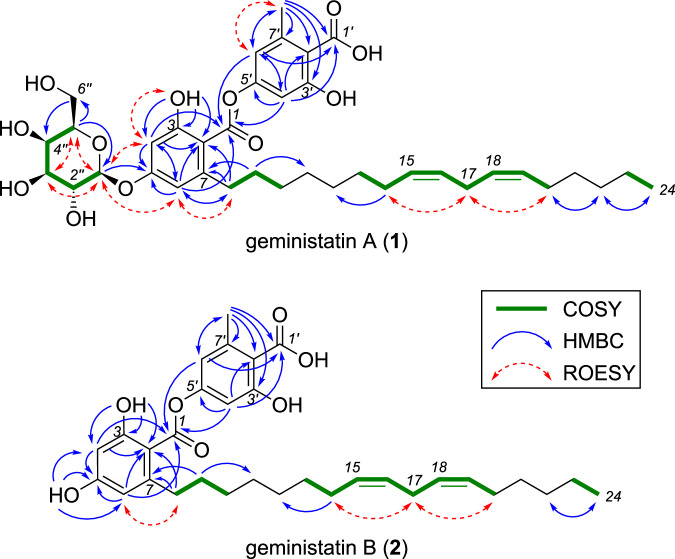
Key 2D NMR correlations for geministatins A (**1**) and B (**2**)

Detailed analysis of the HMBC, COSY and ROESY data for **1** (Fig. 2) confirmed the two tetrasubstituted benzenes were connected by an ester linkage, indicative of a depside. Long-range (^4^*J*_CH_) HMBC correlations from H-4 and H-6 to C-1 and from H-4′ and H-6′ to C-1′ confirmed the assignment of the ester and carboxylic acid carbons to their respective ring systems. HMBC correlations from the aromatic methyl H_3_-8′ to carboxylic acid carbonyl C-1′ and aromatic carbons C-2′, C-3′, C-6′ and C-7′, together with a ROESY correlation between H_3_-8′ and *meta*-coupled aromatic proton H-6′, confirmed the presence of an orsellinic acid subunit in **1**. An HMBC correlation from the anomeric proton H-1′′ to the oxygenated aromatic carbon C-5 and a ROESY correlation between H-1′′ and H-6 confirmed the locus of the hexopyranoside moiety on the central benzene ring, which was determined to be β-galactopyranoside based on the observed ^3^*J*_H-1′′,H-2′′_ diaxial coupling constant (7.8 Hz) and diagnostic ROESY correlations between H-1′′, H-3′′ and H-5′′. The C_17_ alkyl chain of **1** was confirmed to be attached to C-7 of the central benzene ring based on HMBC correlations from H_2_-8 to C-2, C-6 and C-7, and a ROESY correlation between H_2_-8 and H-6.

**Fig. 3:**
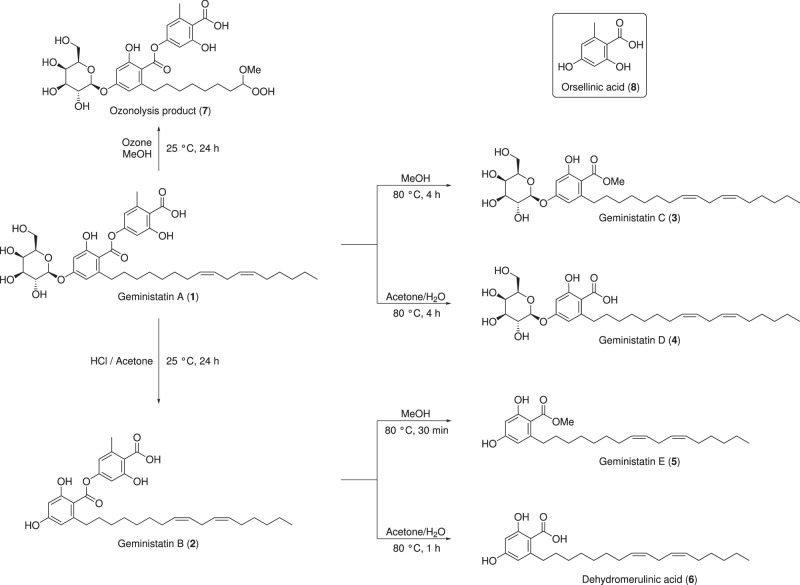
Chemical degradation of geministatins A (**1**) and B (**2**)

The ^1^H and ^13^C NMR resonances corresponding to the alkyl chain were overlapping within the methylene envelope and hence it was not possible to determine the absolute location of the two double bonds by NMR. However, the arrangement of the double bonds relative to each other was determined based on COSY correlations between the olefinic methine protons and their adjacent methylene protons. Two coincident olefinic methines, H-16 and H-18, with overlapping chemical shifts (*δ*_H_ 5.27, *δ*_C_ 127.7), both showed a COSY correlation to the same methylene group (*δ*_H_ 2.70; H_2_-17), suggesting they were separated by a single carbon. Similarly, olefinic protons H-15 and H-19, also coincidental (*δ*_H_ 5.30, *δ*_C_ 129.7), showed COSY correlations to the methylene protons at *δ*_H_ 1.98 (2 × 2H; H_2_-14 and H_2_-20). The geometries of the double bonds were determined to be *Z*,*Z* based on the ^13^C chemical shift of the C-17 methylene group (*δ*_C_ 25.2), which was compared with previously reported chemical shifts for the four possible geometric isomers 9*Z*,12*Z* (*δ*_C_ 25.6), *9Z,12E* (*δ*_C_ 30.5), *9E,12Z* (*δ*_C_ 30.5) and *9E*,12*E* (*δ*_C_ 35.7) of synthetic triacylglycerols of linoleic acid [18]. To determine the absolute position of the double bonds on the alkyl side chain, a solution of **1** in MeOH was treated with ozone as described by Criegee [19], which yielded the geminal methoxyhydroperoxide **7** (Fig. 3). This confirmed the position of the double bonds to be Δ^15,16^ and Δ^18,19^ and thus completed the structure elucidation of **1**.

Geministatin B (**2**) was isolated as a white powder. HR-ESI( − )-MS analysis of **2** revealed a deprotonated molecule indicative of the molecular formula C_32_H_42_O_7_. The ^1^H and ^13^C NMR data for **2** (Table 2 and Table S3) were almost identical to those for **1**, except for the absence of signals associated with the β-galactopyranose moiety and the presence of an additional phenolic proton 5-OH (*δ*_H_ 9.84). These observations, coupled with a mass deficit of C_6_H_10_O_5_ compared to **1**, suggested that **2** is the aglycone of **1**. This was confirmed by the presence of key HMBC correlations from 5-OH to C-4, C-5 and C-6.

**Table 2:** Bioassay results for compounds **1**–**6** and **8**

Compound	Minimum inhibitory concentration (MIC; µg mL^–1^)				IC_50_ (µM)	
	*B. subtilis*	*S. aureus*	MRSA	*S. cerevisiae*	NS-1	NFF
**1**	1.6	6.3	6.3	>200	71	>140
**2**	0.39	3.1	1.6	>200	93	>190
**3**	>100	>100	>100	>200	17	19
**4**	25	>100	>100	13	68	>180
**5**	13	>100	50	>200	8.7	47
**6**	0.2	3.1	0.78	>200	64	>260
**8**	25	>100	>100	>200	>590	>590
**Controls**^a^	6.3	3.1	>100	3.1	1.7	1.7

^a^Controls: *B. subtilis* ATCC 6633 = tetracycline; *S. aureus* ATCC 25923 and MRSA ATCC 33592 = ampicillin; *S. cerevisiae* ATCC 9763 = blasticidin S HCl; NS-1 ATCC TIB-18 and NFF TCC PCS-201) = sparsomycin

In an effort to expand upon the chemical space around the geministatin scaffold and to provide further analogues for bioactivity screening, we explored several methods for chemically degrading **1** to its component subunits (Fig. 3). Cleavage of the glycosidic linkage of **1** was effected with aqueous HCl in acetone, yielding **2**. Additionally, facile methanolysis and hydrolysis of the ester linkage in **1** and **2** was achieved by heating in MeOH or aqueous acetone, yielding novel geministatins C–E (**3**–**5**) as well as dehydromerulinic acid A (**6**), which has been previously reported as a metabolite of the wood-rotting basidiomycete *Hapalopilus mutans* [20]. The structures of **3**–**6** were confirmed by detailed spectroscopic analysis (Tables S4–S8).

### Biological screening

The geministatins and their degradation products were screened against the Gram-positive bacteria *B. subtilis*, *Staphylococcus aureus* and methicillin-resistant *S. aureus* (MRSA), the yeast *Saccharomyces cerevisiae* and mouse myeloma NS-1 and neonatal foreskin fibroblast (NFF) mammalian cell lines following the methods outlined previously [21] (Table 2). Geministatin A (**1**) showed strong activity against *B. subtilis* and moderate activity against *S. aureus* and MRSA, with no significant mammalian cytotoxicity. Interestingly, aglycone **2** showed increased antibacterial activity compared to **1**, with additional improvement in activity achieved following cleavage of the ester linkage to give **6**. Compounds **3**–**5** and monomeric subunit **8** showed no significant antibacterial activity.

The potent antibacterial activity and low mammalian cytotoxicity of the geministatins makes this class an attractive target for further investigation. Degradation of the core depside scaffold has shown that the improved MRSA activity is associated with loss of the β-galactopyranoside moiety. The broader activity of this class appears to be linked to the presence of an alkyl chain and a free carboxylic acid. Hydrolysis of the ester linkage of **2** to yield **6** retains antibacterial activity, while the absence of an alkyl side chain in **8** largely abolished antibacterial activity. The modular degradation of the parent geministatin scaffold, which is mediated by acid and heat, may point to a prodrug-like behaviour with ecological relevance to the fungus in exploiting its niche. *A. gemini* is only known from the type specimen isolated from rotting wood in tropical woodland in northern Australia. In similar habitats, bacteria and fungi represent fast growing invasive competitors for resources and secrete large amounts of esterases and glycosidases. For *A. gemini*, degradation of geministatins by other microbes may activate implicit biochemical defences against competition. Importantly, **8** forms a core structural motif of many fungal and lichen metabolites including other depsides such as lecanoric acid, umbilcaric acid and gyrophoric acid [22, 23]. While **8** lacks antibacterial activity, it acts as a common building block for more active metabolites. Phanerosporic acid, which is produced by the white wood rot fungus *Phanerochaete chrysosporium* [17], is a close analogue of dehydromerulinic acid A, the core building block of the geministatins. Further analysis of *A. gemini* is warranted to assess the full scope of the biological activity of this fungus.

## Supplementary information

Supporting Information
