# Supplementary material for: Geministatins: new depside antibiotics from the fungus Austroacremonium gemini
Source: J Antibiot (Tokyo). 2024 Jun 26;77(10):639–46. doi: 10.1038/s41429-024-00755-x (PMC11427298; doi:10.1038/s41429-024-00755-x)
Supplement: Supplementary file 1 — Supporting Information [file 41429_2024_755_MOESM1_ESM.pdf]

SUPPORTING INFORMATION

Geministatins: new depside antibiotics from the fungus *Austroacremonium gemini*

Andrew Crombie[1], John A. Kalaitzis[2], Rachel Chen[1], Daniel Vuong[1], Alastair Lacey[1],
Ernest Lacey[1,2], Roger G. Shivas[3,4], Yu Pei Tan[3,4], Nicolau Sbaraini[5],
Yit-Heng Chooi[5] and Andrew M. Piggott[2*]

[1] *Microbial Screening Technologies, Smithfield, NSW 2164, Australia*

[2] *School of Natural Sciences, Macquarie University, Sydney, NSW 2109, Australia*

[3] *Plant Pathology Herbarium, Department of Agriculture and Fisheries, Dutton Park, QLD 4102, Australia*

[4] *Centre for Crop Health, University of Southern Queensland, Toowoomba, QLD 4350, Australia*

[5] *School of Molecular Sciences, The University of Western Australia, Perth, WA 6009, Australia*

Table of Contents

Cultivation and Purification 3
NMR Data 7
UV-Vis Spectra 14
IR Spectra 16
HRMS Data 18
NMR Spectra 20

Supplementary Tables

Table S1. Yield of geministatin A (**1**) obtained after cultivation of *A. gemini* for 7 days on various media.... 4
Table S2. ^{1}H (600 MHz) and ^{13}C (150 MHz) NMR data for geministatin A (**1**) in DMSO-d_6........ 7
Table S3. ^{1}H (600 MHz) and ^{13}C (150 MHz) NMR data for geministatin B (**2**) in DMSO-d_6........ 8
Table S4. ^{1}H (600 MHz) and ^{13}C (150 MHz) NMR data for geministatin C (**3**) in DMSO-d_6........ 9
Table S5. ^{1}H (600 MHz) and ^{13}C (150 MHz) NMR data for geministatin D (**4**) in DMSO-d_6........ 10
Table S6. ^{1}H (600 MHz) and ^{13}C (150 MHz) NMR data for geministatin E (**5**) in DMSO-d_6........ 11
Table S7. ^{1}H (600 MHz) and ^{13}C (150 MHz) NMR data for dehydromerulinic acid (**6**) in DMSO-d_6........ 12
Table S8. ^{1}H (600 MHz) and ^{13}C (150 MHz) NMR data for geministatin A ozonolysis (**7**) in DMSO-d_6..... 13

Supplementary Figures

Figure S1. HPLC traces (254 nm) of extracts from *A. gemini* after cultivation for 7 days on various media.. 3
Figure S2. Comparison of HPLC traces (254 nm) and UV-vis spectra of geministatins A (**1**) and B (**2**) from *A. gemini* and phanerosporic acid from *P. chrysoporium* 5
Figure S3. Isolation scheme for natural products from *Austroacremonium gemini* MST-FP2131 6
Figure S4. UV-vis spectrum of geministatin A (**1**) in MeCN........ 14
Figure S5. UV-vis spectrum of geministatin B (**2**) in MeCN........ 14
Figure S6. UV-vis spectrum of geministatin C (**3**) in MeCN........ 14
Figure S7. UV-vis spectrum of geministatin D (**4**) in MeCN........ 15
Figure S8. UV-vis spectrum of geministatin E (**5**) in MeCN........ 15

* Corresponding author. Email: andrew.piggott@mq.edu.au

Figure S9. UV-vis spectrum of geministatin A ozonolysis product (**7**) in MeCN 15
Figure S10. IR spectrum (ATR) of geministatin A (**1**)........ 16
Figure S11. IR spectrum (ATR) of geministatin B (**2**)........ 16
Figure S12. IR spectrum (ATR) of geministatin C (**3**)........ 16
Figure S13. IR spectrum (ATR) of geministatin D (**4**)........ 17
Figure S14. IR spectrum (ATR) of geministatin E (**5**)........ 17
Figure S15. IR spectrum (ATR) of geministatin A ozonolysis product (**7**)........ 17
Figure S16. HR-ESI(–)-MS spectrum of geministatin A (**1**)........ 18
Figure S17. HR-ESI(–)-MS spectrum of geministatin B (**2**)........ 18
Figure S18. HR-ESI(–)-MS spectrum of geministatin C (**3**)........ 18
Figure S19. HR-ESI(–)-MS spectrum of geministatin D (**4**)........ 19
Figure S20. HR-ESI(–)-MS spectrum of geministatin E (**5**)........ 19
Figure S21. HR-ESI(–)-MS spectrum of geministatin A ozonlysis product (**7**)........ 19
Figure S22. ^{1}H NMR spectrum (600 MHz) of geministatin A (**1**) in DMSO-d_6........ 20
Figure S23. ^{13}C NMR spectrum (150 MHz) of geministatin A (**1**) in DMSO-d_6........ 21
Figure S24. HSQC NMR spectrum (600 MHz) of geministatin A (**1**) in DMSO-d_6........ 22
Figure S25. HMBC NMR spectrum (600 MHz) of geministatin A (**1**) in DMSO-d_6........ 23
Figure S26. COSY NMR spectrum (600 MHz) of geministatin A (**1**) in DMSO-d_6........ 24
Figure S27. ROESY NMR spectrum (600 MHz) of geministatin A (**1**) in DMSO-d_6........ 25
Figure S28. ^{1}H NMR spectrum (600 MHz) of geministatin B (**2**) in DMSO-d_6........ 26
Figure S29. ^{13}C NMR spectrum (150 MHz) of geministatin B (**2**) in DMSO-d_6........ 27
Figure S30. HSQC NMR spectrum (600 MHz) of geministatin B (**2**) in DMSO-d_6........ 28
Figure S31. HMBC NMR spectrum (600 MHz) of geministatin B (**2**) in DMSO-d_6........ 29
Figure S32. COSY NMR spectrum (600 MHz) of geministatin B (**2**) in DMSO-d_6........ 30
Figure S33. ROESY NMR spectrum (600 MHz) of geministatin B (**2**) in DMSO-d_6........ 31
Figure S34. ^{1}H NMR spectrum (600 MHz) of geministatin C (**3**) in DMSO-d_6........ 32
Figure S35. ^{13}C NMR spectrum (150 MHz) of geministatin C (**3**) in DMSO-d_6........ 33
Figure S36. HSQC NMR spectrum (600 MHz) of geministatin C (**3**) in DMSO-d_6........ 34
Figure S37. HMBC NMR spectrum (600 MHz) of geministatin C (**3**) in DMSO-d_6........ 35
Figure S38. COSY NMR spectrum (600 MHz) of geministatin C (**3**) in DMSO-d_6........ 36
Figure S39. ROESY NMR spectrum (600 MHz) of geministatin C (**3**) in DMSO-d_6........ 37
Figure S40. ^{1}H NMR spectrum (600 MHz) of geministatin D (**4**) in DMSO-d_6........ 38
Figure S41. ^{13}C NMR spectrum (150 MHz) of geministatin D (**4**) in DMSO-d_6........ 39
Figure S42. HSQC NMR spectrum (600 MHz) of geministatin D (**4**) in DMSO-d_6........ 40
Figure S43. HMBC NMR spectrum (600 MHz) of geministatin D (**4**) in DMSO-d_6........ 41
Figure S44. COSY NMR spectrum (600 MHz) of geministatin D (**4**) in DMSO-d_6........ 42
Figure S45. ROESY NMR spectrum (600 MHz) of geministatin D (**4**) in DMSO-d_6........ 43
Figure S46. ^{1}H NMR spectrum (600 MHz) of geministatin E (**5**) in DMSO-d_6........ 44
Figure S47. ^{13}C NMR spectrum (150 MHz) of geministatin E (**5**) in DMSO-d_6........ 45
Figure S48. HSQC NMR spectrum (600 MHz) of geministatin E (**5**) in DMSO-d_6........ 46
Figure S49. HMBC NMR spectrum (600 MHz) of geministatin E (**5**) in DMSO-d_6........ 47
Figure S50. COSY NMR spectrum (600 MHz) of geministatin E (**5**) in DMSO-d_6........ 48
Figure S51. ROESY NMR spectrum (600 MHz) of geministatin E (**5**) in DMSO-d_6........ 49
Figure S52. ^{1}H NMR spectrum (600 MHz) of geministatin A ozonolysis product (**7**) in DMSO-d_6........ 50
Figure S53. ^{13}C NMR spectrum (150 MHz) of geministatin A ozonolysis product (**7**) in DMSO-d_6........ 51
Figure S54. HSQC NMR spectrum (600 MHz) of geministatin A ozonolysis product (**7**) in DMSO-d_6........ 52
Figure S55. HMBC NMR spectrum (600 MHz) of geministatin A ozonolysis product (**7**) in DMSO-d_6........ 53
Figure S56. COSY NMR spectrum (600 MHz) of geministatin A ozonolysis product (**7**) in DMSO-d_6........ 54
Figure S57. ROESY NMR spectrum (600 MHz) of geministatin A ozonolysis product (**7**) in DMSO-d_6........ 55

Cultivation and Purification

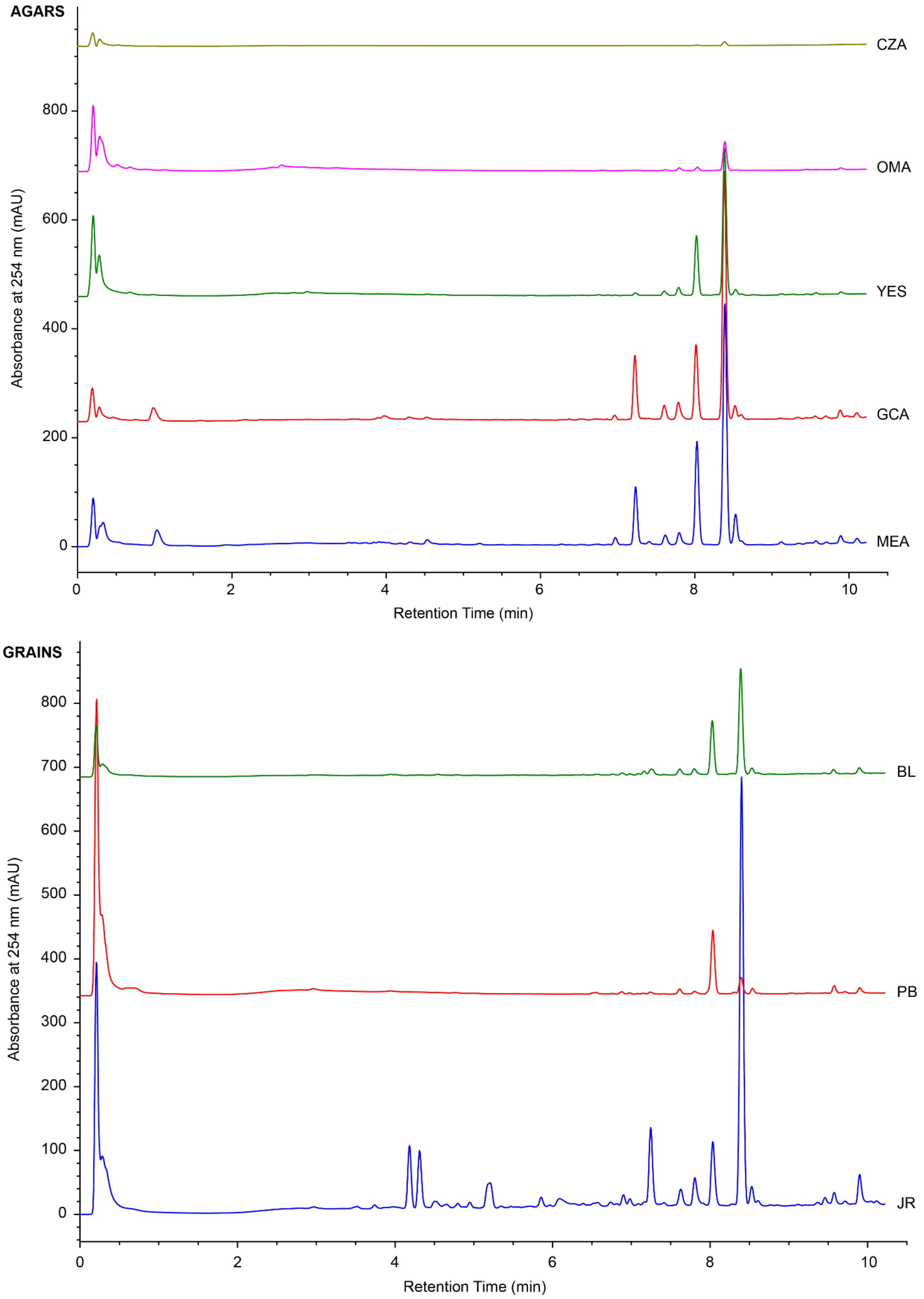

Figure S1. HPLC traces (254 nm) of extracts from *A. gemini* after cultivation for 7 days on various media

Table S1. Yield of geministatin A (**1**) obtained after cultivation of *A. gemini* for 7 days on various media

**Medium**	**Retention Time (min)**	**Absolute Peak Area (254 nm)**	**Relative Yield (%)**
**Agars**[a]			
Glycerol casein agar (GCA)	8.022	459	73
Czapek's agar (CZA)	8.025	2	0.32
Oatmeal agar (OMA)	8.035	20	3.2
**Malt extract agar (MEA)**	**8.034**	**626**	**100**
Yeast extract sucrose agar (YES)	8.024	347	55
**Grains**[b]			
Cracked wheat (BL)	8.033	273	79
Pearl barley (PB)	8.039	334	97
**Jasmine rice (JR)**	**8.041**	**346**	**100**

[a] Two agar discs (each 2 cm diameter) dissolved in methanol (2 mL)
[b] Grain (5 g) dissolved in methanol (10 mL)

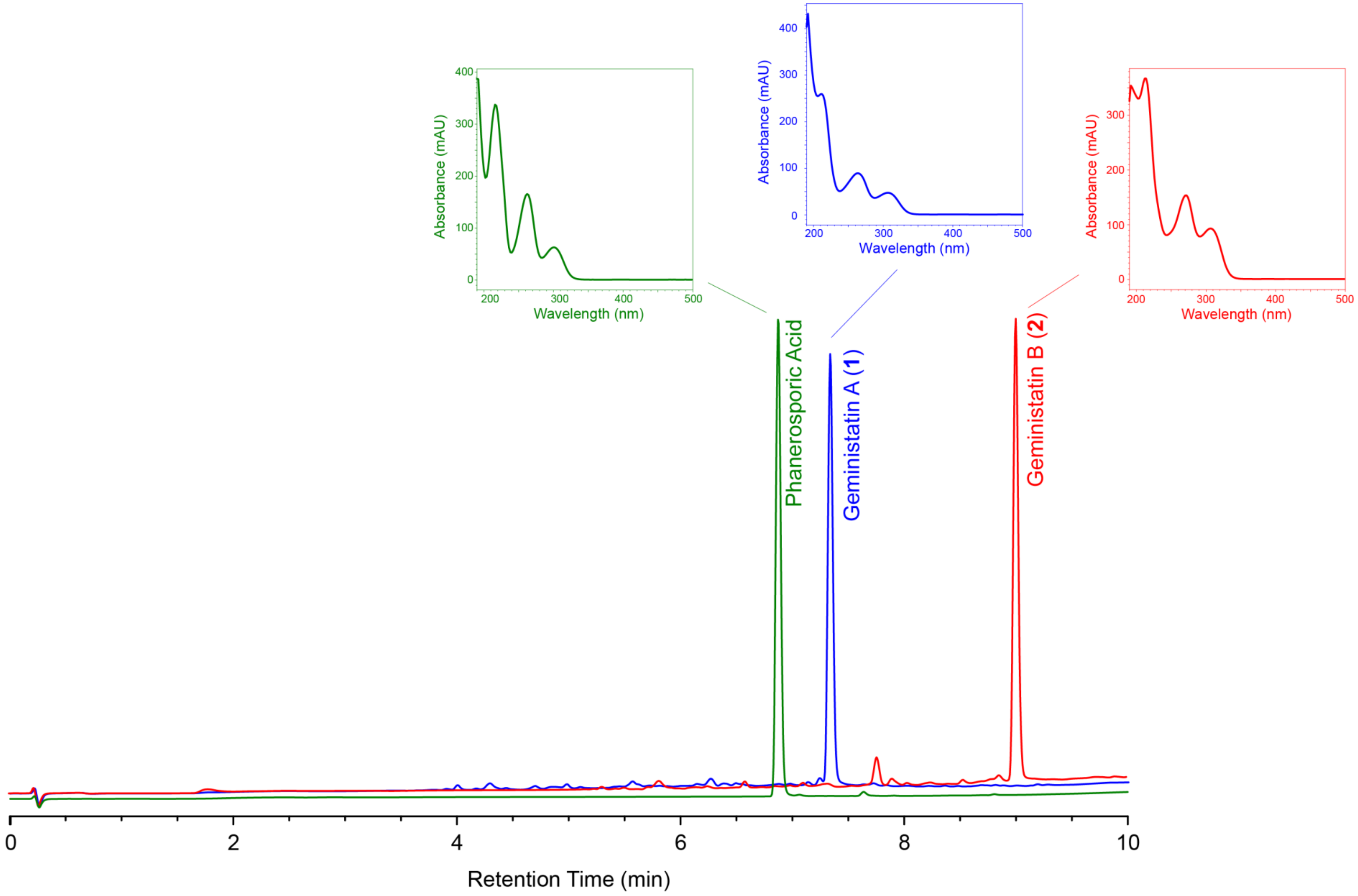

Figure S2. Comparison of HPLC traces (254 nm) and UV-vis spectra of geministatins A (**1**) and B (**2**) from *A. gemini* and phanerosporic acid from *P. chrysoporium*

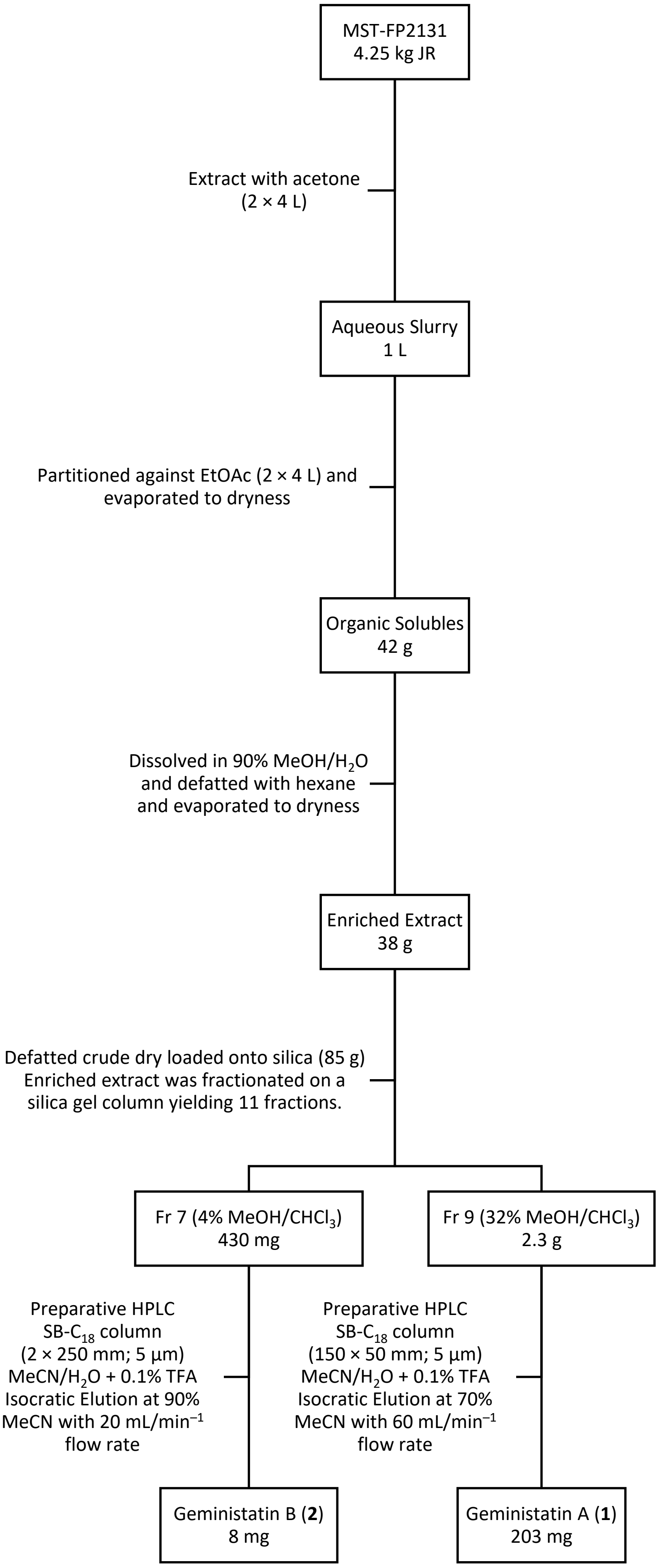

Figure S3. Isolation scheme for natural products from *Austroacremonium gemini* MST-FP2131

NMR Data

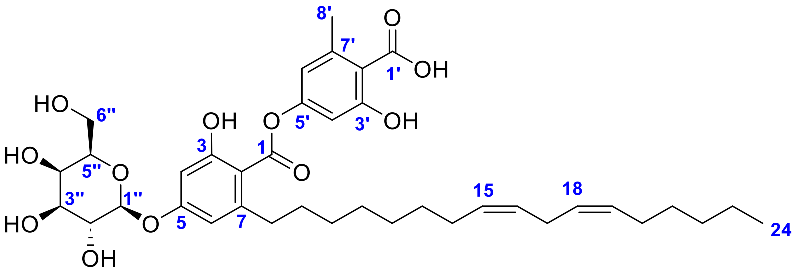

Table S2. ^{1}H (600 MHz) and ^{13}C (150 MHz) NMR data for geministatin A (**1**) in DMSO-d_6

Pos.	δ_C, type	δ_H, mult (*J* in Hz)	HMBC	COSY	ROESY
1	166.2, C				
2	113.0, C				
3	157.3, C				
3-OH		10.20, s	2, 3, 4		4
4	101.4, CH	6.46, d (2.2)	1, 2, 5, 6	6	3-OH, 1″
5	159.6, C				
6	108.4, CH	6.43, d (2.2)	1, 2, 4, 5, 8	4	8, 9, 1″
7	143.1, C				
8	33.5, CH_2	2.60, m	2, 6, 7, 9	9	6, 9
9	30.9, CH_2	1.54, m	7, 8, 10, 11	8, 10	6, 8
10	28.6[a], CH_2	1.23–1.27[b], m		9	
11	29.0[a], CH_2	1.23–1.27[b], m			
12	28.7[a], CH_2	1.23–1.27[b], m			
13	28.7[a], CH_2	1.23–1.27[b], m		14	
14	26.6, CH_2	1.98[c], m	12, 15, 16	13, 15	17
15	129.7, CH	5.30[d], m	17	14, 16	
16	127.7, CH	5.27[d], m	14	15, 17	
17	25.2, CH_2	2.70, br dd (6.7, 6.7)	15, 16, 18, 19	16, 18	14, 20
18	127.7, CH	5.27[d], m	20	17, 19	
19	129.7, CH	5.30[d], m	17	18, 20	
20	26.6, CH_2	1.98[c], m	18, 19, 22	19	17
21	28.9[a], CH_2	1.23–1.27[b], m			
22	30.8, CH_2	1.20, m	24		
23	21.9, CH_2	1.23, m		24	
24	13.9, CH_3	0.82, t (6.9)	22, 23	23	
1′	170.6, C				
1′-OH		13.32, br s			
2′	116.6, C				
3′	159.0, C				
3′-OH		11.31, br s			
4′	107.2, CH	6.58, d (2.2)	1, 1′, 2′, 3′, 5′, 6′	6′	
5′	152.4, C				
6′	114.4, CH	6.52, br d (2.2)	1, 1′, 2′, 3′, 4′, 5′, 8′	4′	8′
7′	139.6, C				
8′	21.0, CH_3	2.36, s	1′, 2′, 3′, 6′, 7′		
1″	100.6, CH	4.81[e], d (7.8)	5	2″	4, 6, 6′
2″	70.2, CH	3.54, m	1″, 3″	1″, 3″	3″, 5″
3″	73.2, CH	3.41, dd (9.7, 3.2)		2″, 4″	1″, 4″, 5″
4″	67.9, CH	3.71, br d (3.2)	2″, 3″	3″	3″, 5″
5″	75.4, CH	3.55[f], m	1″, 6″		1″, 3″, 4″
6″	60.0, CH_2	3.56[f], m	4″,	6″a	6″a
		3.48, m	5″,	6″b	6″b
2″-OH		5.15, br s			
3″-OH		4.81[e], br s			
4″-OH		4.49, br s			
6″-OH		4.63, br s			

[a] assignments interchangeable; [c–f] overlapping ^{1}H resonances.

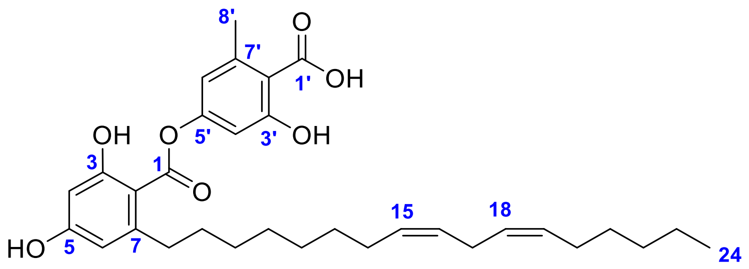

Table S3. ^{1}H (600 MHz) and ^{13}C (150 MHz) NMR data for geministatin B (**2**) in DMSO-d_6

Pos.	δ_C, type	δ_H, mult (*J* in Hz)	HMBC	COSY	ROESY
1	166.8, C				
2	109.2, C				
3	158.8, C				
3-OH		10.12, s	2, 3, 4		
4	100.5, CH	6.23, d (2.1)	1, 2, 3, 5, 6		
5	160.5, C				
5-OH		9.84, s	4, 5, 6		
6	108.5, CH	6.17, d (2.1)	1, 2, 4, 5, 8		8
7	144.2, C				
8	33.9, CH_2	2.59, m	6, 7, 9, 10	9	6, 9
9	31.0, CH_2	1.52, m	8, 10, 11	8, 10	8
10	28.9[a], CH_2	1.25[c], m		9	
11	29.0[a], CH_2	1.24–1.26[c], m			
12	28.6[b], CH_2	1.24–1.26[c], m			
13	28.7[b], CH_2	1.26[c], m			
14	26.6, CH_2	1.97[d], m	12, 13, 15, 16	15	17
15	129.7, CH	5.29[e], m	14	14	
16	127.7, CH	5.26[e], m	14		
17	25.2, CH_2	2.70, br dd (6.5, 6.5)	15, 16, 18, 19		14, 20
18	127.7, CH	5.26[e], m	14		
19	129.7, CH	5.29[e], m	14	20	
20	26.6, CH_2	1.97[d], m	18, 19, 21, 22	19	17
21	28.7[b], CH_2	1.24–1.26[c], m			
22	30.9, CH_2	1.20, m			
23	21.9, CH_2	1.23[c], m		24	
24	13.9, CH_3	0.81, t (7.2)	22, 23	23	
1′	170.6, C				
1′-OH		13.33, br s			
2′	116.2, C				
3′	159.1, C				
3′-OH		11.12, br s			
4′	107.2, CH	6.57, d (2.3)	1′, 2′, 3′, 5′, 6′		
5′	152.5, C				
6′	114.4, CH	6.52, dd (2.3, 0.6)	2′, 4′, 5′, 8′	8′	8′
7′	139.6, C				
8′	21.0, CH_3	2.36, br s	2′, 6′, 7′	6′	6′

[a–b] assignments interchangeable; [c–e] overlapping ^{1}H resonances.

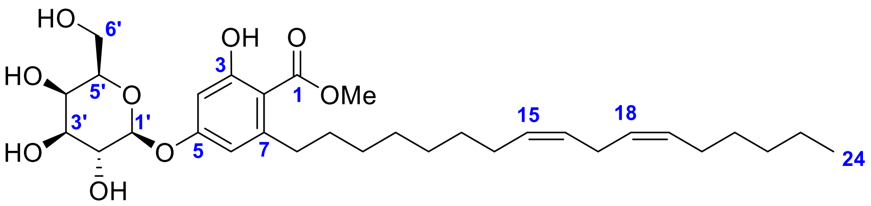

Table S4. ^{1}H (600 MHz) and ^{13}C (150 MHz) NMR data for geministatin C (**3**) in DMSO-d_6

Pos.	δ_C, type	δ_H, mult (*J* in Hz)	HMBC	COSY	ROESY
1	169.1, C				
1-OCH$_3$	51.6, CH$_3$	3.73, s	1		
2	113.7, C				
3	158.4, C				
3-OH		[b]			
4	101.5, CH	6.35, d (2.3)	1, 2, 3, 5, 6		1′
5	159.3, C				
6	107.5, CH	6.28, d (2.3)	1, 2, 3, 4, 5, 8		8, 9, 1′
7	142.8, C				
8	33.7, CH$_2$	2.47, m	2, 6, 7, 9, 10	9	6, 10
9	30.8, CH$_2$	1.44, m	7, 9, 11	8, 10	6
10	29.0, CH$_2$	1.24[c], m		9	8
11	28.7[a], CH$_2$	1.21–1.26[c], m			
12	28.9[a], CH$_2$	1.21–1.26[c], m			
13	28.6[a], CH$_2$	1.28–1.29[d], m		14	
14	26.6, CH$_2$	2.00, m	12, 13, 15, 16, 17	13, 15	15, 17
15	129.7, CH	5.33[e], m	13, 16	14	14
16	127.7, CH	5.29[e], m	14, 17, 18		17
17	25.2, CH$_2$	2.72, m	15, 16, 18, 19		14, 16, 18, 20
18	127.7, CH	5.29[e], m	16, 20		17
19	129.7, CH	5.33[e], m	17, 20	20	20
20	26.6, CH$_2$	2.00, m	17, 18, 19, 21, 22	19, 21	17, 20
21	28.6[a], CH$_2$	1.28–1.29[d], m		20	
22	30.9, CH$_2$	1.21–1.26[c], m			
23	21.9, CH$_2$	1.25[c], m		24	
24	13.9, CH$_3$	0.85, t (7.0)	22, 23	23	
1′	100.5, CH	4.75, d (7.7)	5, 2′	2′	4, 6, 3′, 5′
2′	70.2, CH	3.51[f], m	1′	1′, 3′	
3′	73.3, CH	3.38, dd (9.5, 3.3)	2′	2′, 4′	1′
4′	67.9, CH	3.69, br d (3.3)	2′, 3′	3′	
5′	75.4, CH	3.52[f], m	1′, 4′, 6′		1′
6′	60.1, CH$_2$	3.53[f], m			
		3.45, dd (9.5, 5.1)	4′, 5′		
2′-OH		[b]			
3′-OH		[b]			
4′-OH		[b]			
6′-OH		[b]			

[a] assignments interchangeable; [b] not observed; [c–f] overlapping ^{1}H resonances.

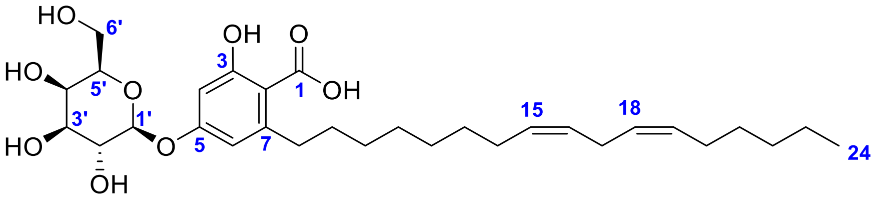

Table S5. ^{1}H (600 MHz) and ^{13}C (150 MHz) NMR data for geministatin D (**4**) in DMSO-d_6

Pos.	δ_C, type	δ_H, mult (*J* in Hz)	HMBC	COSY	ROESY
1	171.9, C				
1-OH		13.42, br s			
2	109.2, C				
3	161.7, C				
3-OH		11.48, br s			
4	101.1, CH	6.39, d (2.1)	1, 2, 3, 5, 6	6	1′
5	160.3, C				
6	109.9, CH	6.35, d (2.1)	1, 2, 4, 5, 8	4	1′, 8, 9
7	145.8, C				
8	34.9, CH_2	2.72, m	2, 6, 7, 9	9	6, 10
9	31.2, CH_2	1.48, m	7, 8, 10, 11	8, 10	
10	29.0[a], CH_2	1.26[b], m	8	9	6, 8
11	28.6[a], CH_2	1.22–1.27[b], m			
12	28.7[a], CH_2	1.22–1.27[b], m			
13	28.7, CH_2	1.28[b], m	15	14	
14	26.6, CH_2	2.00, m	15, 16	13, 15	15
15	129.7, CH	5.32, m	14, 17	14, 16	14
16	127.7, CH	5.29, m	14, 16, 17, 18	15, 17	17
17	25.2, CH_2	2.71, m	14, 15, 16, 18, 19, 20	16, 18	16, 18
18	127.7, CH	5.29, m	17, 20	17, 19	17
19	129.7, CH	5.32, m	16, 17, 20	18, 20	20
20	26.6, CH_2	2.00, m	18, 19	19, 21	19
21	29.1[a], CH_2	1.28[b], m	19	20	
22	30.9, CH_2	1.23, m	23		
23	21.9, CH_2	1.25, m	23	24	
24	13.9, CH_3	0.83, t (7.0)	22, 23	23	
1′	100.3, CH	4.83[c], d (7.6)	5, 2′, 5′	2′	4, 6
2′	70.3, CH	3.53[d], m	1′, 3′, 4′	1′, 3′, 2′-OH	
3′	73.2, CH	3.39, dd (9.5, 3.2)	2′	2′, 4′	
4′	68.0, CH	3.69, br d (3.2)	2′, 3′	3′, 5′, 4′-OH	
5′	75.5, CH	3.56, m	1′, 3′, 4′, 6′	5′	
6′	60.2, CH_2	3.53[d], m			
		3.46, m	4′, 5′	6′-OH	
2′-OH		5.12, br s		2′	
3′-OH		4.83[c], br s	3′	3′	
4′-OH		4.47, br s		4′	
6′-OH		4.61, br s		6′b	

[a] assignments interchangeable; [b–d] overlapping ^{1}H resonances.

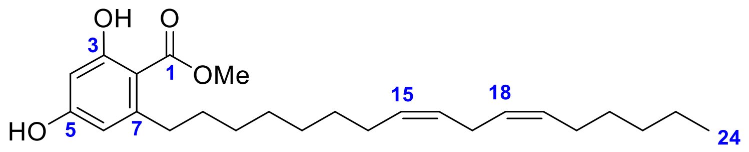

Table S6. ^{1}H (600 MHz) and ^{13}C (150 MHz) NMR data for geministatin E (**5**) in DMSO-d_6

Pos.	δ_C, type	δ_H, mult (*J* in Hz)	HMBC	COSY	ROESY
1	169.5, C				
1-OMe	51.6, CH_3	3.74, s	1		
2	109.0, C				
3	159.3, C				
3-OH		10.25[b], br s			
4	100.4, CH	6.14, d (2.3)	1, 2, 3, 5, 6	6	
5	160.3, C				
5-OH		9.77[b], br s			
6	108.6, CH	6.11, d (2.3)	1, 2, 4, 5, 8	4	8, 9
7	144.4, C				
8	34.2, CH_2	2.51, m	2, 6, 7, 9, 10	9	6, 10
9	30.9, CH_2	1.42, br m	7, 8, 10, 11	8, 10	6
10	29.0, CH_2	1.23–1.26[c], m		9	8
11	28.6[a], CH_2	1.23–1.26[c], m			
12	28.5[a], CH_2	1.23–1.26[c], m			
13	28.6[a], CH_2	1.29[c], m	11, 12, 14, 15	14	15
14	26.6, CH_2	2.00[d], dt (7.0, 6.9)	15, 16	13, 15	17
15	129.7, CH	5.30[e], m	14, 17	14, 16	13
16	127.7, CH	5.29[e], m	18	15, 17	
17	25.2, CH_2	2.72, t, (6.8)	15, 16, 18, 19	16, 18	14, 20
18	127.7, CH	5.29[e], m	16	17, 19	
19	129.7, CH	5.30[e], m	17, 20	18, 20	21
20	26.6, CH_2	2.00[d], dt (7.0, 6.9)	18, 19, 22	19, 21	17, 22
21	28.9, CH_2	1.24–1.29[c], m	19, 22, 23	20	19
22	30.9, CH_2	1.23[c], m	23		20
23	21.9, CH_2	1.25[c], m		24	
24	13.9, CH_3	0.84, t (7.1)	22, 23	23	

[a–b] assignments interchangeable; [c–e] overlapping ^{1}H resonances.

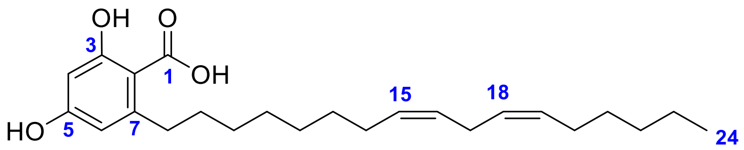

Table S7. ^{1}H (600 MHz) and ^{13}C (150 MHz) NMR data for dehydromerulinic acid (**6**) in DMSO-d_6

Pos.	δ_C, type	δ_H, mult (*J* in Hz)	HMBC	COSY	ROESY
1	172.7, C				
1-OH		13.31, br s			
2	105.1, C				
3	163.6, C				
3-OH		11.81, s			
4	100.5, CH	6.11, d (2.4)	1, 2, 3, 5		6
5	161.6, C				
5-OH		10.01, s	4, 5, 6		
6	110.0, CH	6.14, d (2.4)	1, 2, 5, 8		5-OH, 8
7	147.1, C				
8	35.3, CH_2	2.72[c], m	2, 6 ,7, 9, 10	9	6
9	31.3, CH_2	1.46, m		8, 10	
10	29.0[a], CH_2	1.20–1.29[d], m		9	
11	28.6[b], CH_2	1.20–1.29[d], m			
12	28.7[b], CH_2	1.20–1.29[d], m			
13	28.6[b], CH_2	1.28[d], m			15
14	26.7, CH_2	2.00[e], m	15, 16	15	17
15	129.7, CH	5.33[f], m	14, 17	14, 16	13
16	127.7, CH	5.29[f], m	14, 17, 18	15, 17	
17	25.2, CH_2	2.72[c], m	15, 16, 18, 19	16, 18	14, 20
18	127.7, CH	5.29[f], m	16, 17, 20	17, 19	
19	129.7, CH	5.33[f], m	17, 20	18, 20	21
20	26.6, CH_2	2.00[e], m	18, 19, 22	19, 21	17
21	29.1[a], CH_2	1.28[d], m		20	19
22	30.9, CH_2	1.23[d], m			
23	21.9, CH_2	1.27[d], m		24	
24	13.9, CH_3	0.83, t (6.9)	22, 23	23	

[a–b] assignments interchangeable; [c–f] overlapping ^{1}H resonances.

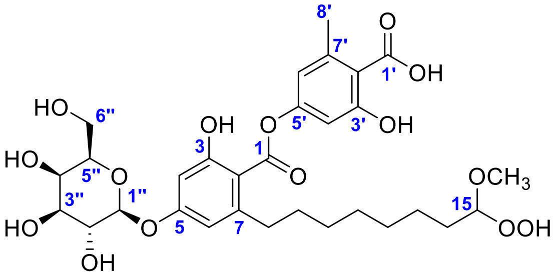

Table S8. ^{1}H (600 MHz) and ^{13}C (150 MHz) NMR data for geministatin A ozonolysis (**7**) in DMSO-d_6

Pos.	δ_C, type	δ_H, mult (J in Hz)	HMBC	COSY	ROESY
1	166.3, C				
2	113.1, C				
3	157.2, C				
3-OH		10.19, s	2, 3, 4		
4	101.4, CH	6.46, d (2.1)	1, 2, 3, 5, 6		1″
5	159.6[a], C				
6	108.7, CH	6.43, d (2.1)	2, 4, 5, 8		8, 9, 1″
7	143.1, C				
8	33.5, CH_2	2.60, m	2, 6, 7, 9, 10	9	6, 10
9	30.8, CH_2	1.54, m	7, 8, 11	8, 10	6
10	28.8[b], CH_2	1.27[c], m		9	8
11	28.8[b], CH_2	1.22–1.27[c], m			
12	28.8[b], CH_2	1.22–1.27[c], m			
13	24.3, CH_2	1.25[c], m			
14	31.3, CH_2	1.51, m		15	
		1.43, m		15	
15	107.5, CH	4.58, t (7.6)	13, 15-OMe	14a, 14b	
15-OMe	55.2, CH_3	3.33, s	15		
15-OOH		10.20, s			
1′	170.6, C				
1′-OH		[e]			
2′	116.9, C				
3′	159.6[a], C				
3′-OH		11.44, s			
4′	107.1, CH	6.53, br s	2′, 5′, 6′	6′	
5′	152.1, C				
6′	114.0, CH	6.47, br s	2′, 4′, 5′, 8′	4′	8′
7′	139.8, C				
8′	21.2, CH_3	2.38, s	2′, 6′, 7′		6′
1″	100.5, CH	4.81, d (7.6)	5, 2″, 3″, 5″	2″	4, 6, 3″
2″	70.2, CH	3.55[d], m		1″, 3″, 2″-OH	
3″	73.2, CH	3.41, dd (9.5, 2.8)		2″, 4″, 3″-OH	1″
4″	67.9, CH	3.71, br s	2″, 3″	3″, 4″-OH	5″
5″	75.4, CH	3.55[d], m			4″
6″	60.0, CH_2	3.48, m		6″-OH	
		3.55[d], m		6″-OH	
2″-OH		5.15, br s		2″	
3″-OH		4.84, m		3″	
4″-OH		4.49, br s		4″	
6″-OH		4.63, br s		6″a, 6″b	

[a] overlapping ^{13}C resonances; [b] assignments interchangeable; [c–d] overlapping ^{1}H resonances; [e] not observed.

UV-Vis Spectra

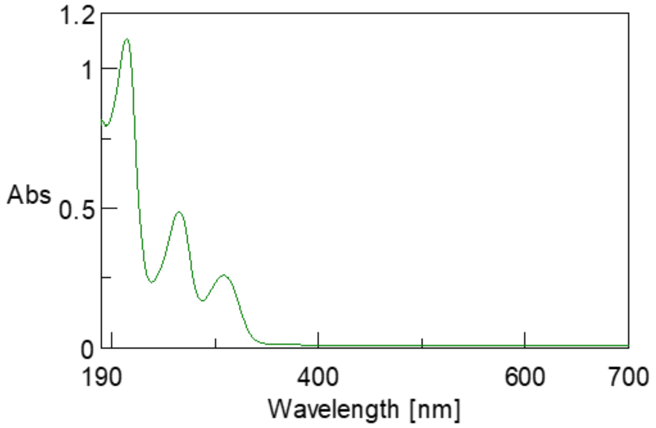

Figure S4. UV-vis spectrum of geministatin A (**1**) in MeCN

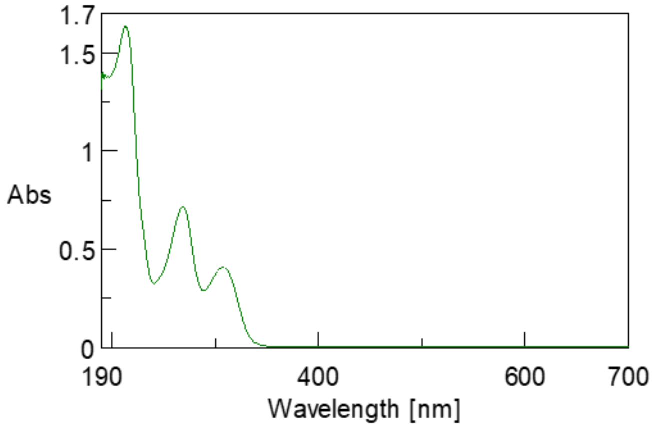

Figure S5. UV-vis spectrum of geministatin B (**2**) in MeCN

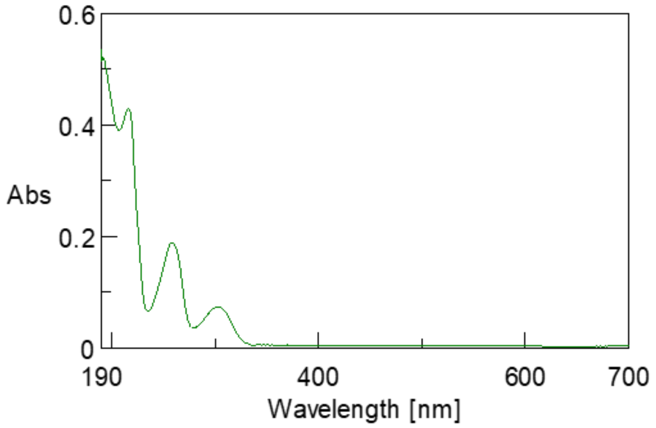

Figure S6. UV-vis spectrum of geministatin C (**3**) in MeCN

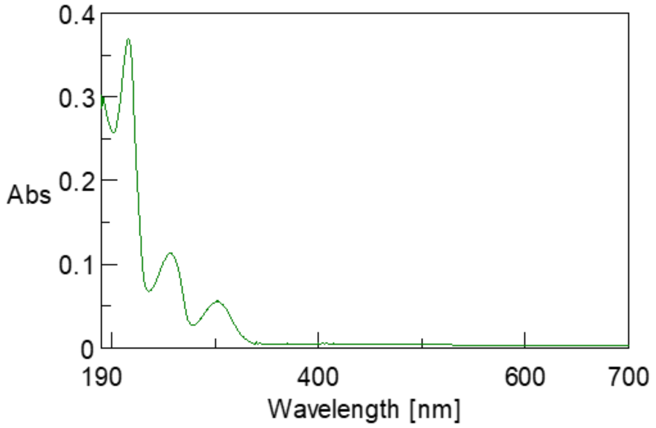

Figure S7. UV-vis spectrum of geministatin D (**4**) in MeCN

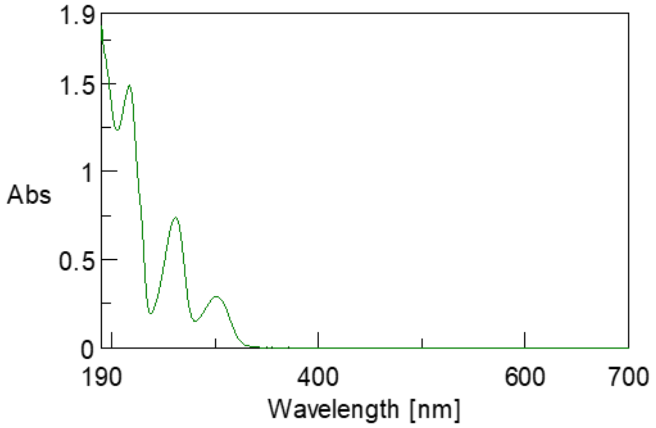

Figure S8. UV-vis spectrum of geministatin E (**5**) in MeCN

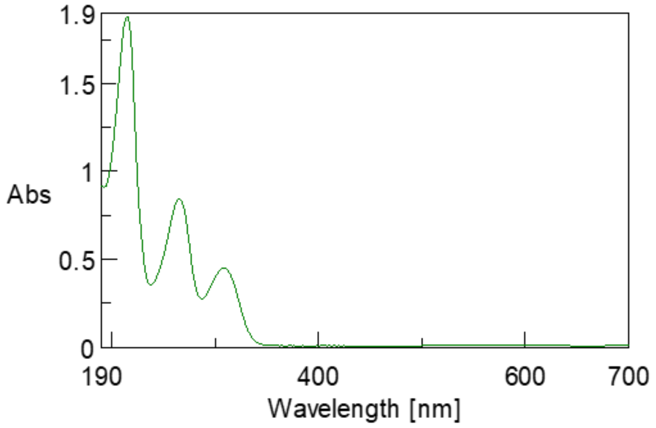

Figure S9. UV-vis spectrum of geministatin A ozonolysis product (**7**) in MeCN

IR Spectra

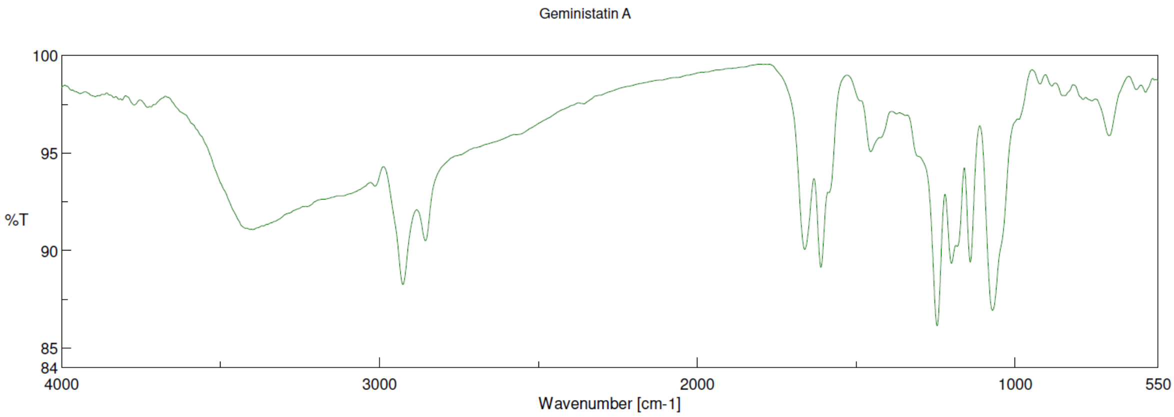

Figure S10. IR spectrum (ATR) of geministatin A (**1**)

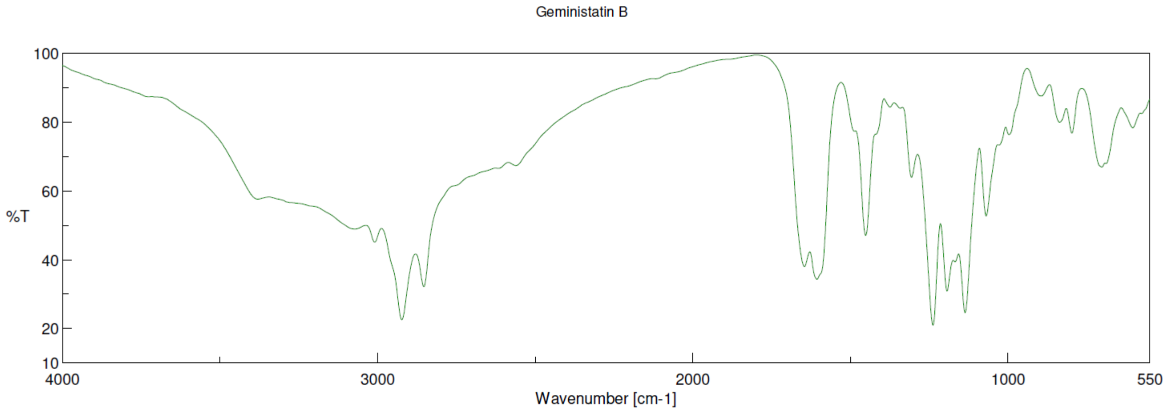

Figure S11. IR spectrum (ATR) of geministatin B (**2**)

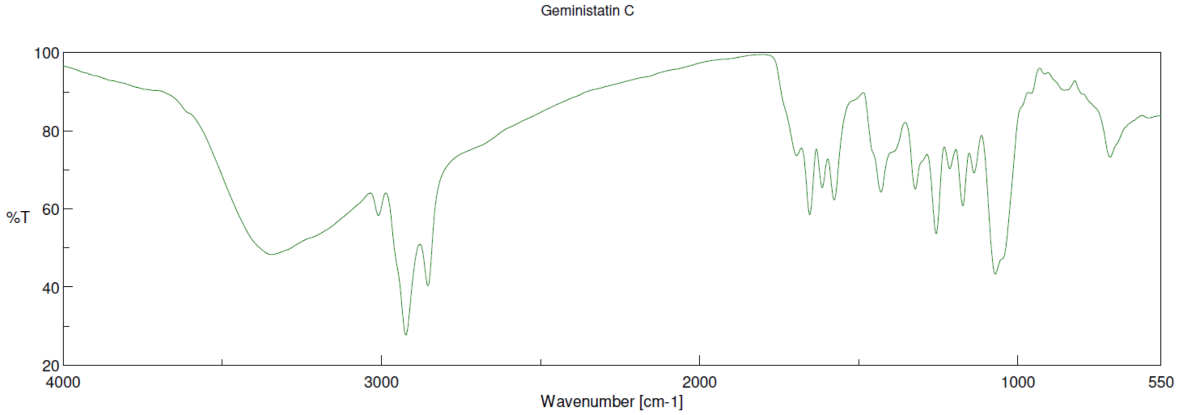

Figure S12. IR spectrum (ATR) of geministatin C (**3**)

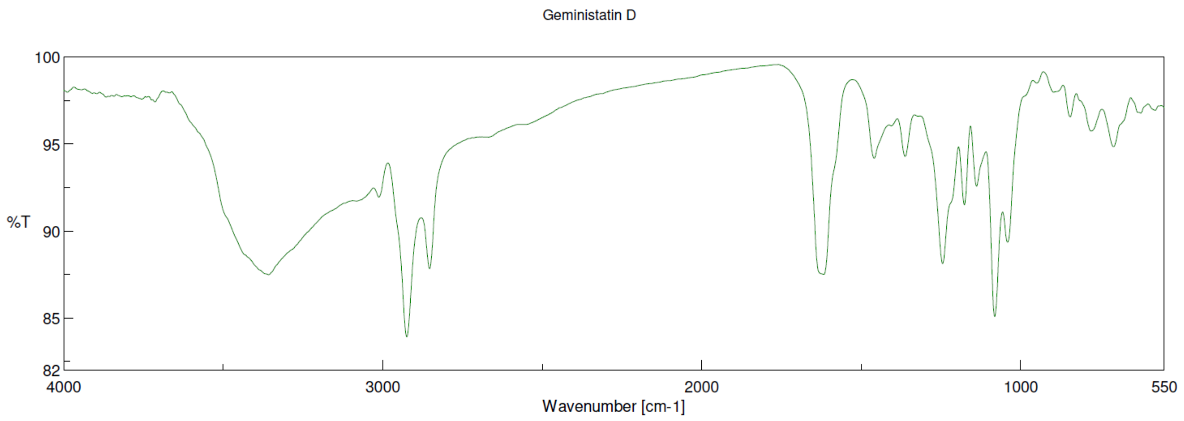

Figure S13. IR spectrum (ATR) of geministatin D (**4**)

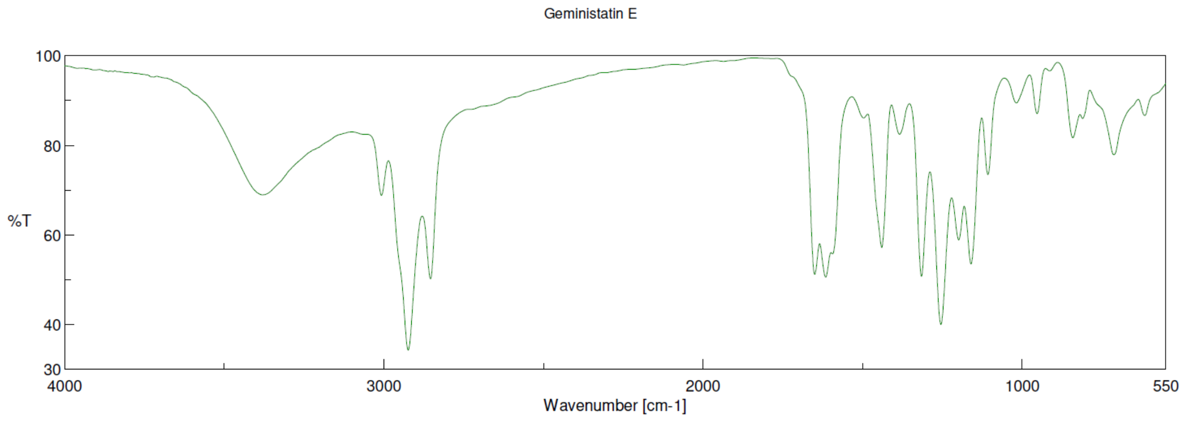

Figure S14. IR spectrum (ATR) of geministatin E (**5**)

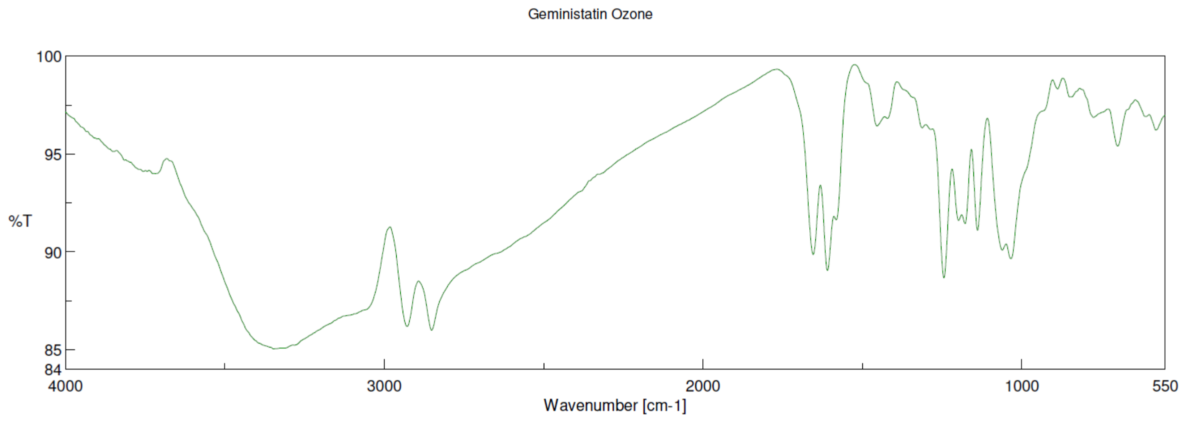

Figure S15. IR spectrum (ATR) of geministatin A ozonolysis product (**7**)

HRMS Data

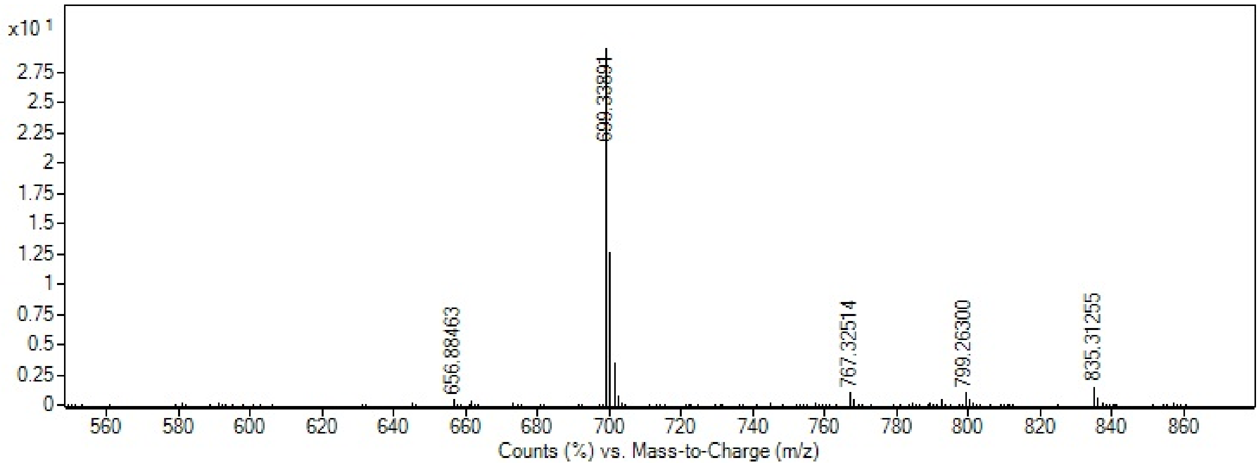

Figure S16. HR-ESI(–)-MS spectrum of geministatin A (**1**)

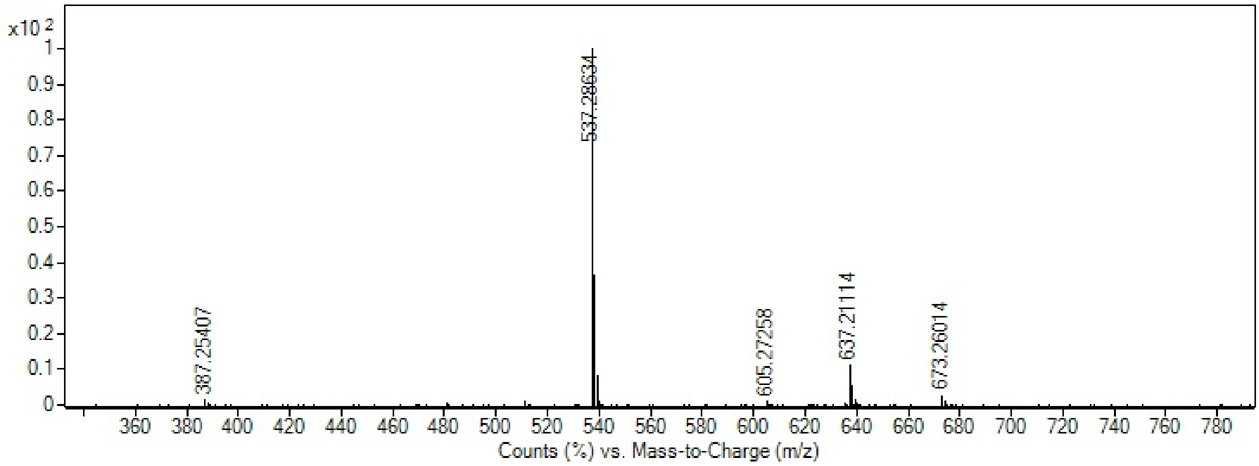

Figure S17. HR-ESI(–)-MS spectrum of geministatin B (**2**)

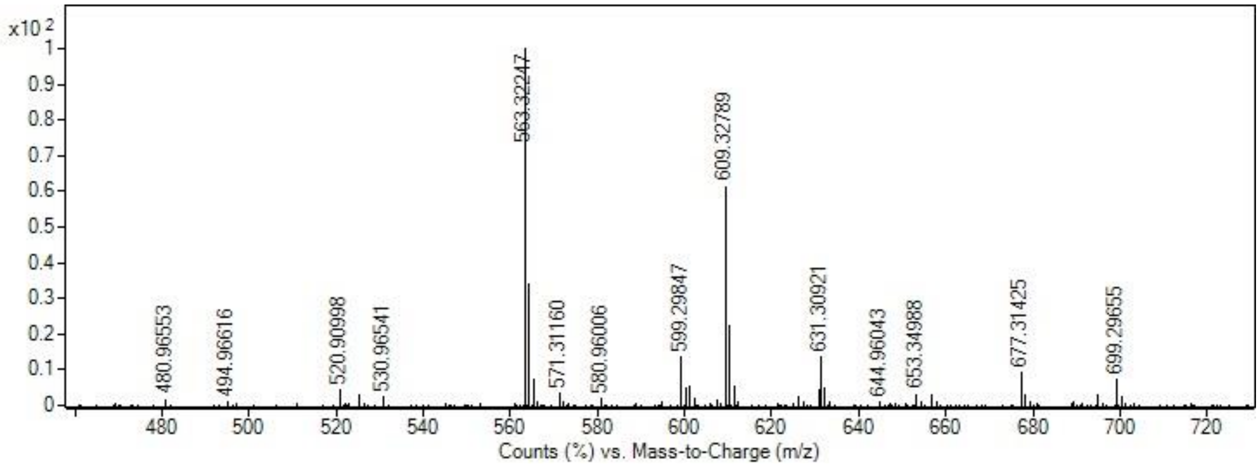

Figure S18. HR-ESI(–)-MS spectrum of geministatin C (**3**)

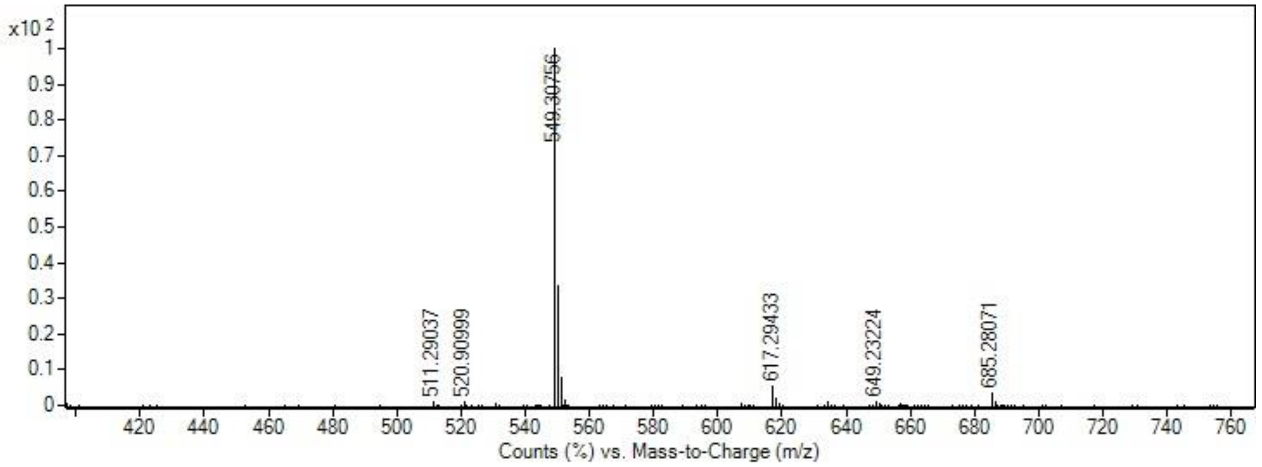

Figure S19. HR-ESI(–)-MS spectrum of geministatin D (**4**)

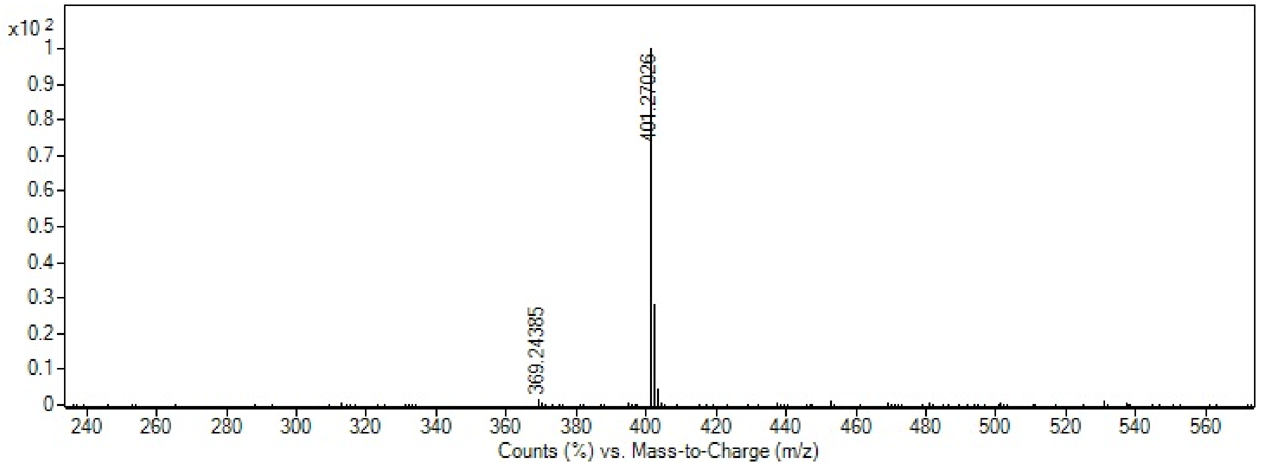

Figure S20. HR-ESI(–)-MS spectrum of geministatin E (**5**)

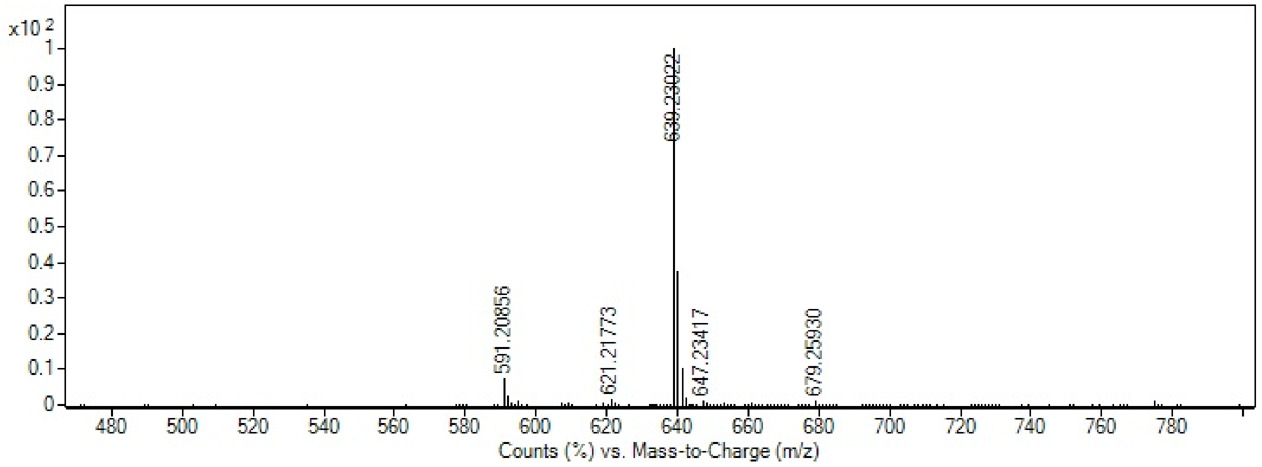

Figure S21. HR-ESI(–)-MS spectrum of geministatin A ozonlysis product (**7**)

NMR Spectra

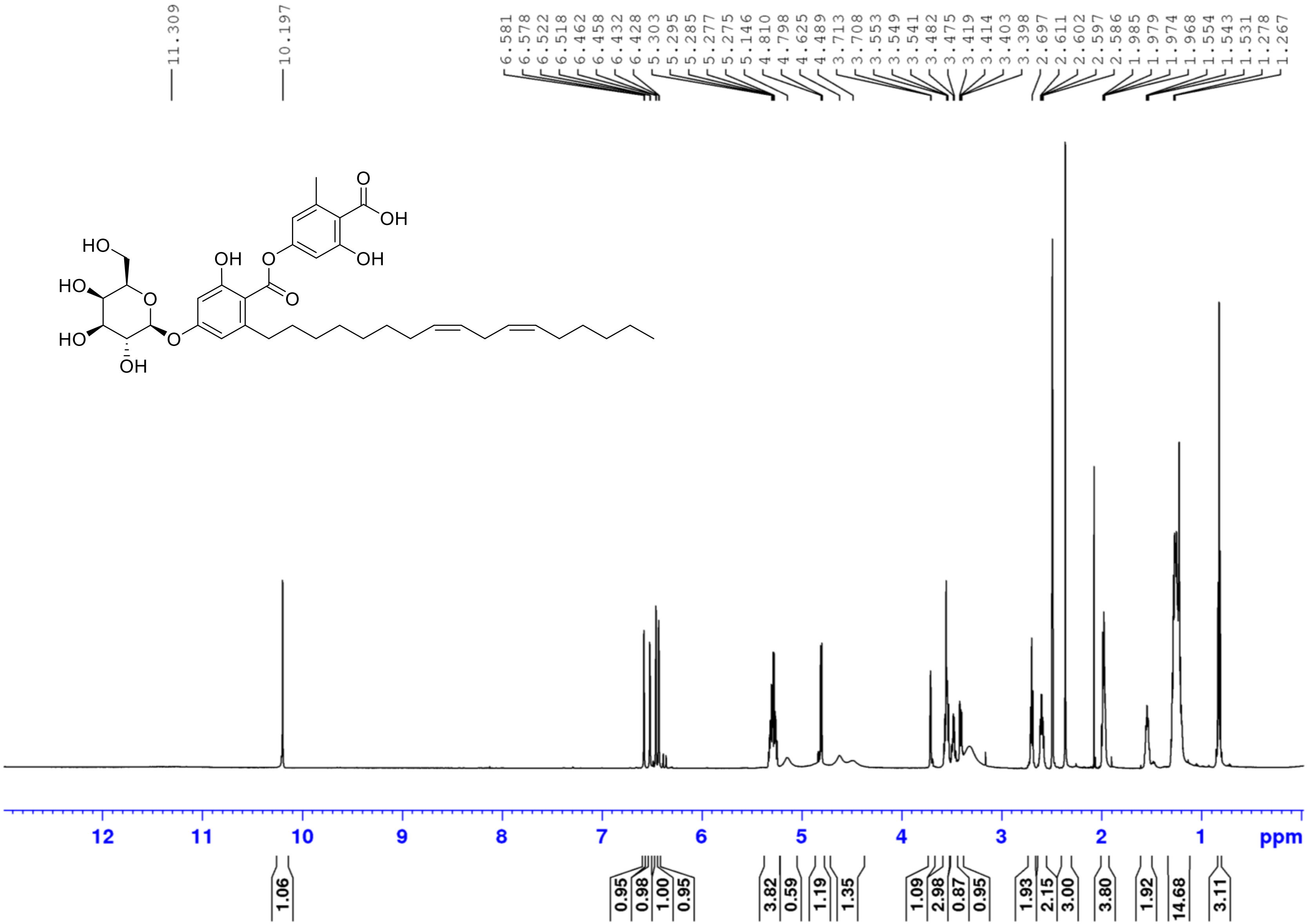

Figure S22. ^{1}H NMR spectrum (600 MHz) of geministatin A (**1**) in DMSO-d_6

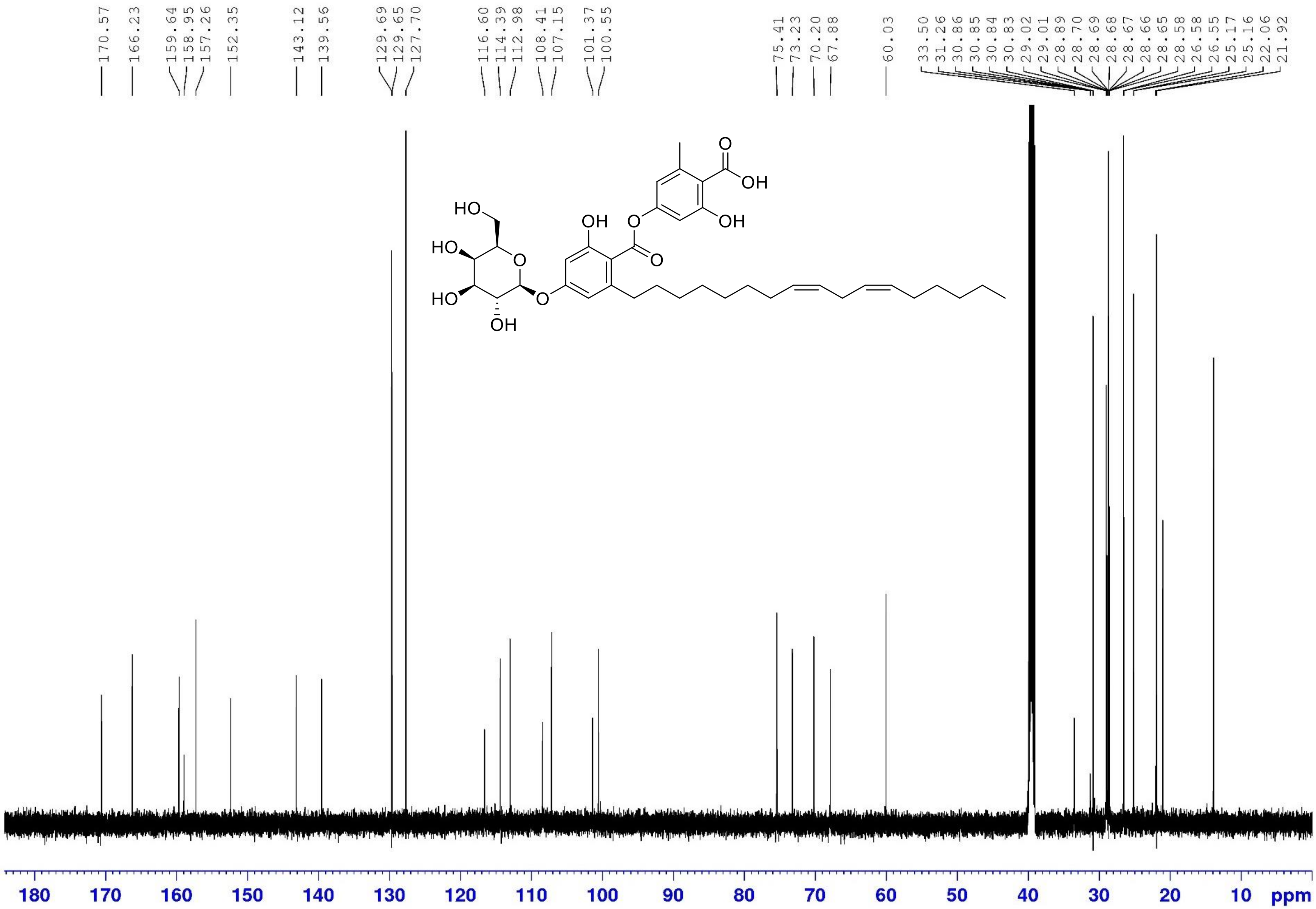

Figure S23. ^{13}C NMR spectrum (150 MHz) of geministatin A (**1**) in DMSO-d_6

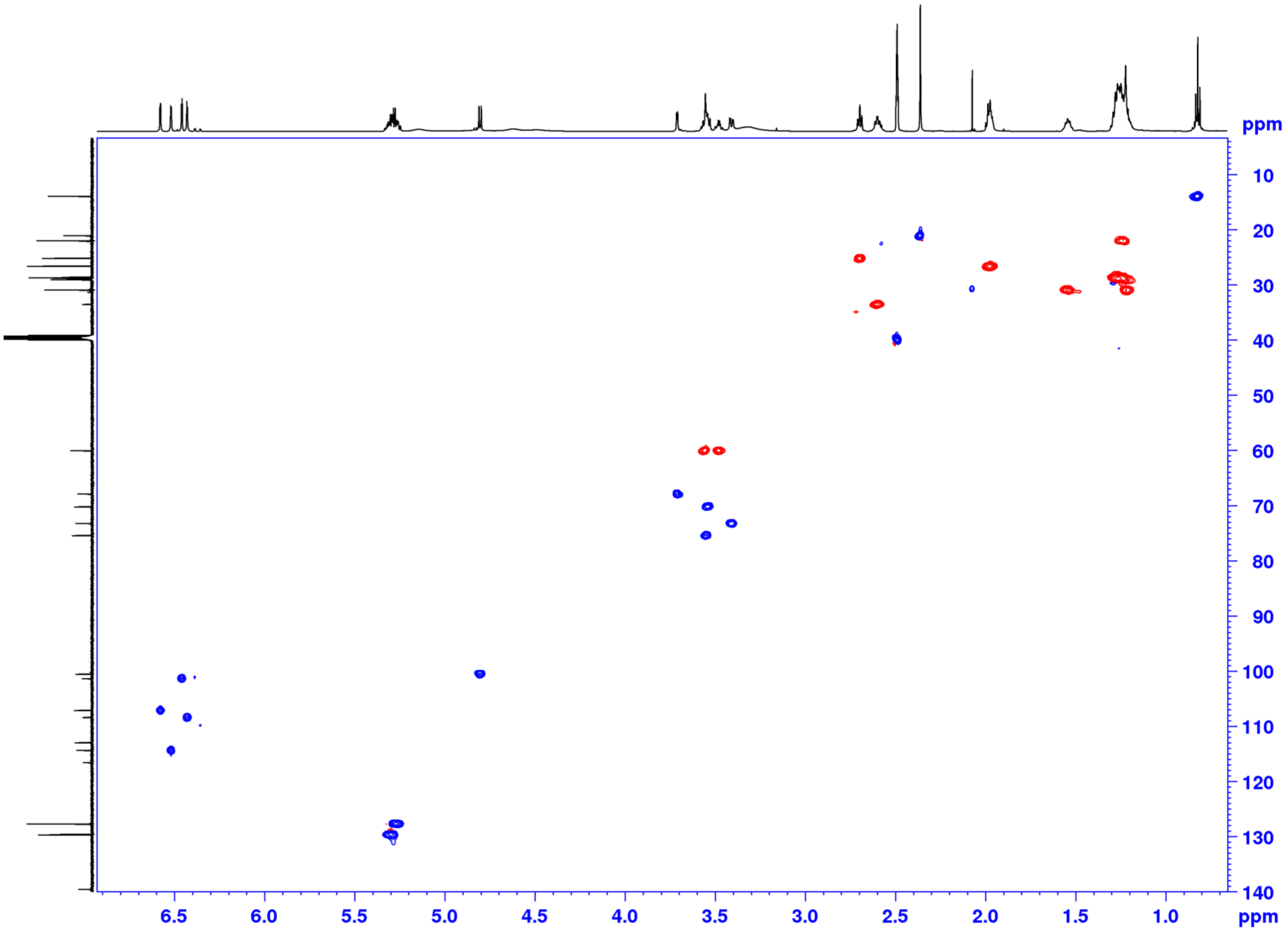

Figure S24. HSQC NMR spectrum (600 MHz) of geministatin A (**1**) in DMSO-d_6

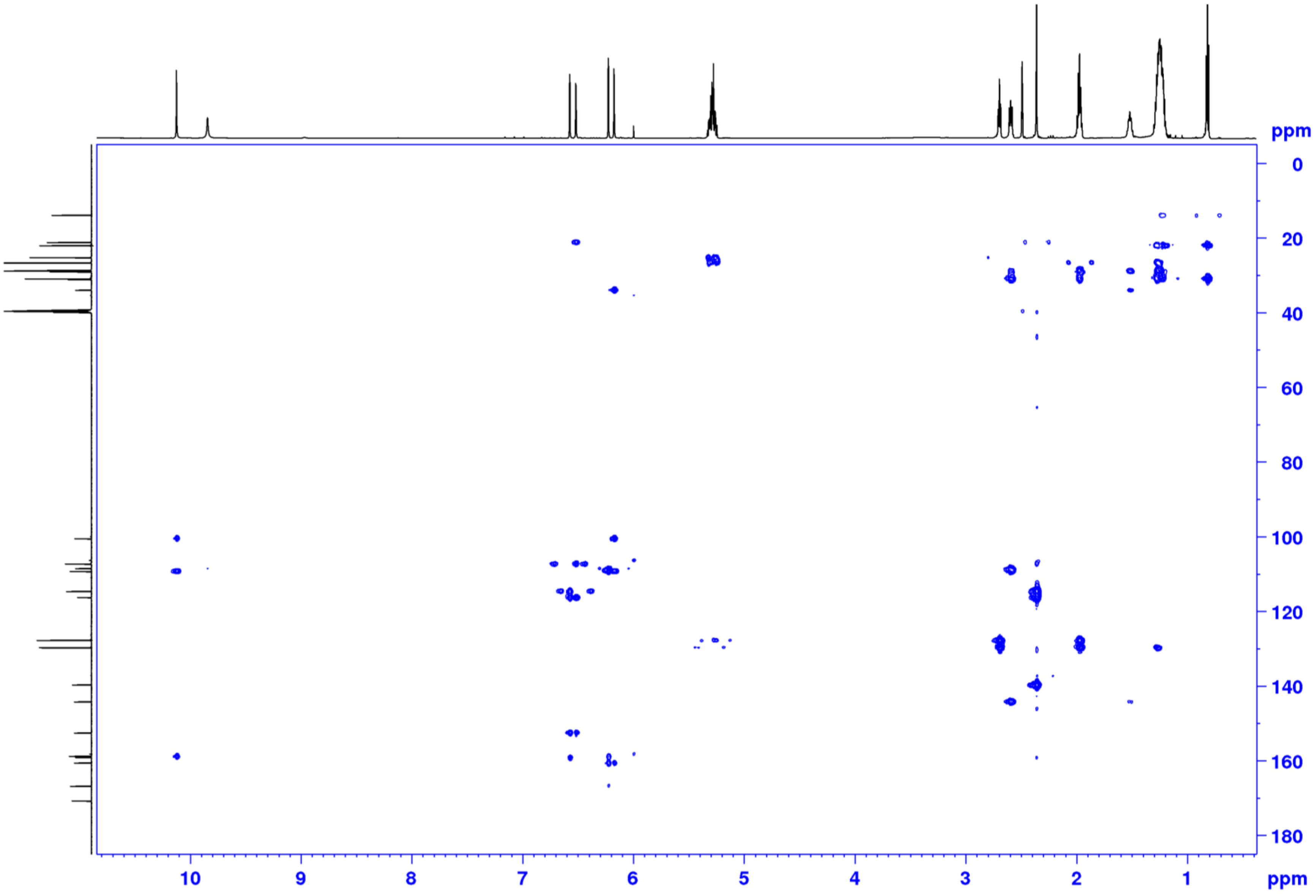

Figure S25. HMBC NMR spectrum (600 MHz) of geministatin A (**1**) in DMSO-d_6

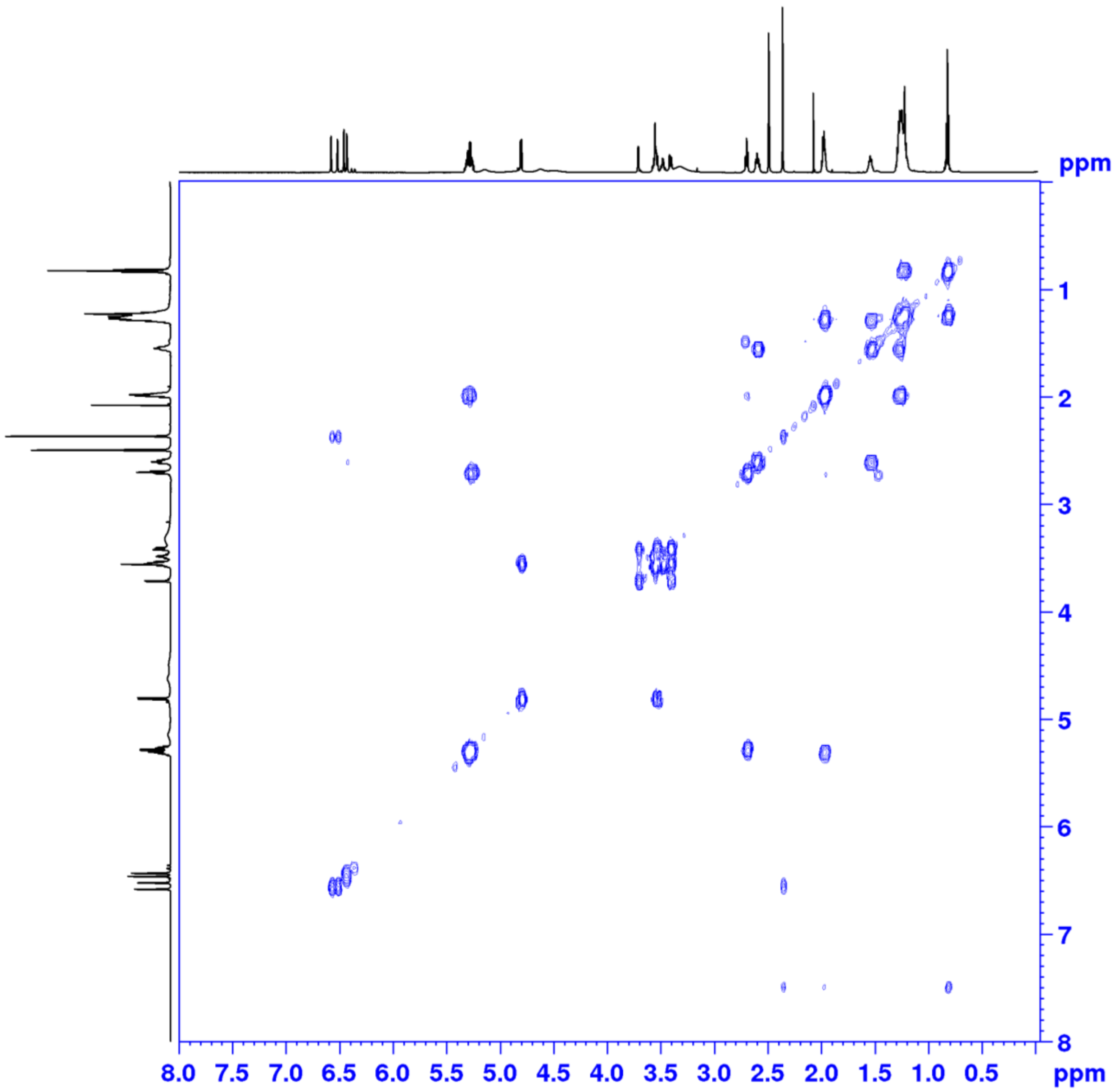

Figure S26. COSY NMR spectrum (600 MHz) of geministatin A (**1**) in DMSO-d_6

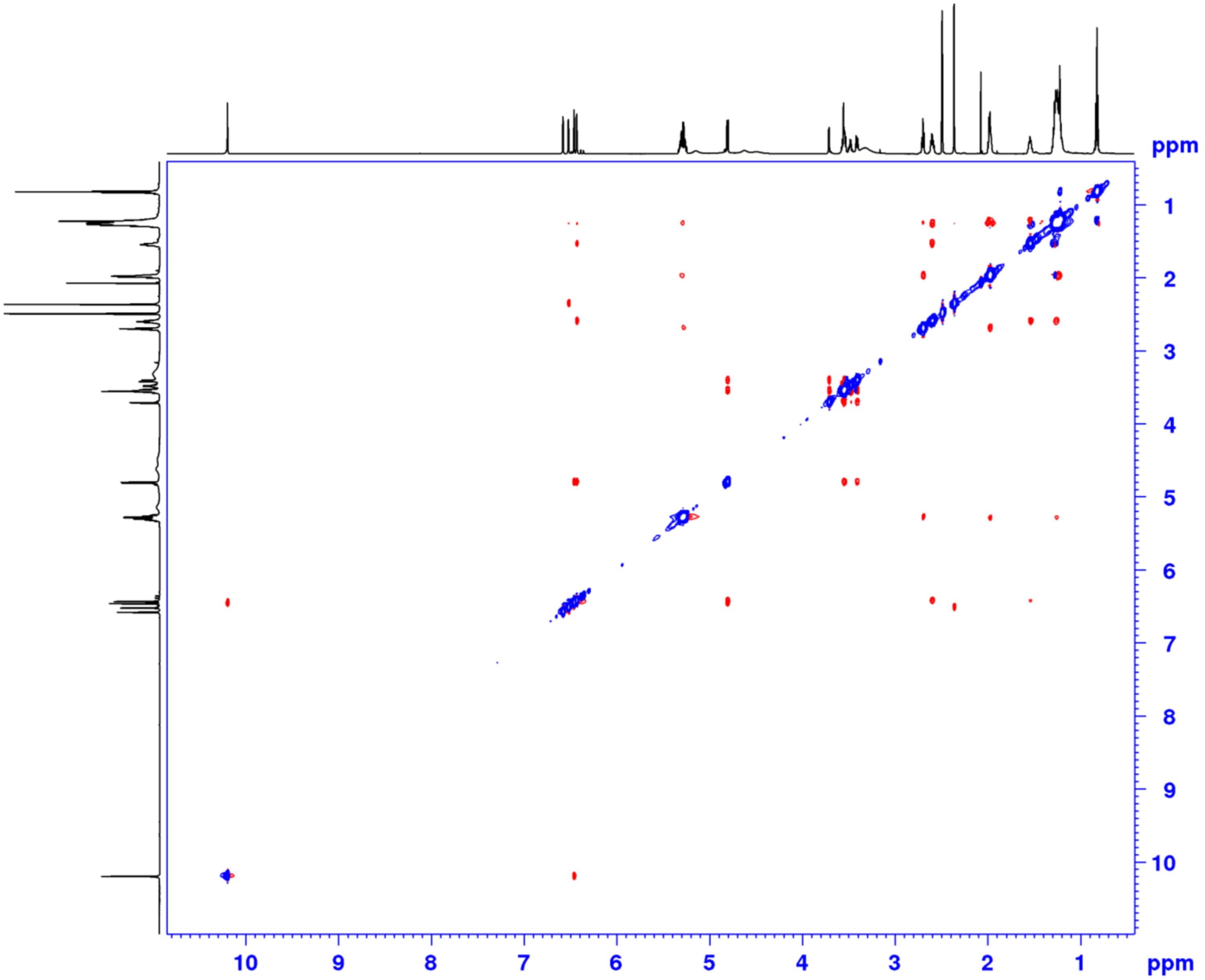

Figure S27. ROESY NMR spectrum (600 MHz) of geministatin A (**1**) in DMSO-d_6

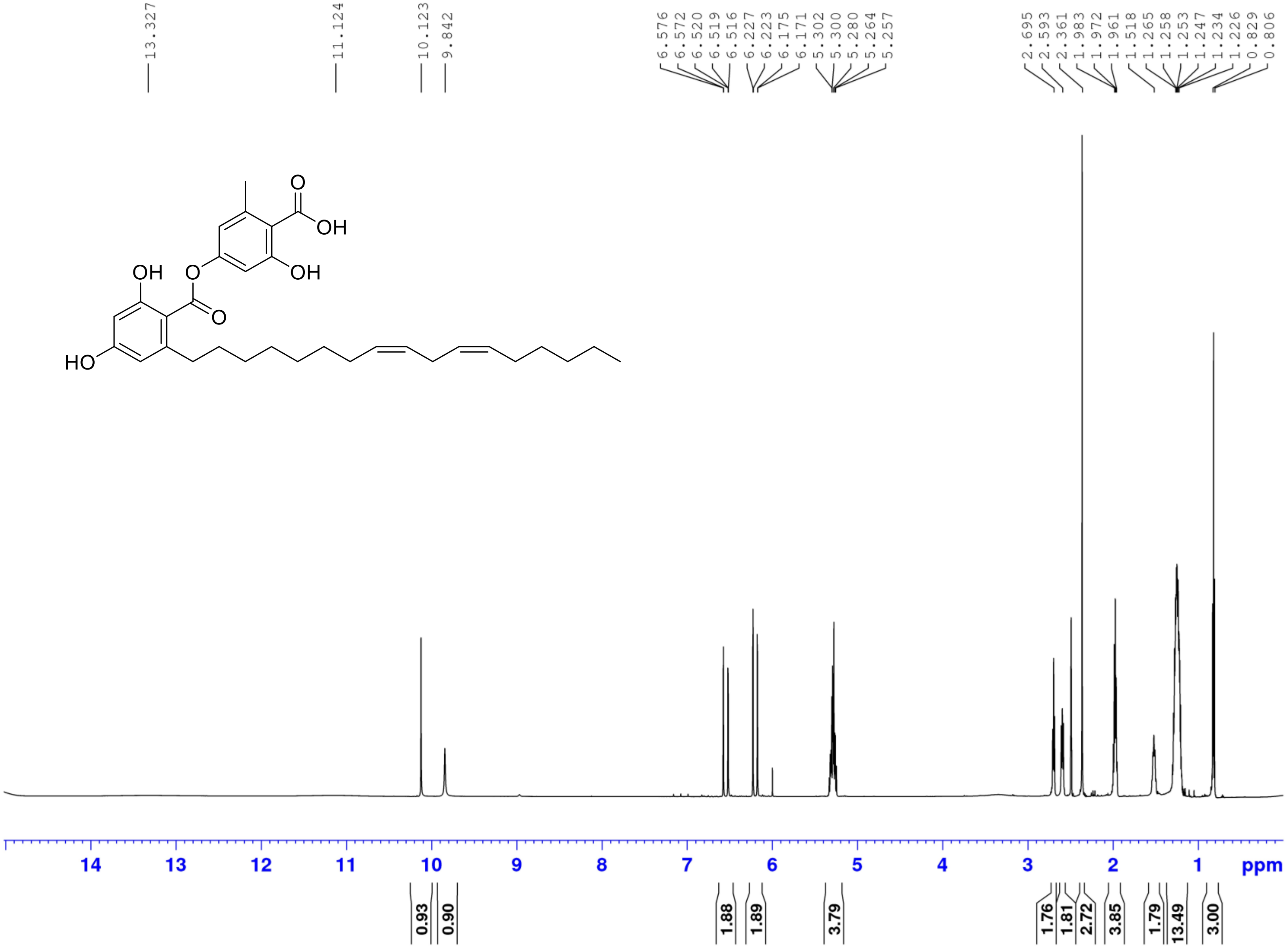

Figure S28. ^{1}H NMR spectrum (600 MHz) of geministatin B (**2**) in DMSO-d_6

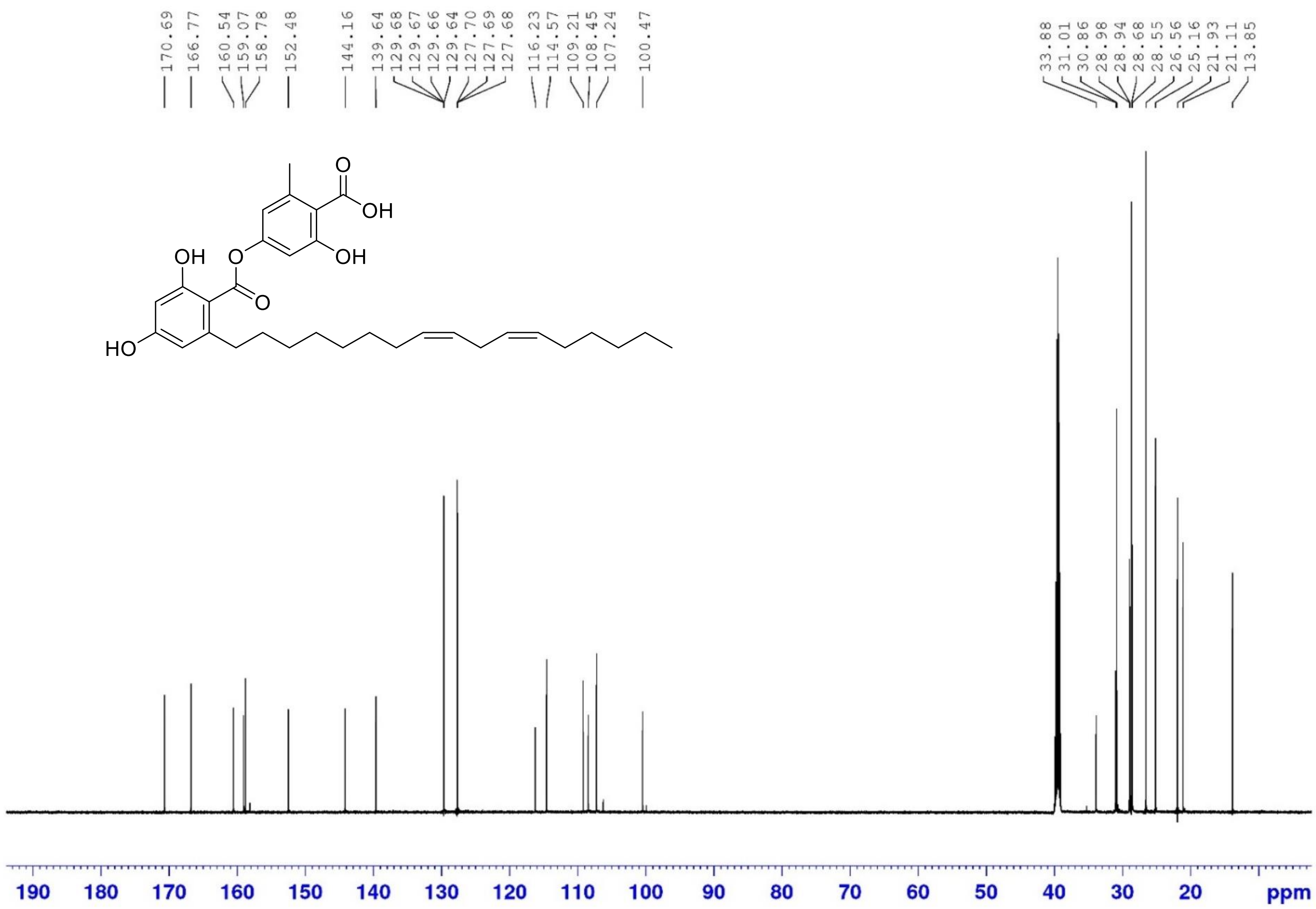

Figure S29. ^{13}C NMR spectrum (150 MHz) of geministatin B (**2**) in DMSO-d_6

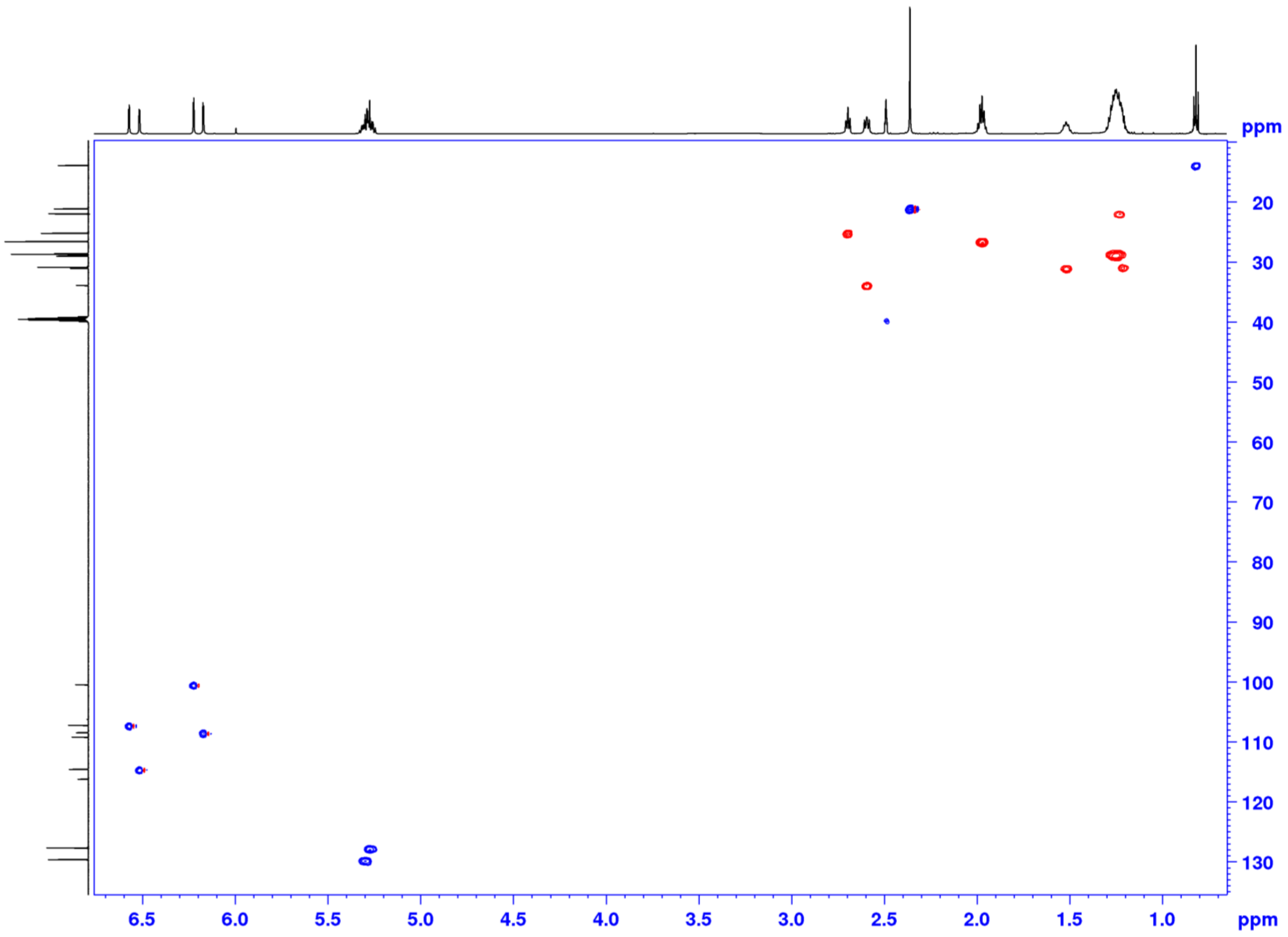

Figure S30. HSQC NMR spectrum (600 MHz) of geministatin B (**2**) in DMSO-d_6

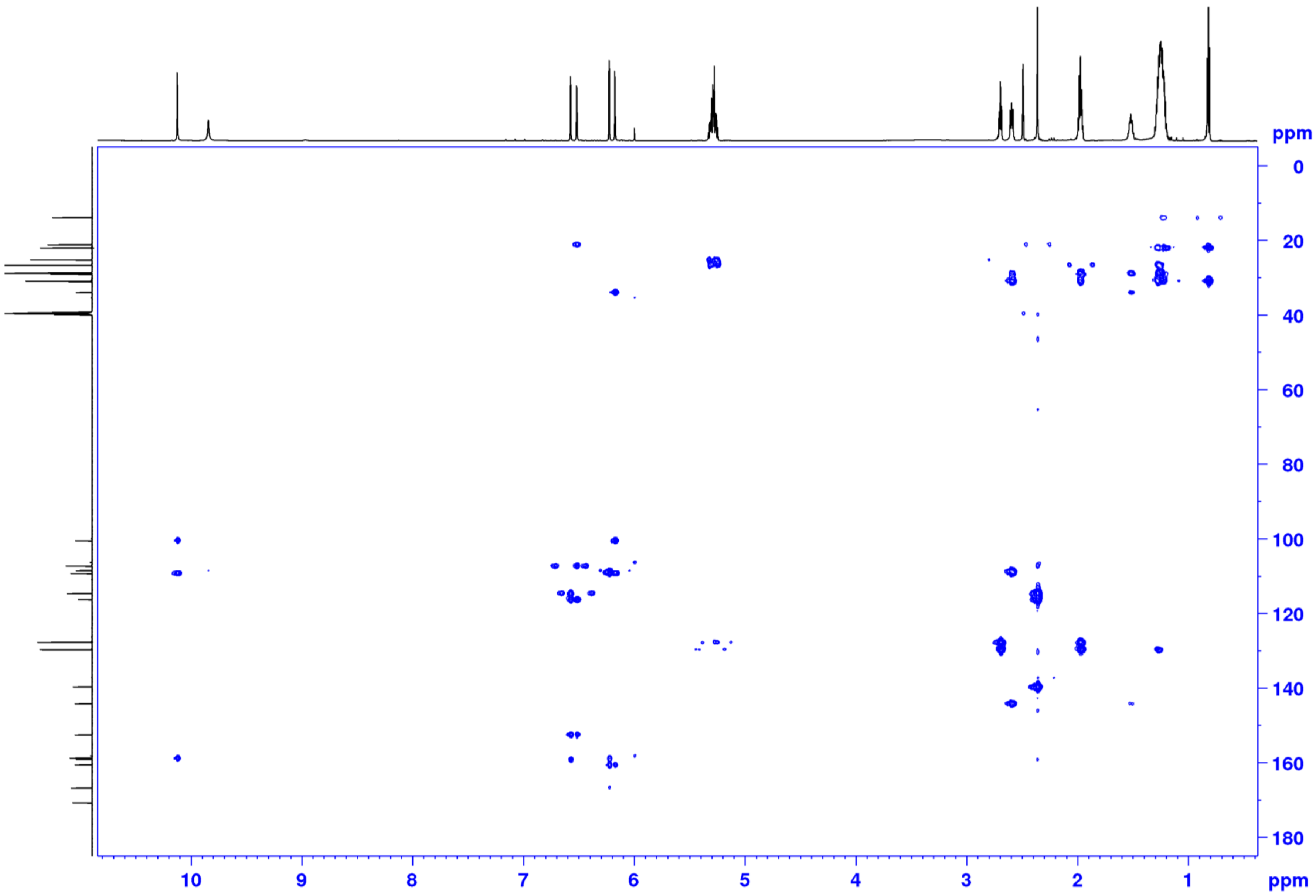

Figure S31. HMBC NMR spectrum (600 MHz) of geministatin B (**2**) in DMSO-d_6

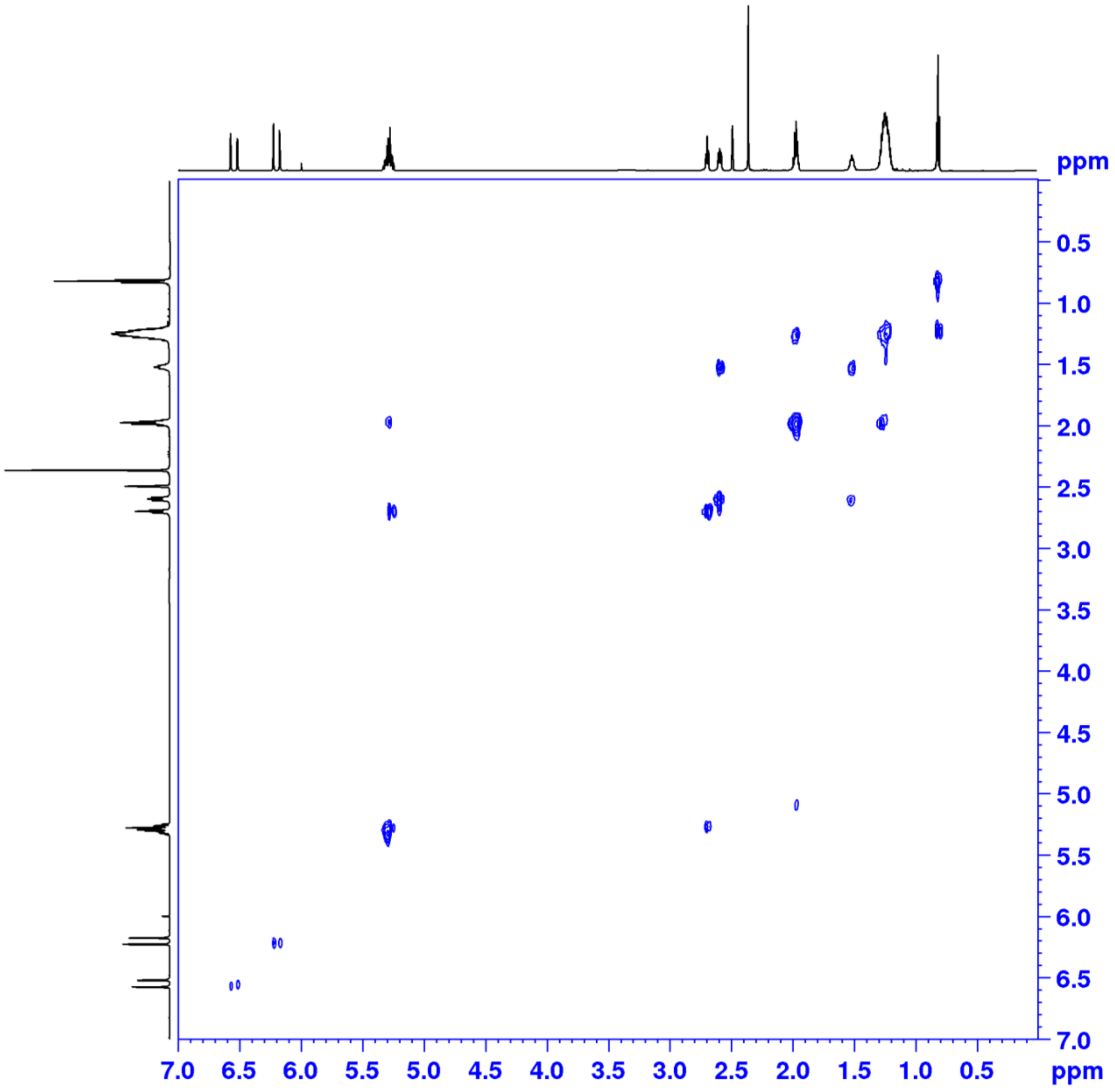

Figure S32. COSY NMR spectrum (600 MHz) of geministatin B (**2**) in DMSO-d_6

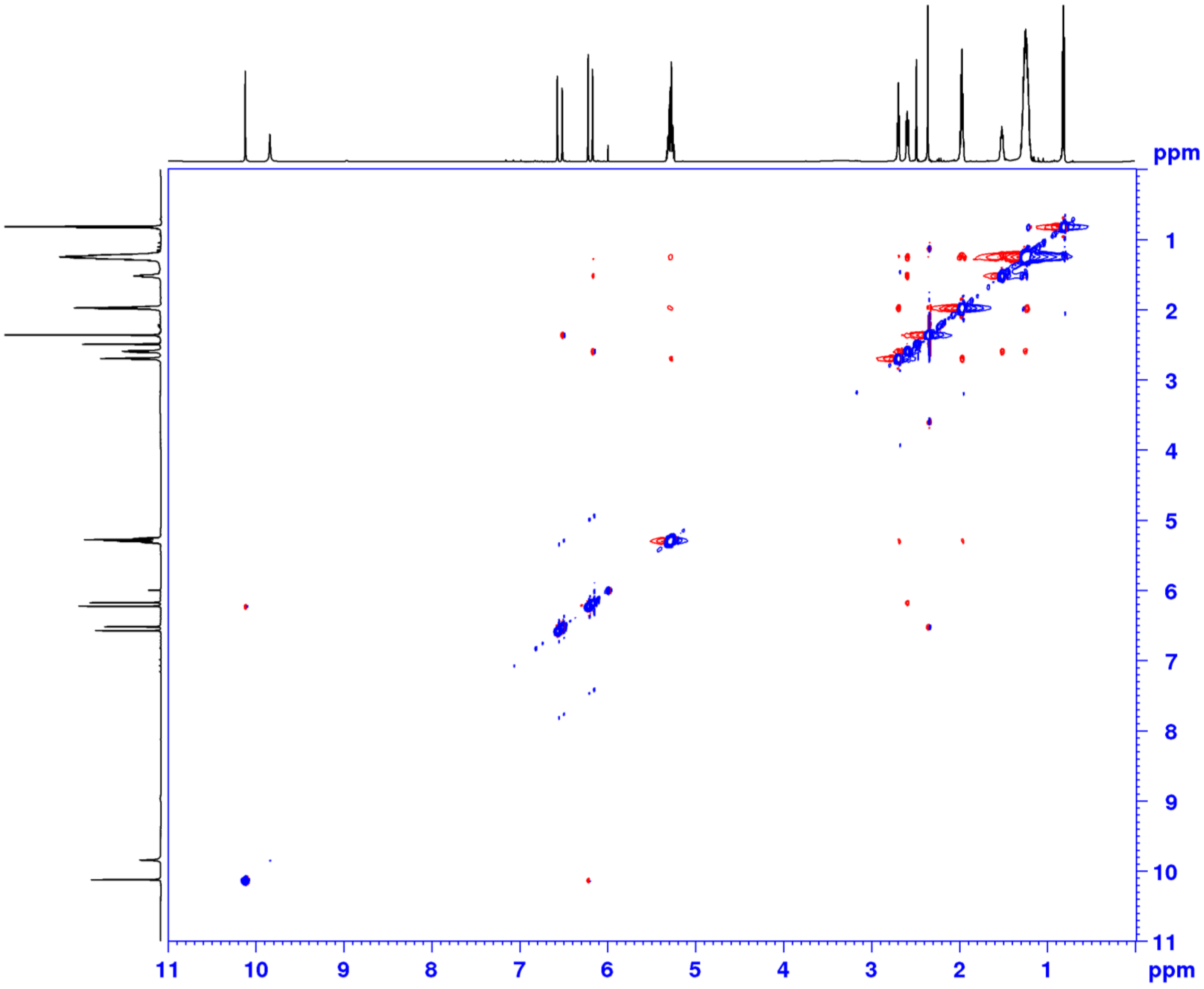

Figure S33. ROESY NMR spectrum (600 MHz) of geministatin B (**2**) in DMSO-d_6

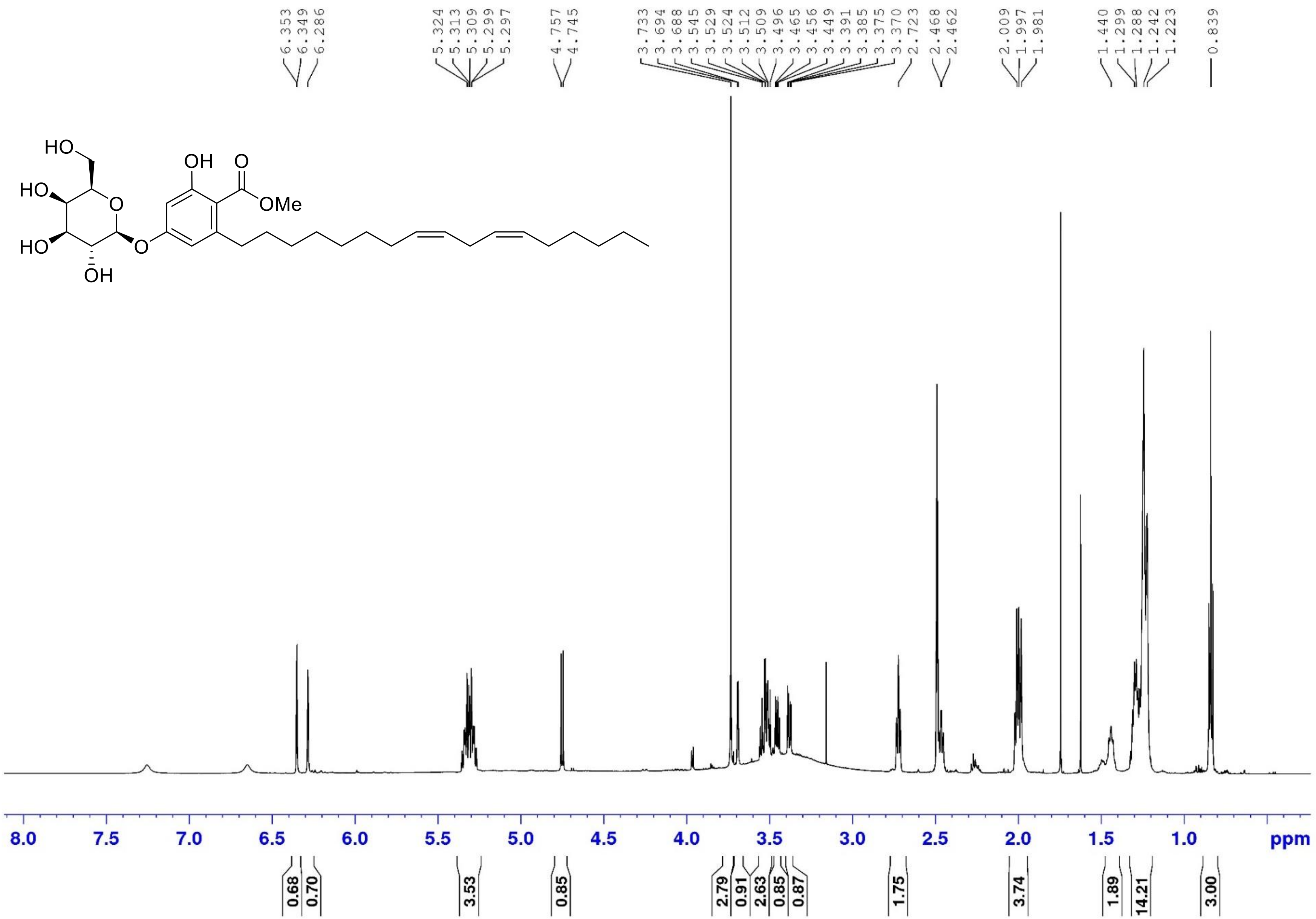

Figure S34. ^{1}H NMR spectrum (600 MHz) of geministatin C (**3**) in DMSO-d_6

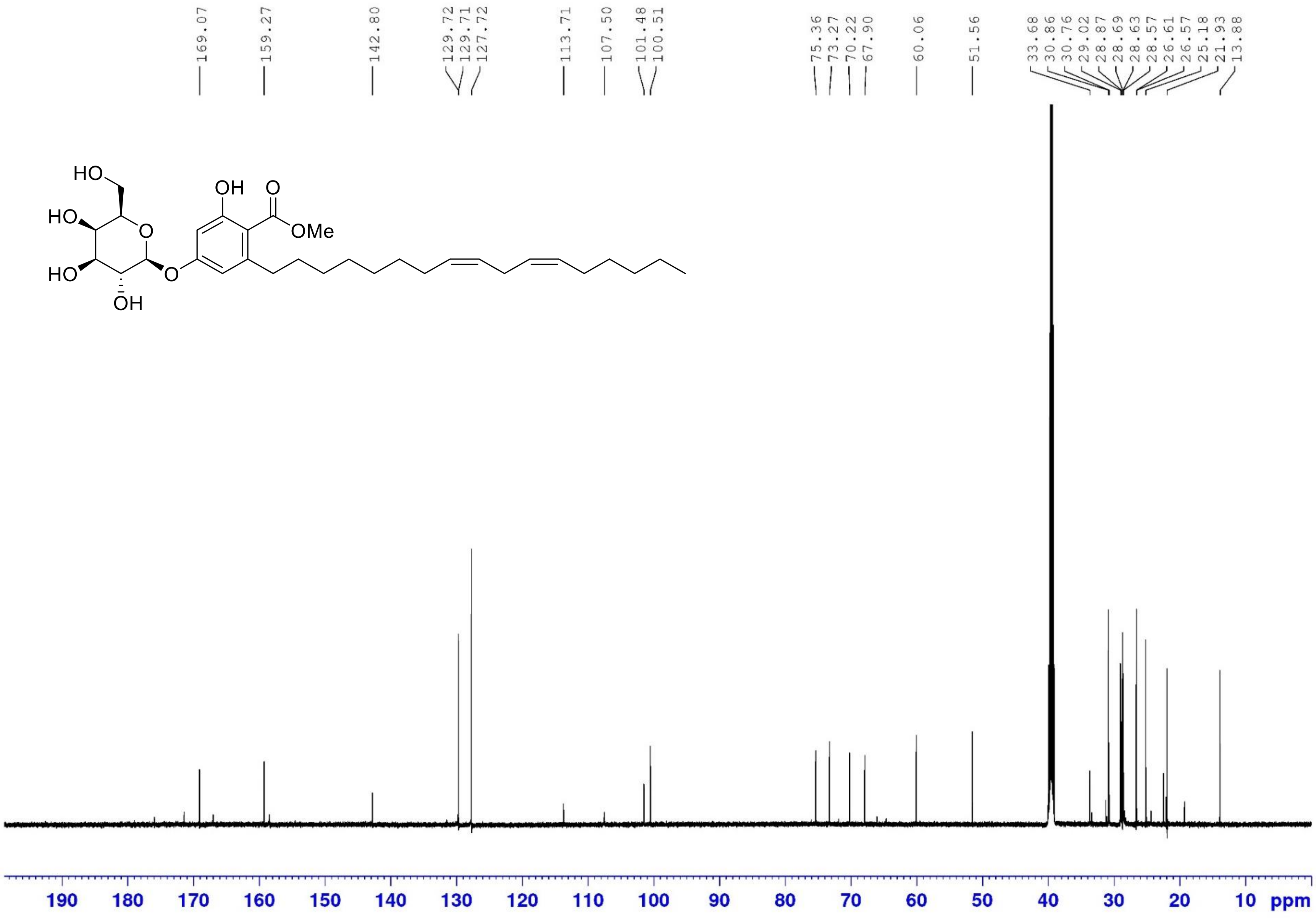

Figure S35. ^{13}C NMR spectrum (150 MHz) of geministatin C (**3**) in DMSO-d_6

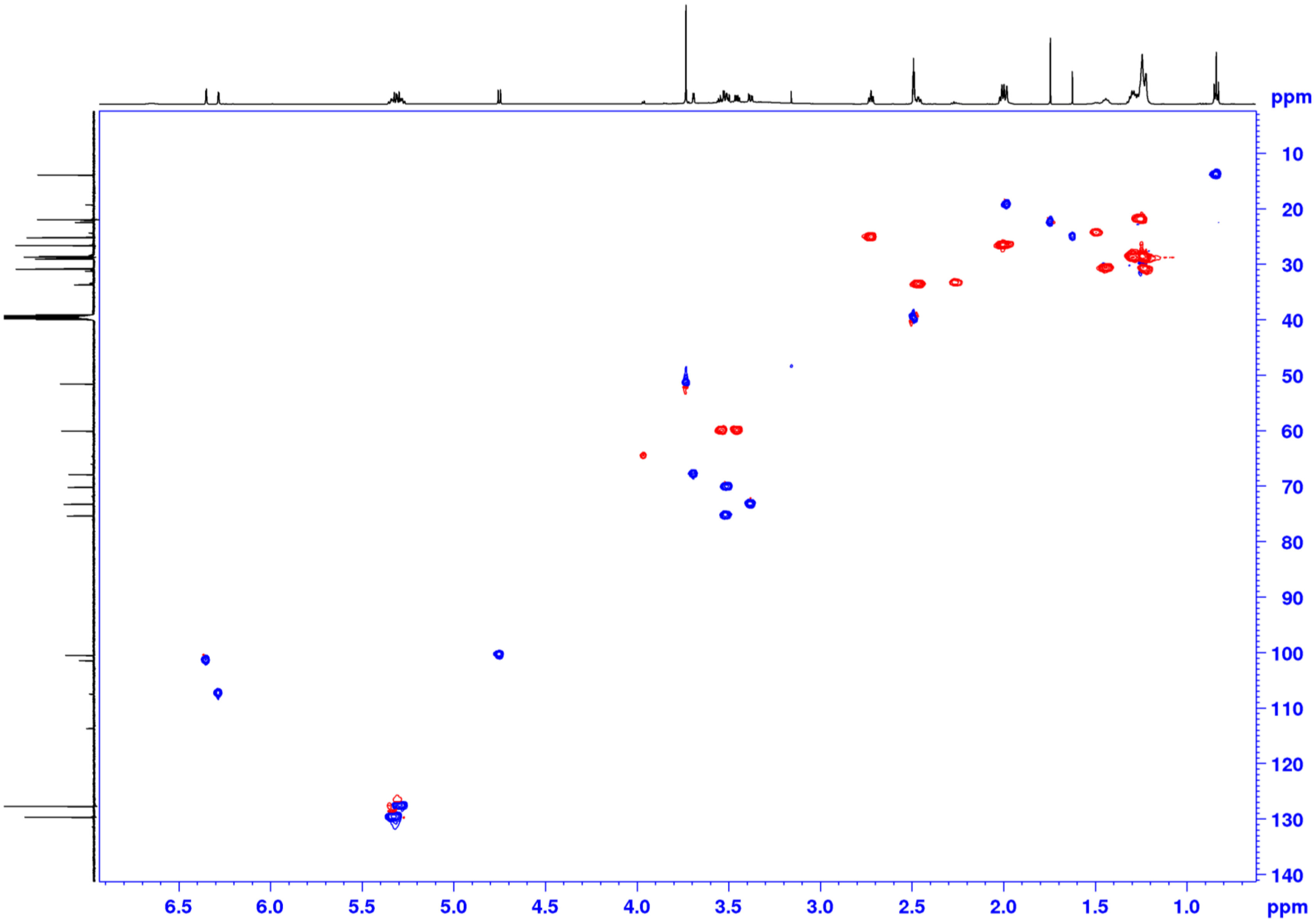

Figure S36. HSQC NMR spectrum (600 MHz) of geministatin C (**3**) in DMSO-d_6

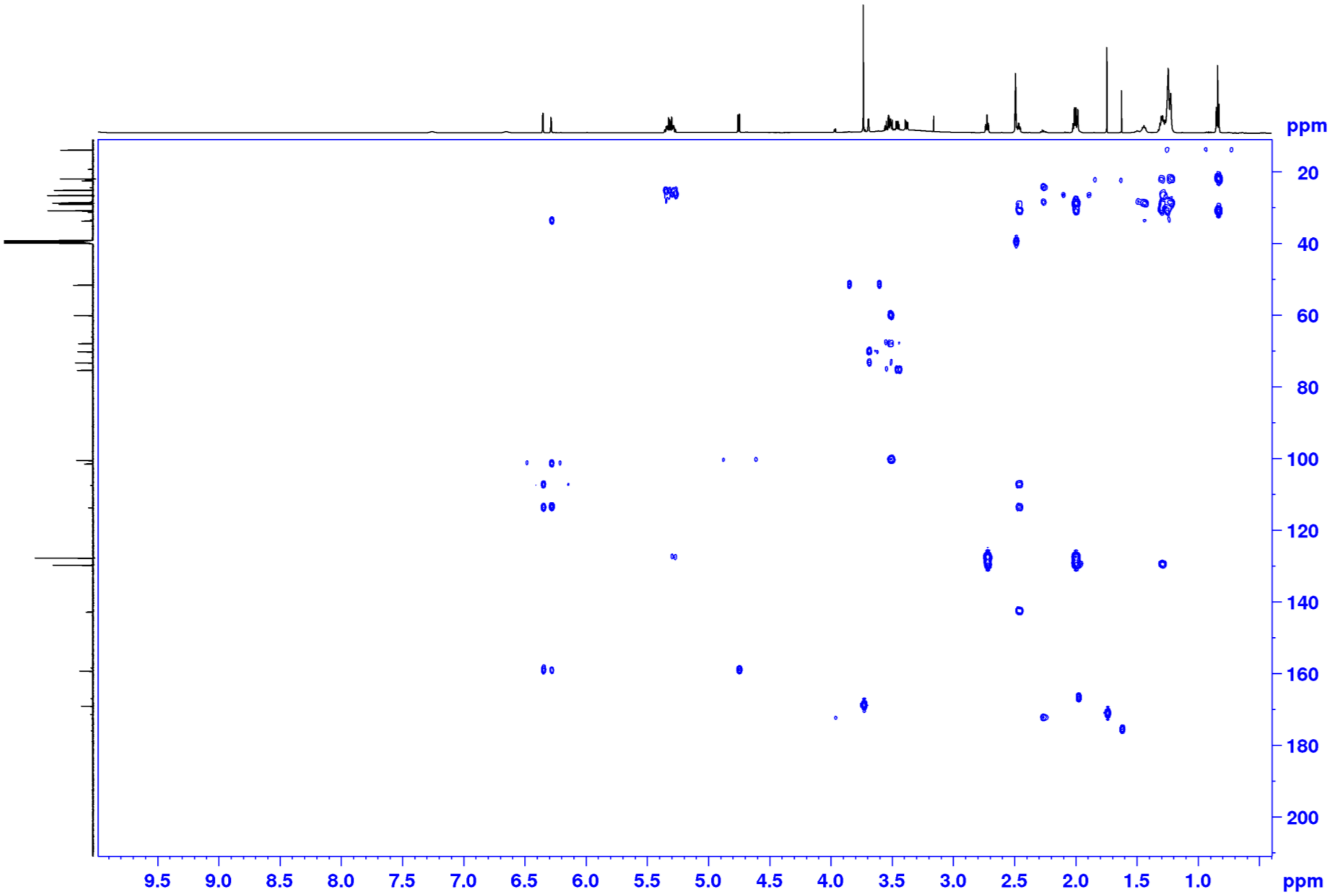

Figure S37. HMBC NMR spectrum (600 MHz) of geministatin C (**3**) in DMSO-d_6

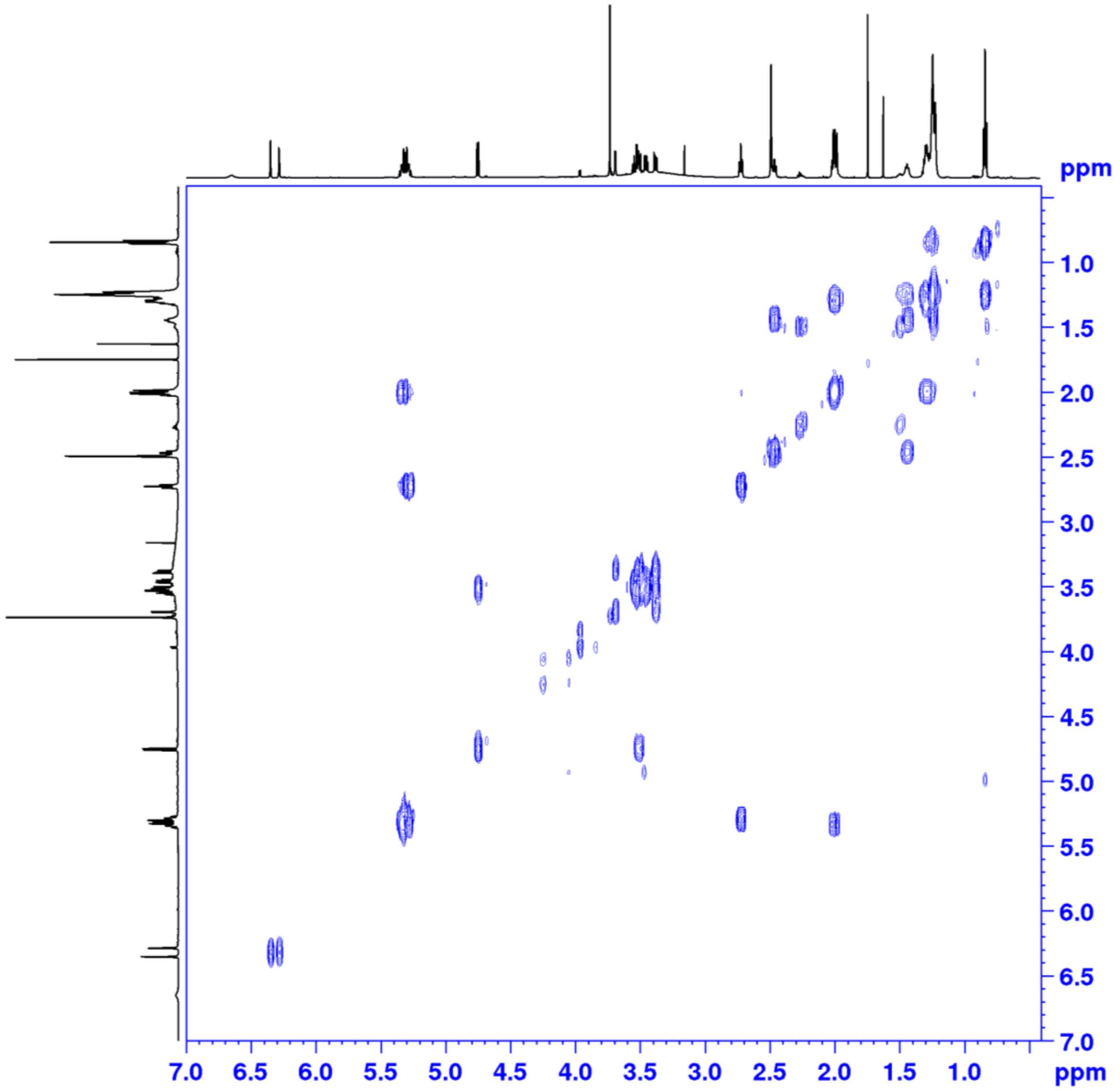

Figure S38. COSY NMR spectrum (600 MHz) of geministatin C (**3**) in DMSO-d_6

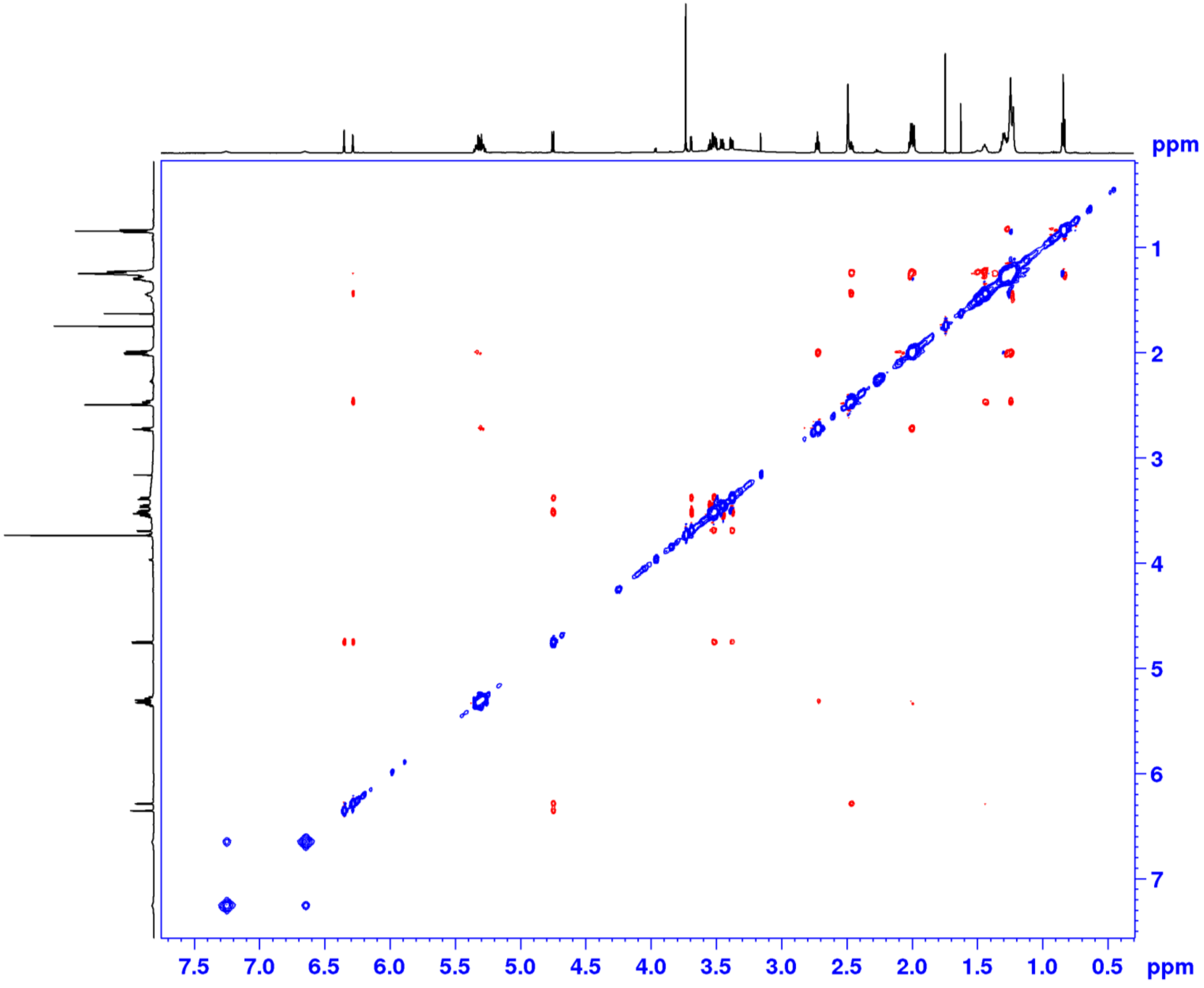

Figure S39. ROESY NMR spectrum (600 MHz) of geministatin C (**3**) in DMSO-d_6

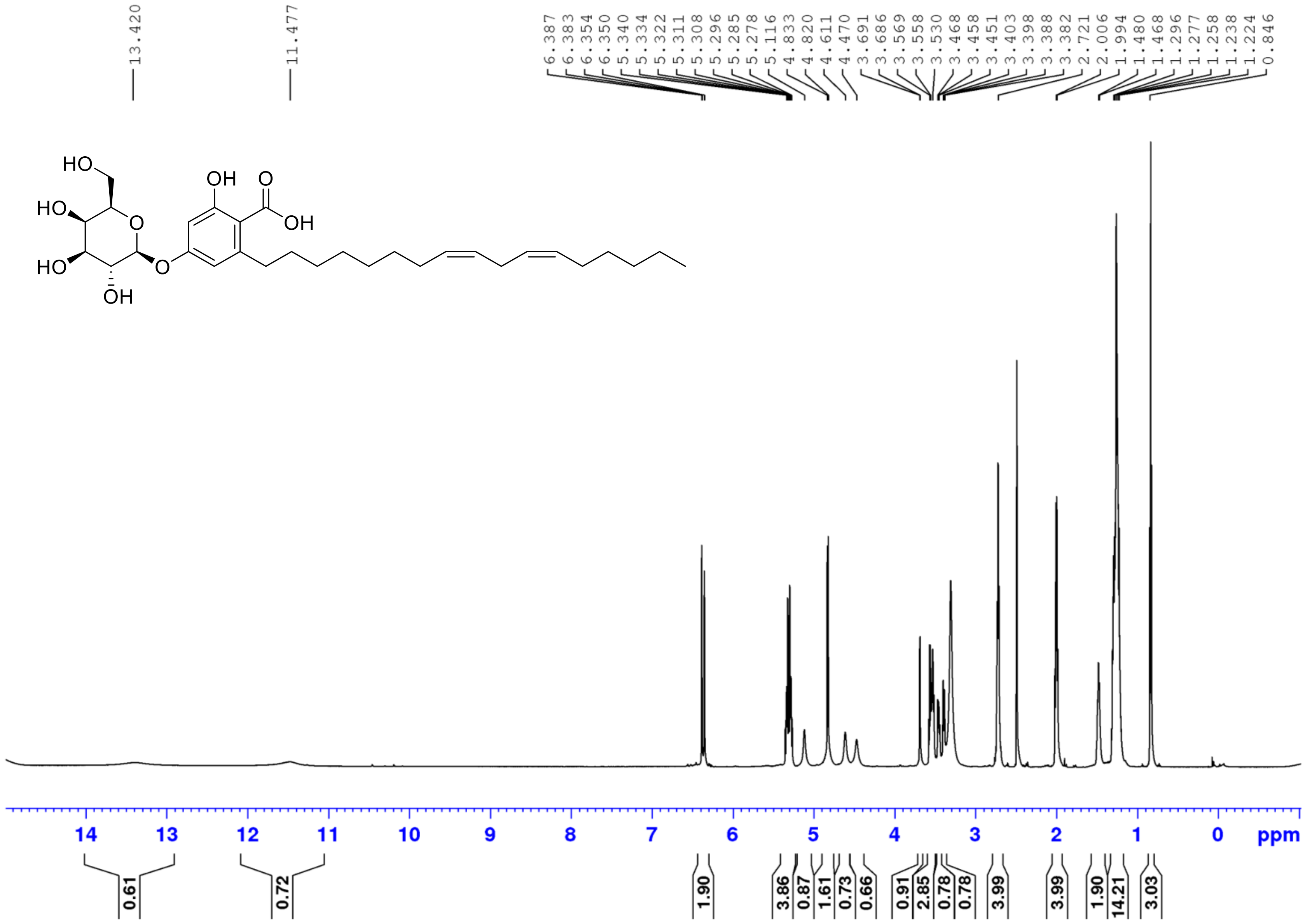

Figure S40. ^{1}H NMR spectrum (600 MHz) of geministatin D (**4**) in DMSO-d_6

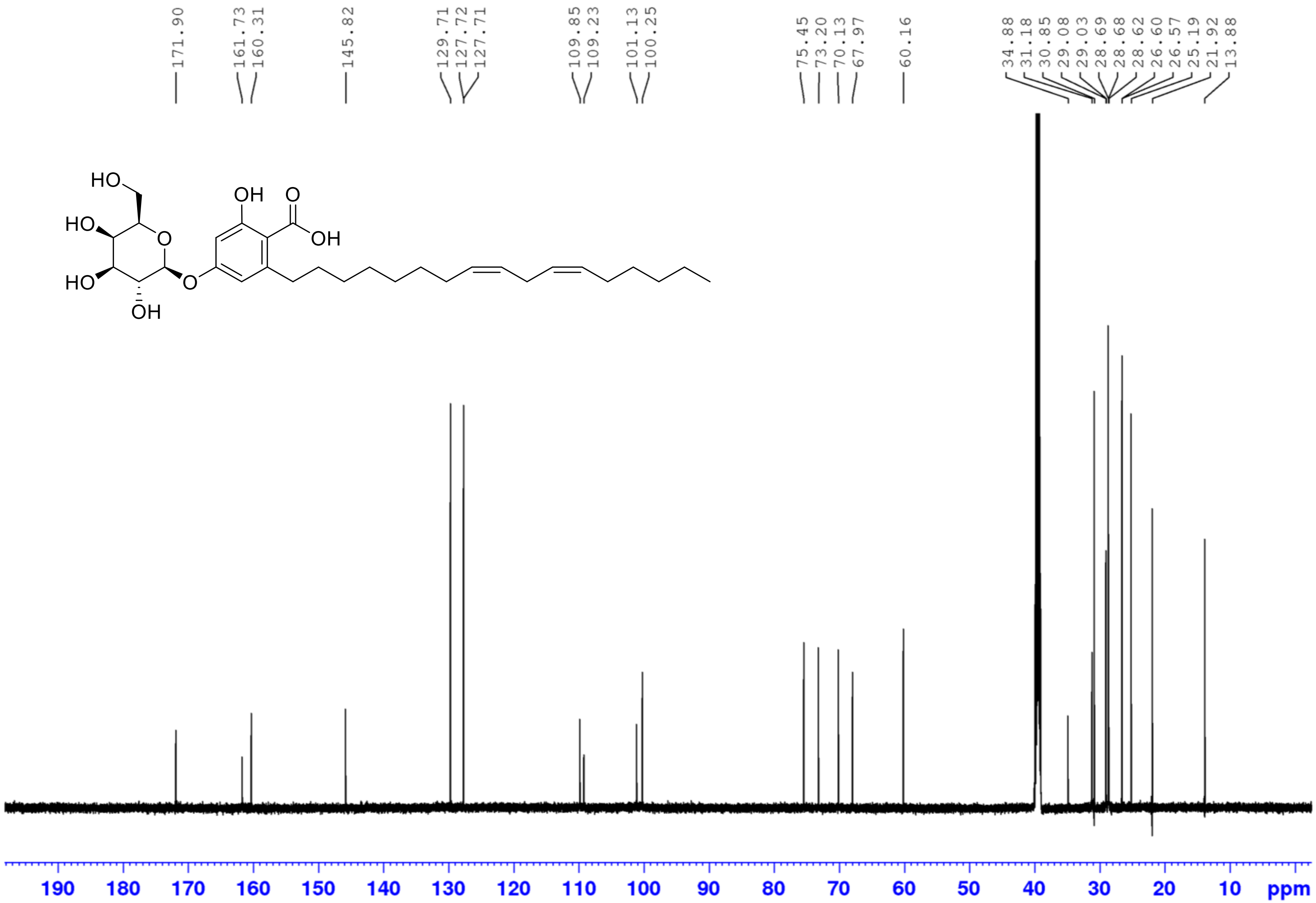

Figure S41. ^{13}C NMR spectrum (150 MHz) of geministatin D (**4**) in DMSO-d_6

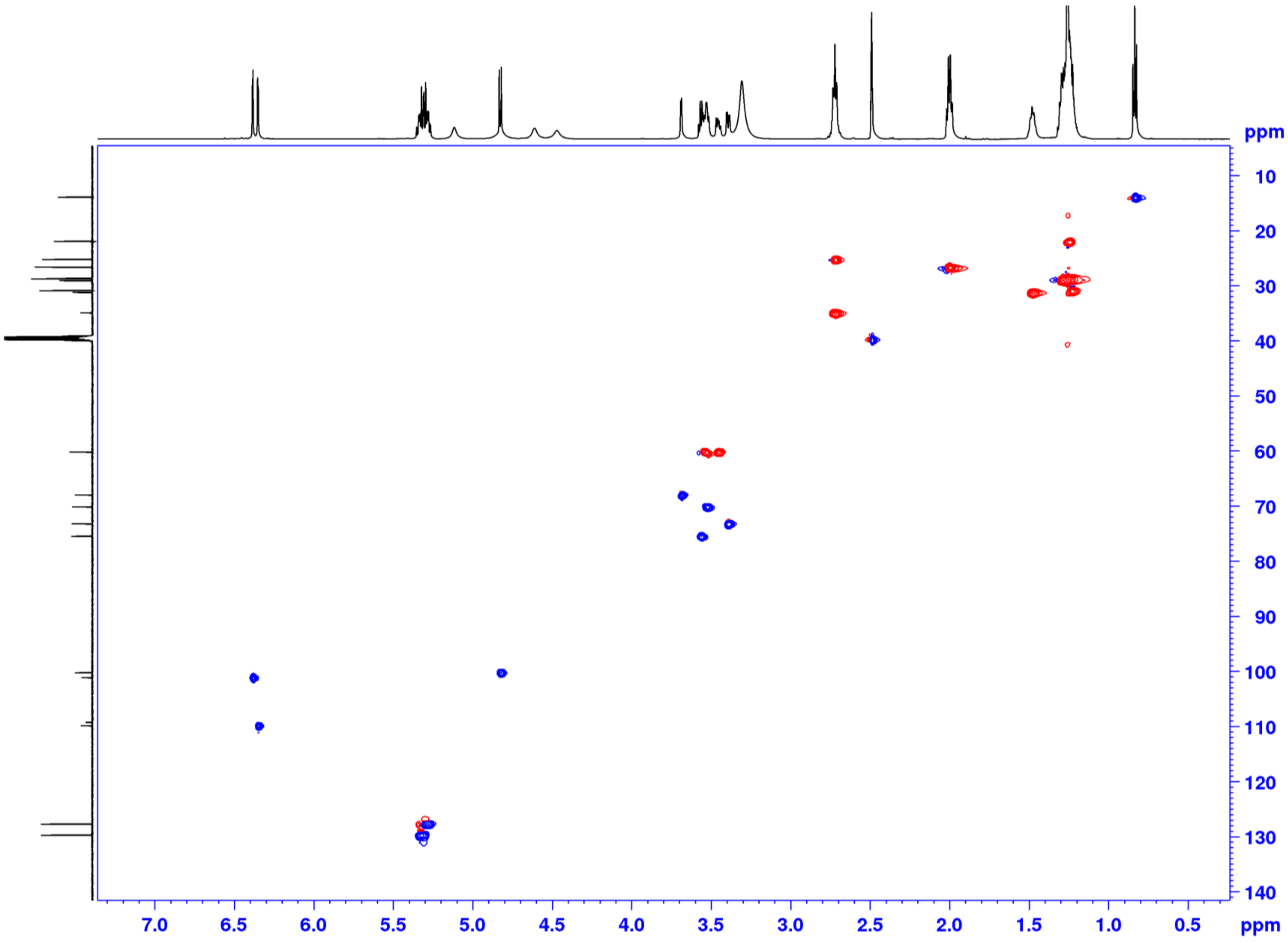

Figure S42. HSQC NMR spectrum (600 MHz) of geministatin D (**4**) in DMSO-d_6

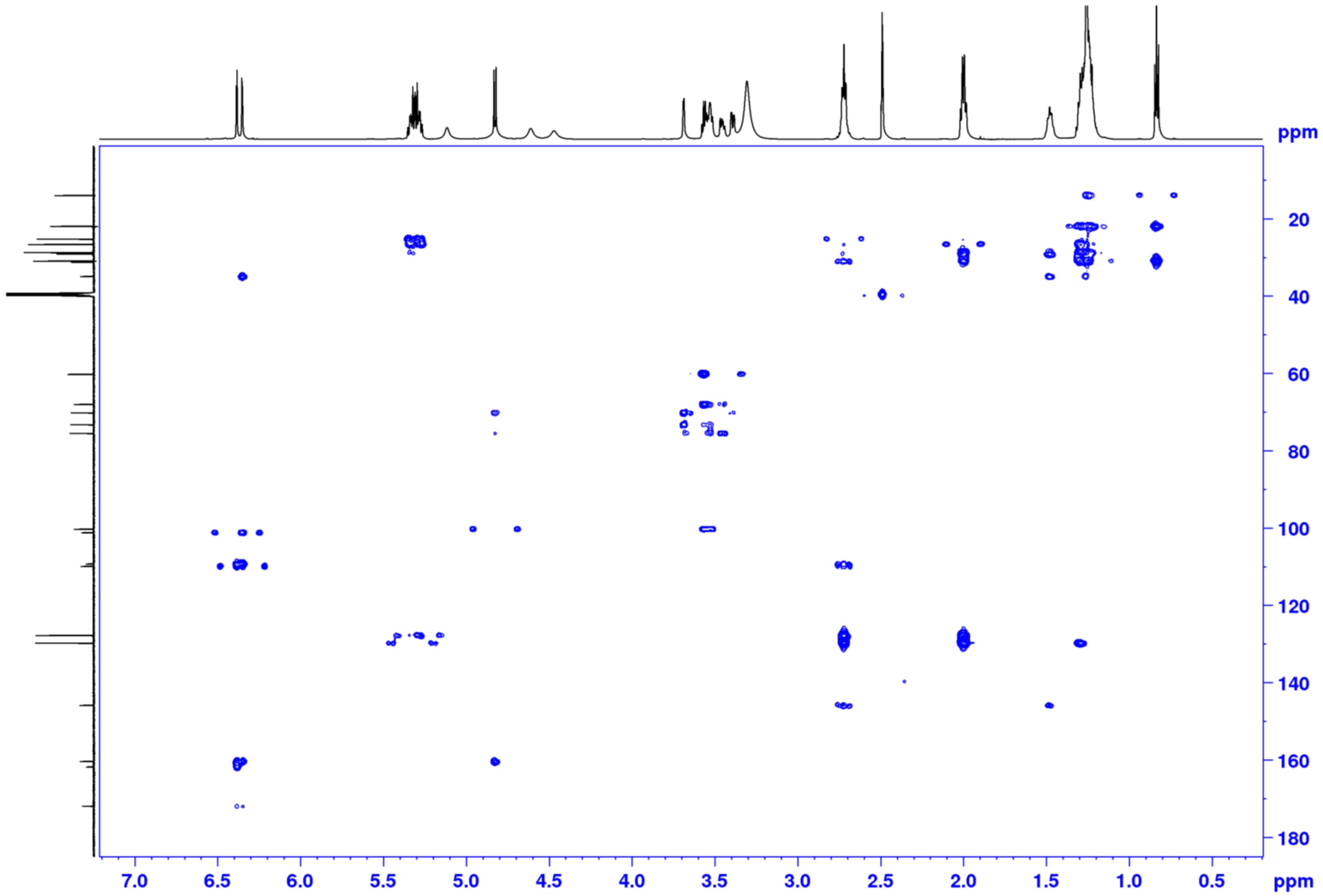

Figure S43. HMBC NMR spectrum (600 MHz) of geministatin D (**4**) in DMSO-d_6

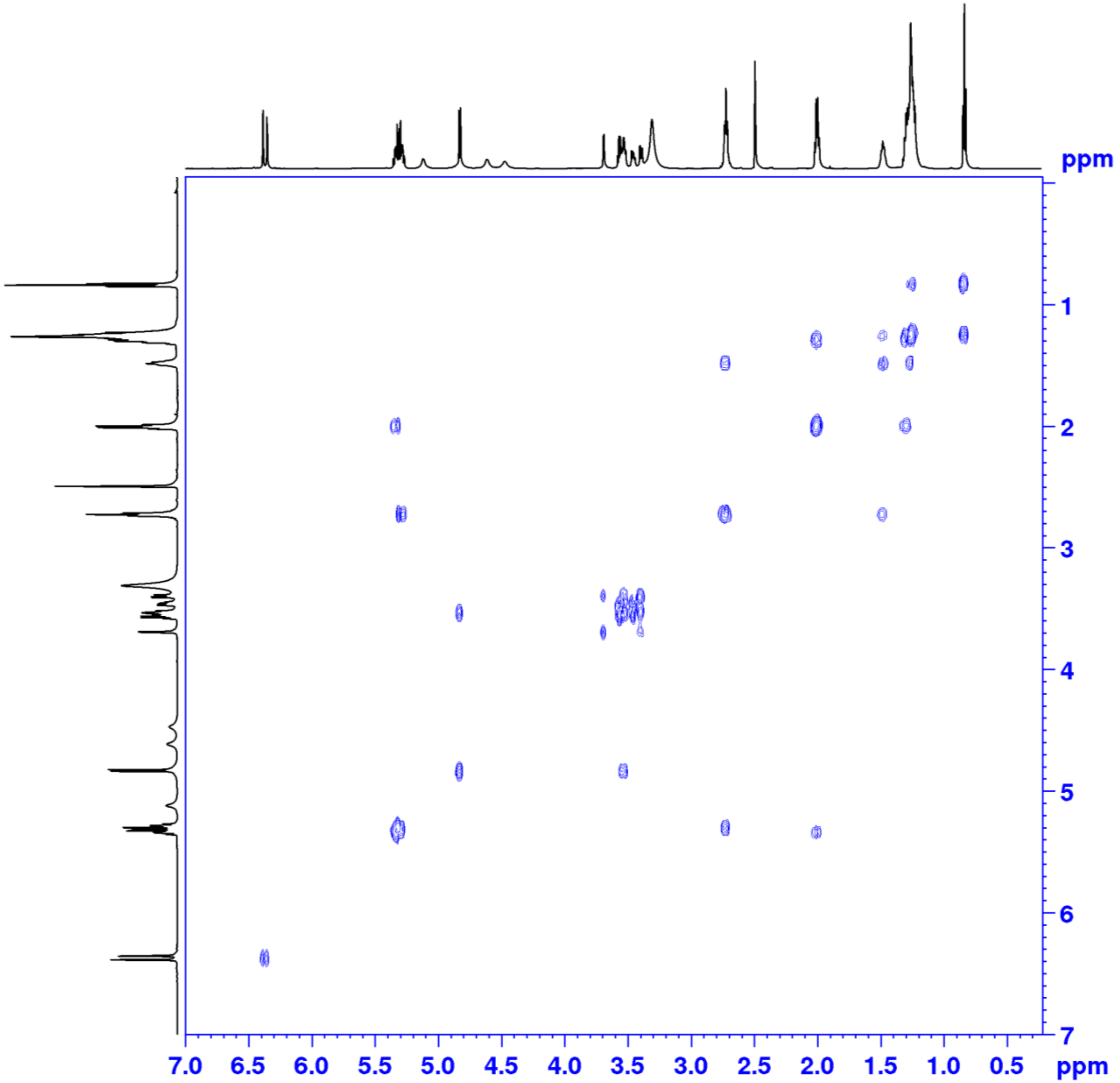

Figure S44. COSY NMR spectrum (600 MHz) of geministatin D (**4**) in DMSO-d_6

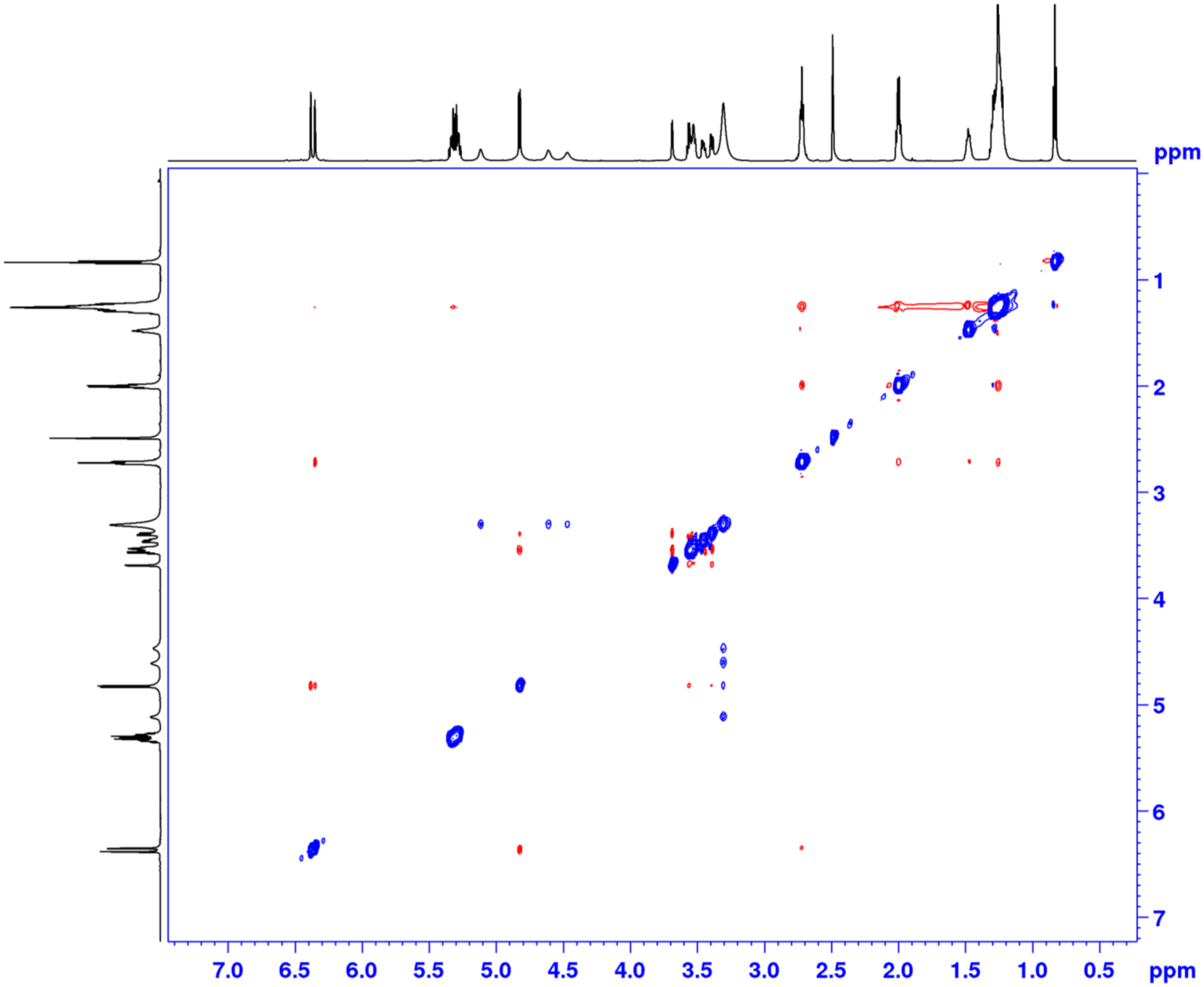

Figure S45. ROESY NMR spectrum (600 MHz) of geministatin D (**4**) in DMSO-d_6

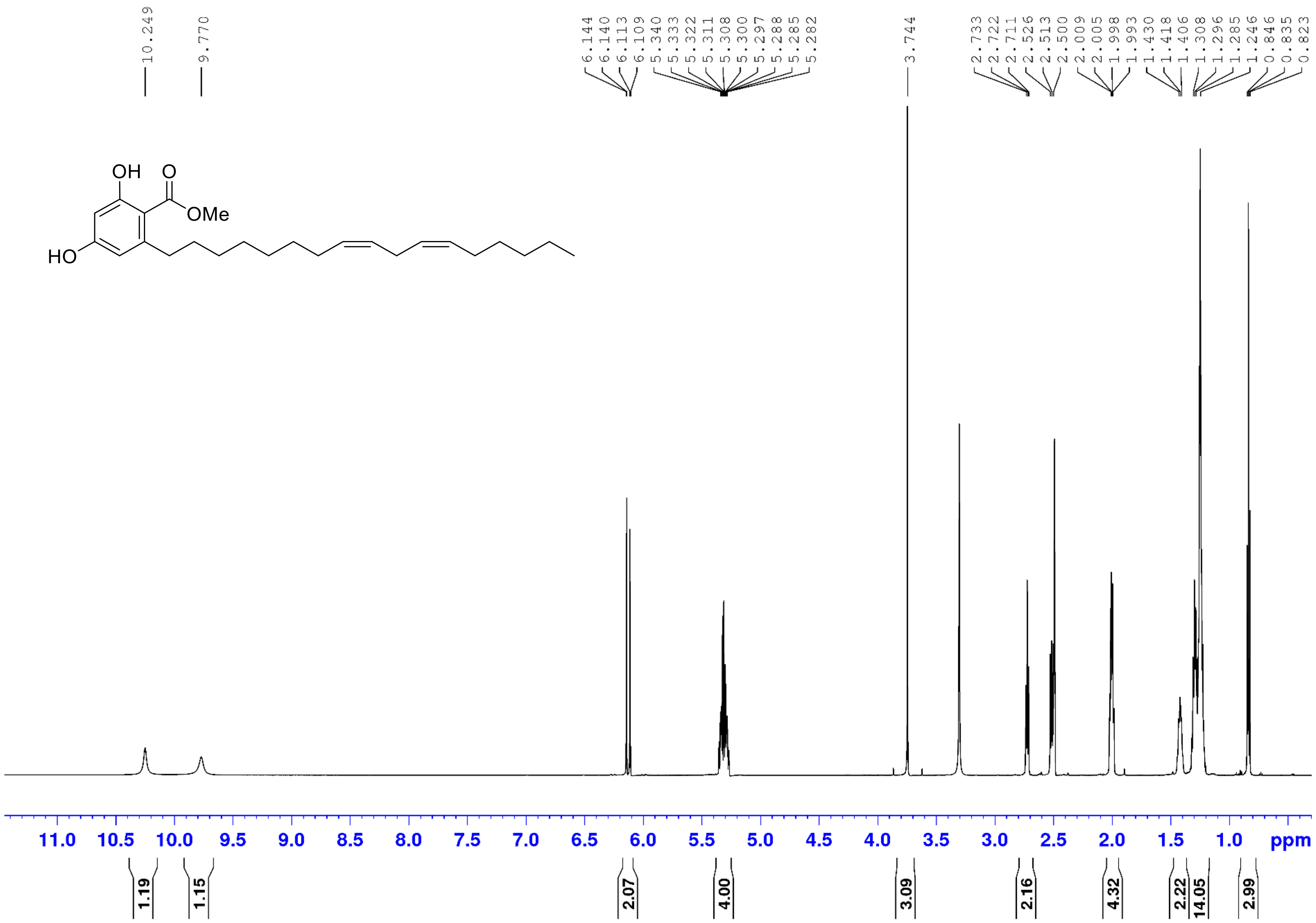

Figure S46. ^{1}H NMR spectrum (600 MHz) of geministatin E (**5**) in DMSO-d_6

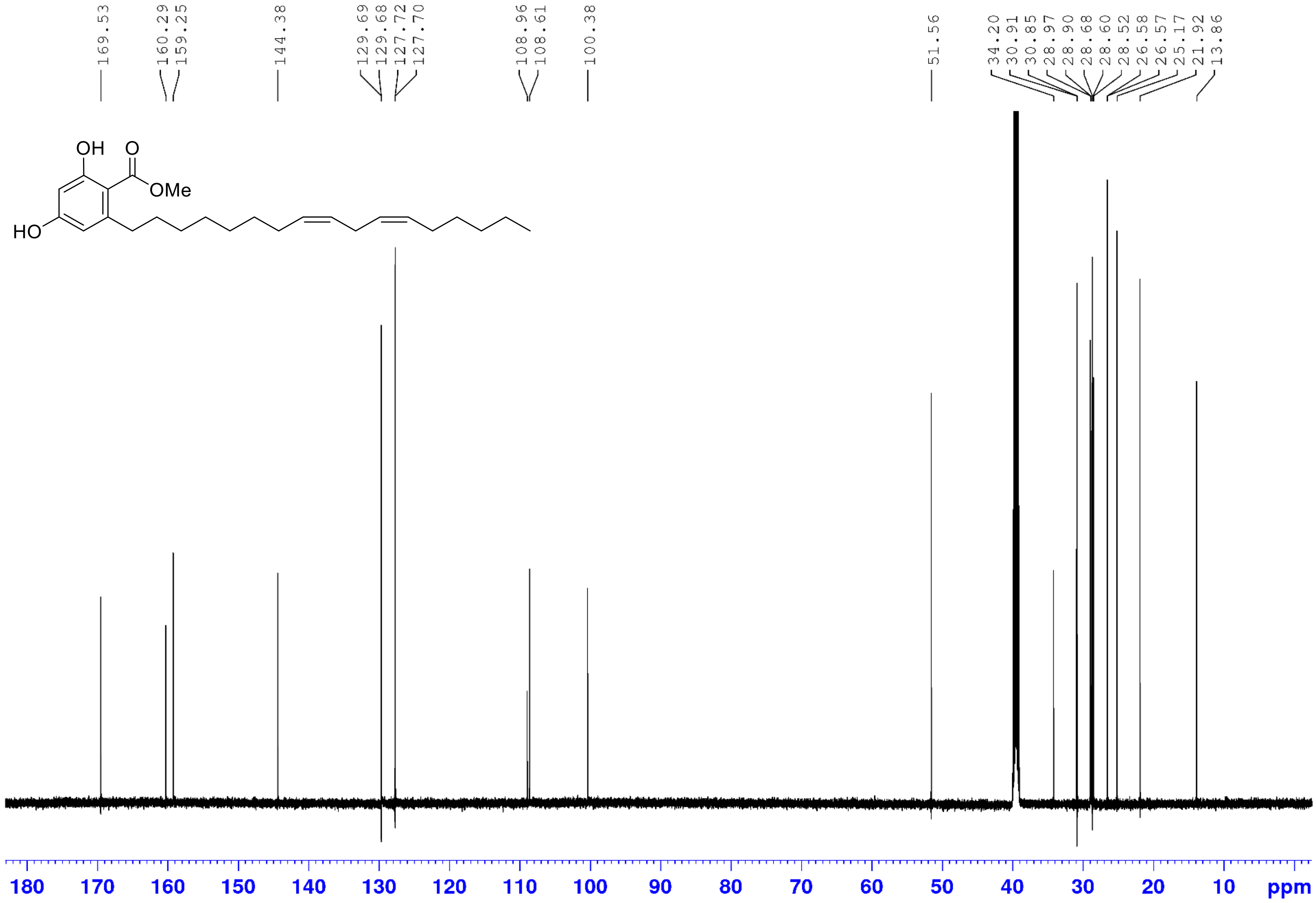

Figure S47. ^{13}C NMR spectrum (150 MHz) of geministatin E (**5**) in DMSO-d_6

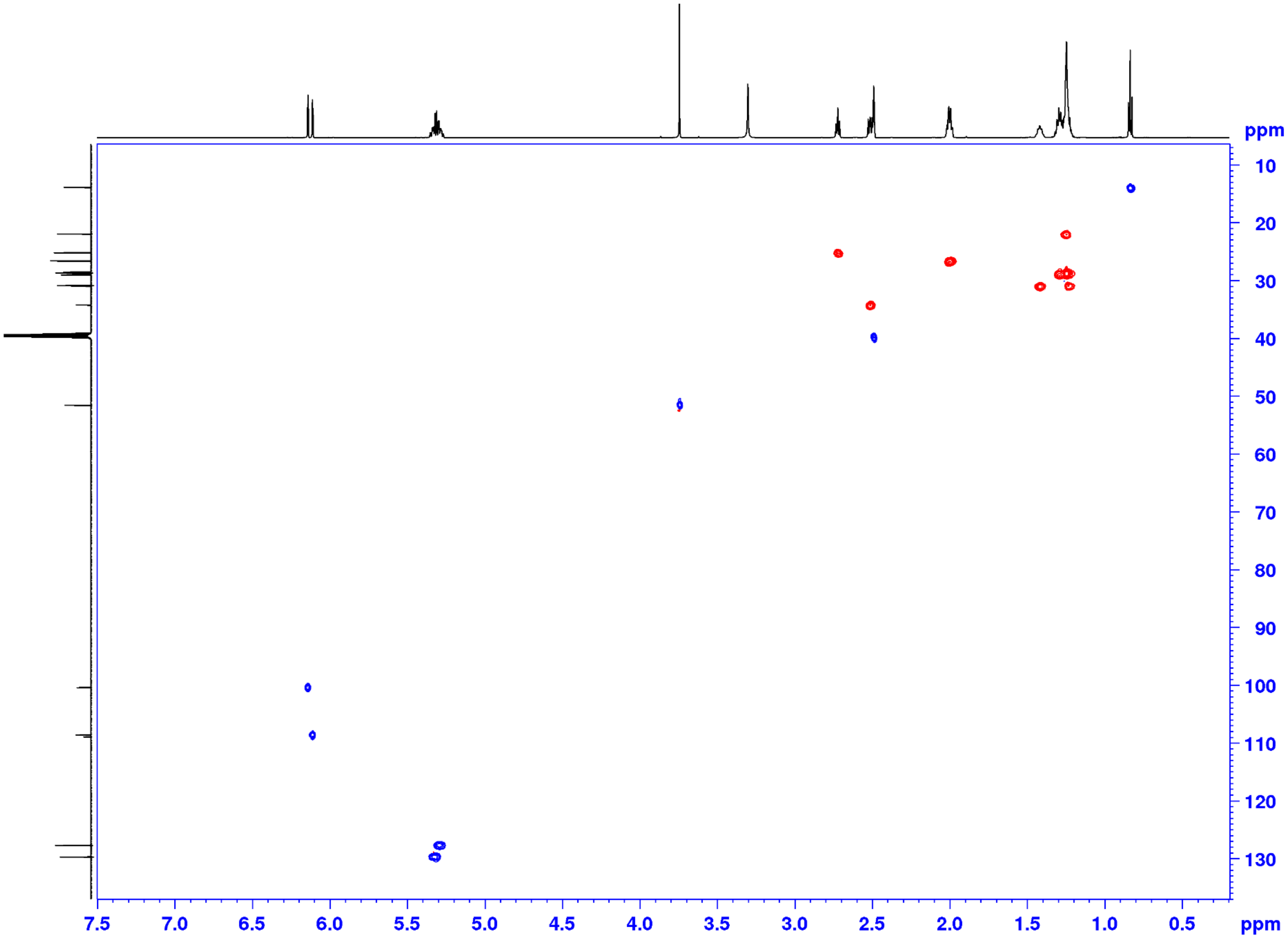

Figure S48. HSQC NMR spectrum (600 MHz) of geministatin E (**5**) in DMSO-d_6

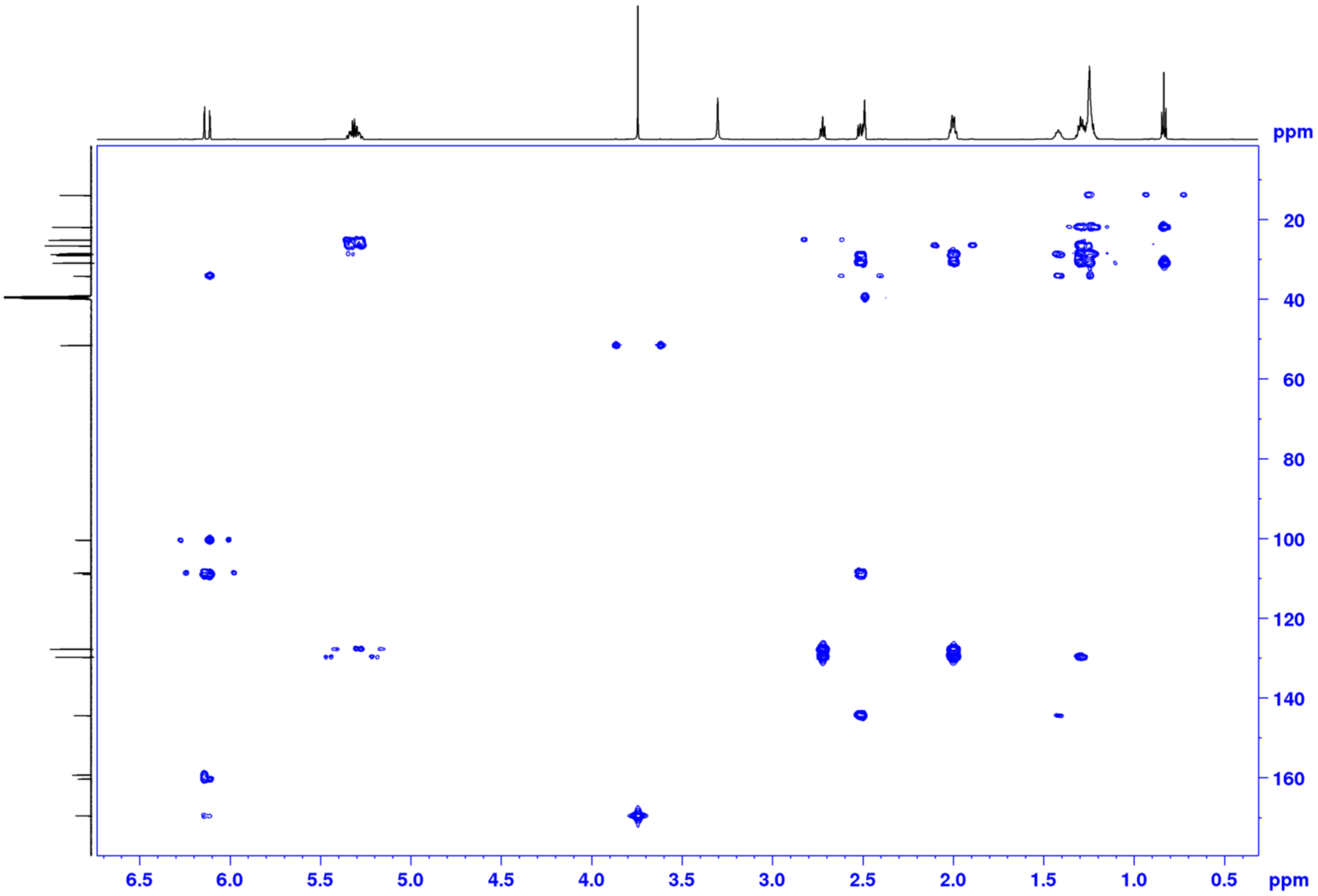

Figure S49. HMBC NMR spectrum (600 MHz) of geministatin E (**5**) in DMSO-d_6

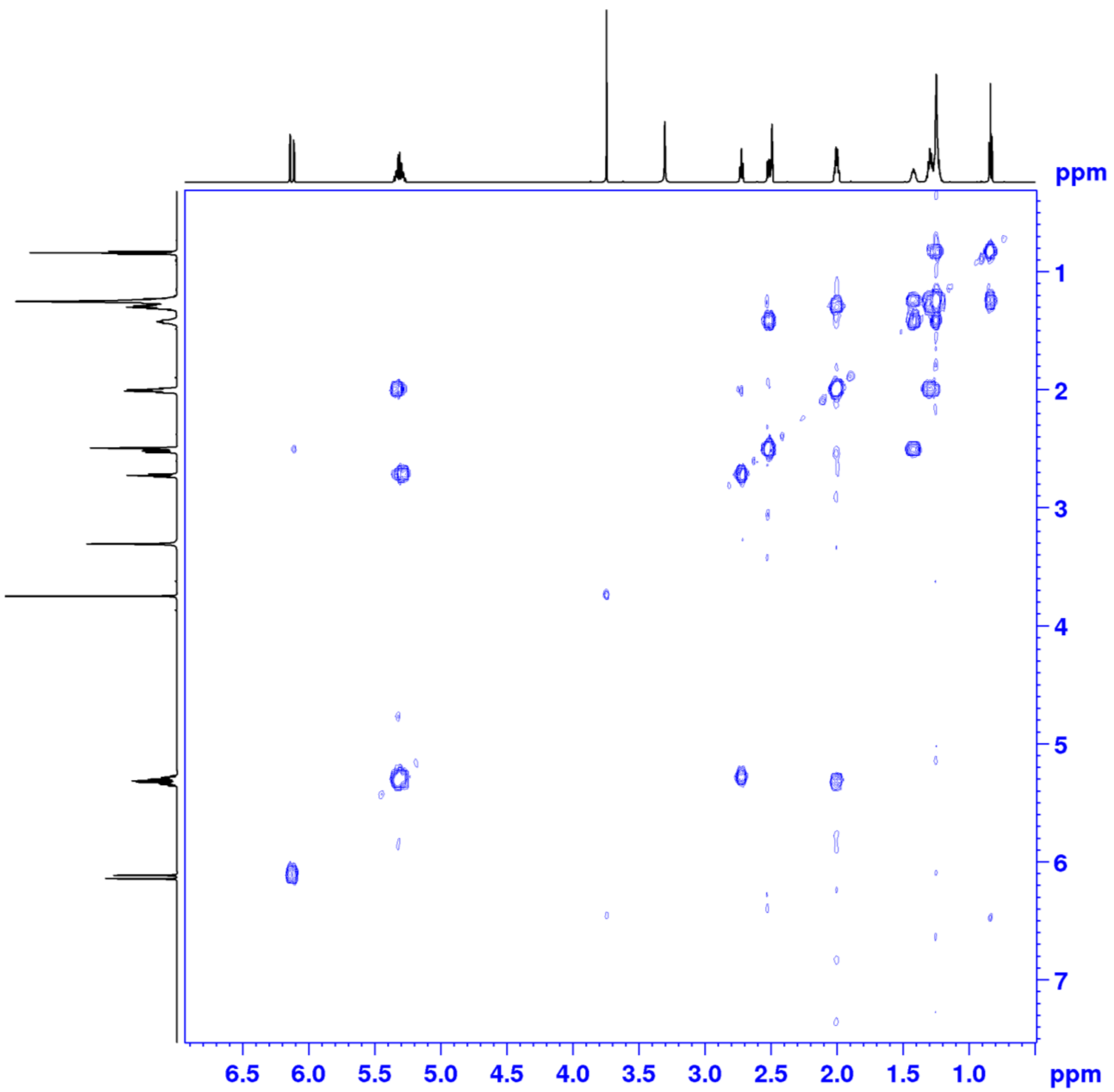

Figure S50. COSY NMR spectrum (600 MHz) of geministatin E (**5**) in DMSO-d_6

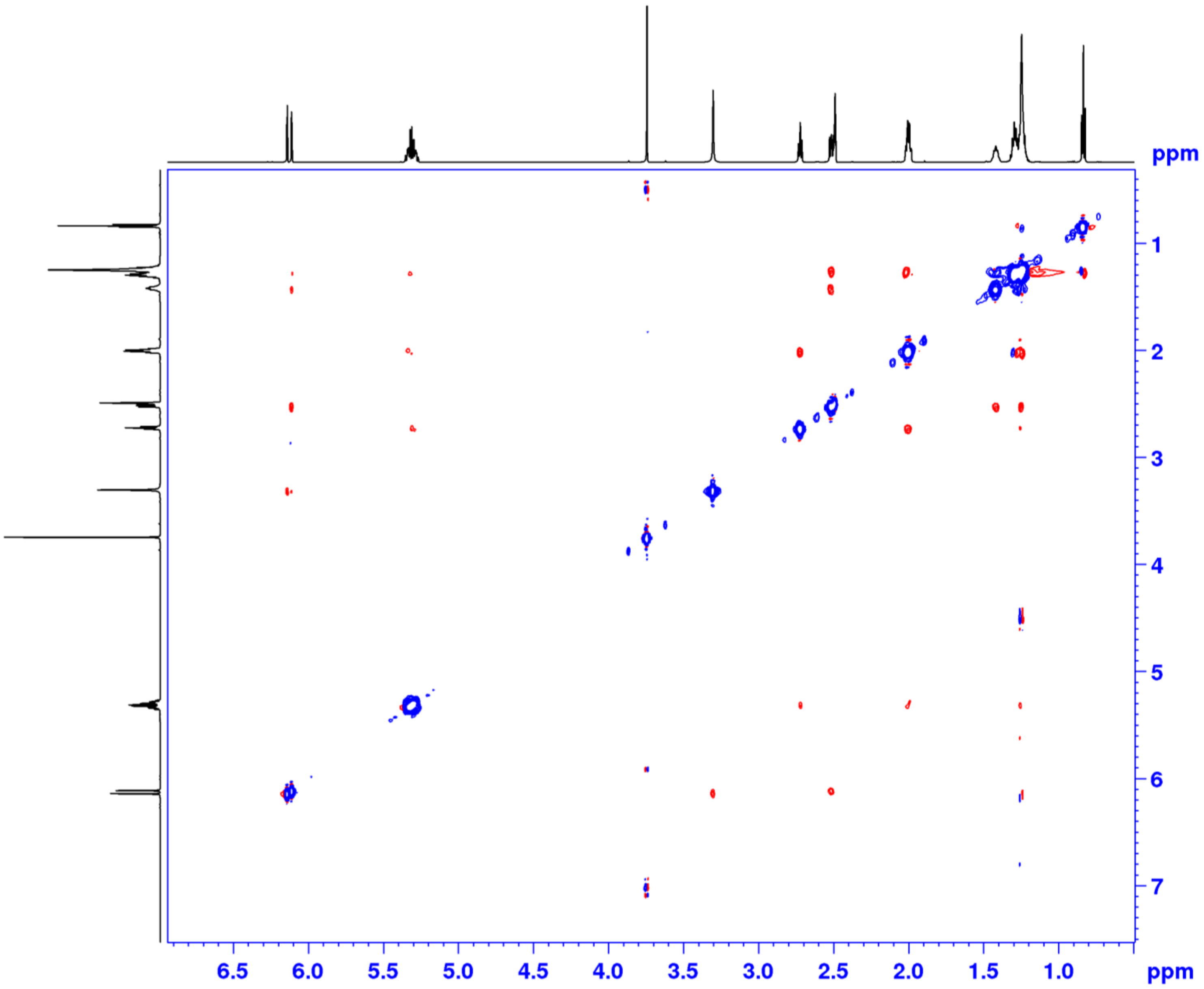

Figure S51. ROESY NMR spectrum (600 MHz) of geministatin E (**5**) in DMSO-d_6

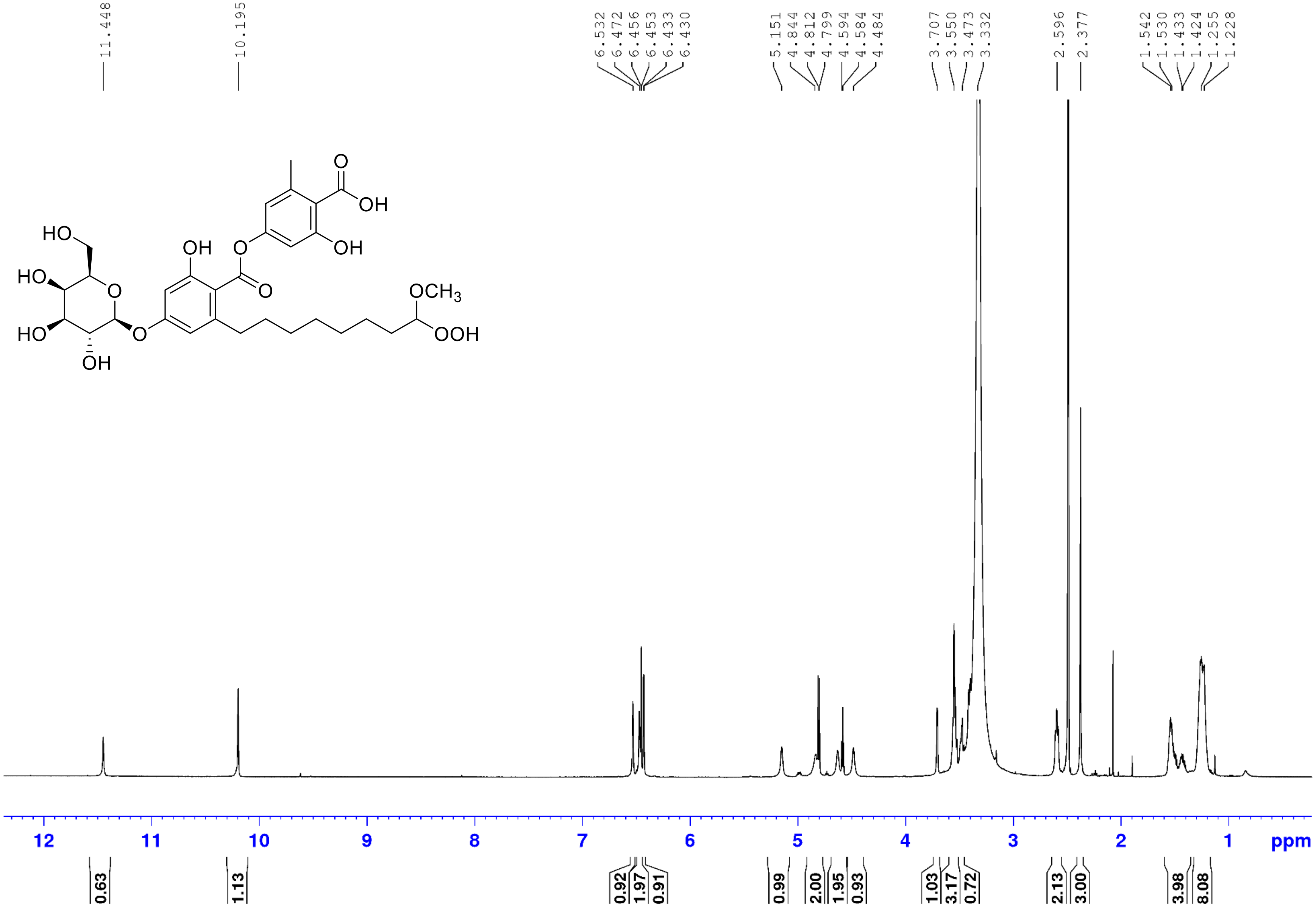

Figure S52. ^{1}H NMR spectrum (600 MHz) of geministatin A ozonolysis product (**7**) in DMSO-d_6

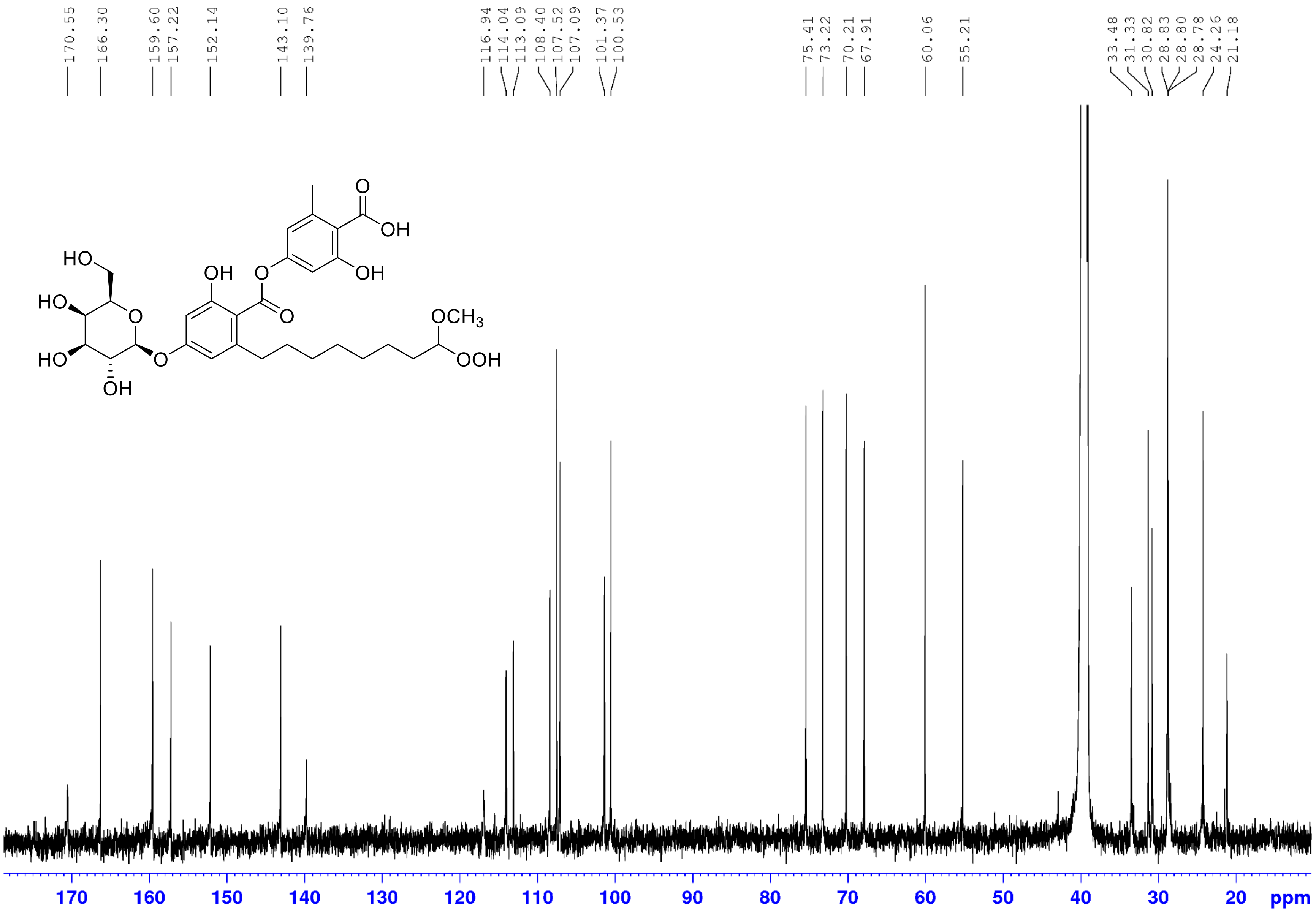

Figure S53. ^{13}C NMR spectrum (150 MHz) of geministatin A ozonolysis product (**7**) in DMSO-d_6

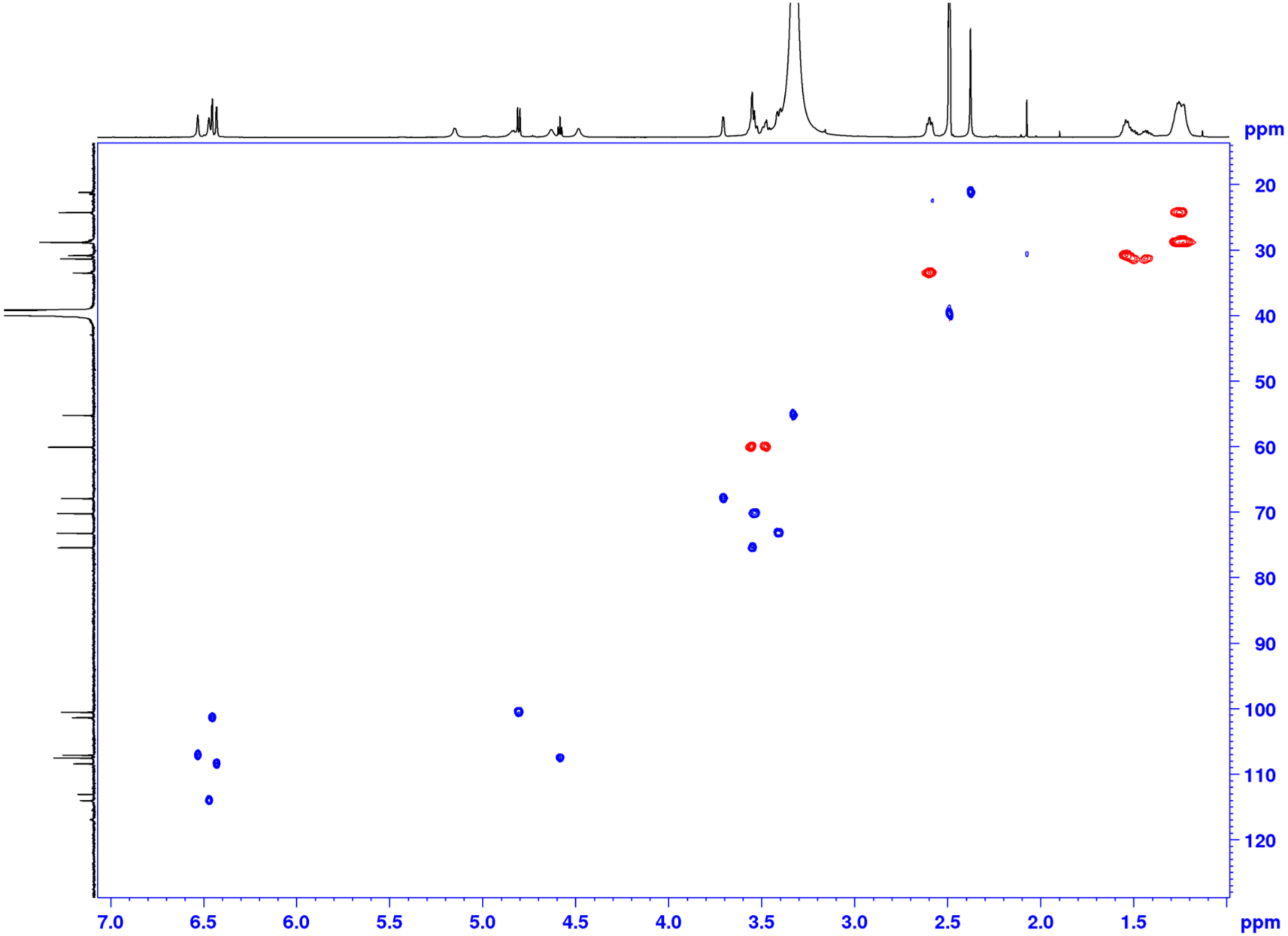

Figure S54. HSQC NMR spectrum (600 MHz) of geministatin A ozonolysis product (**7**) in DMSO-d_6

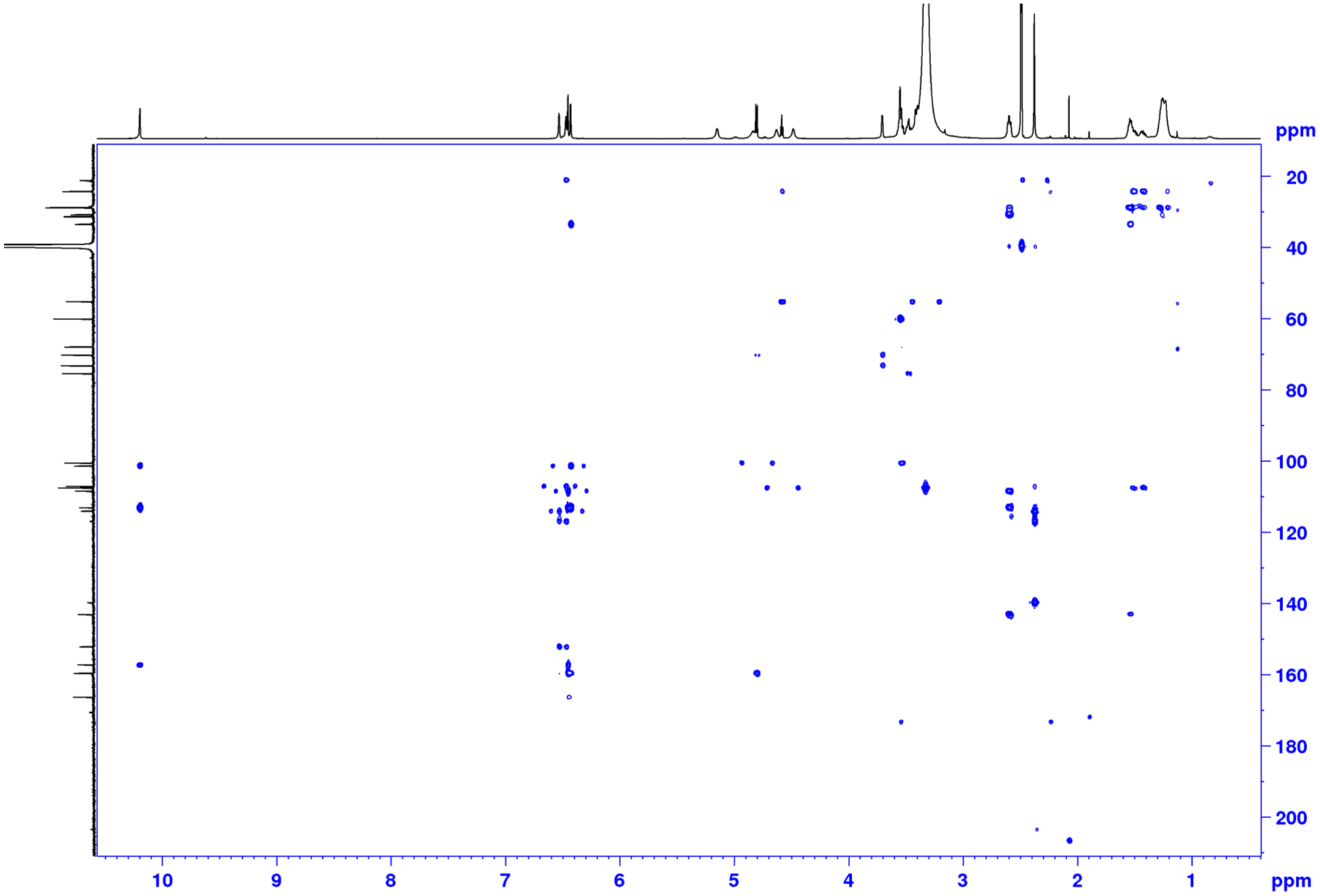

Figure S55. HMBC NMR spectrum (600 MHz) of geministatin A ozonolysis product (**7**) in DMSO-d_6

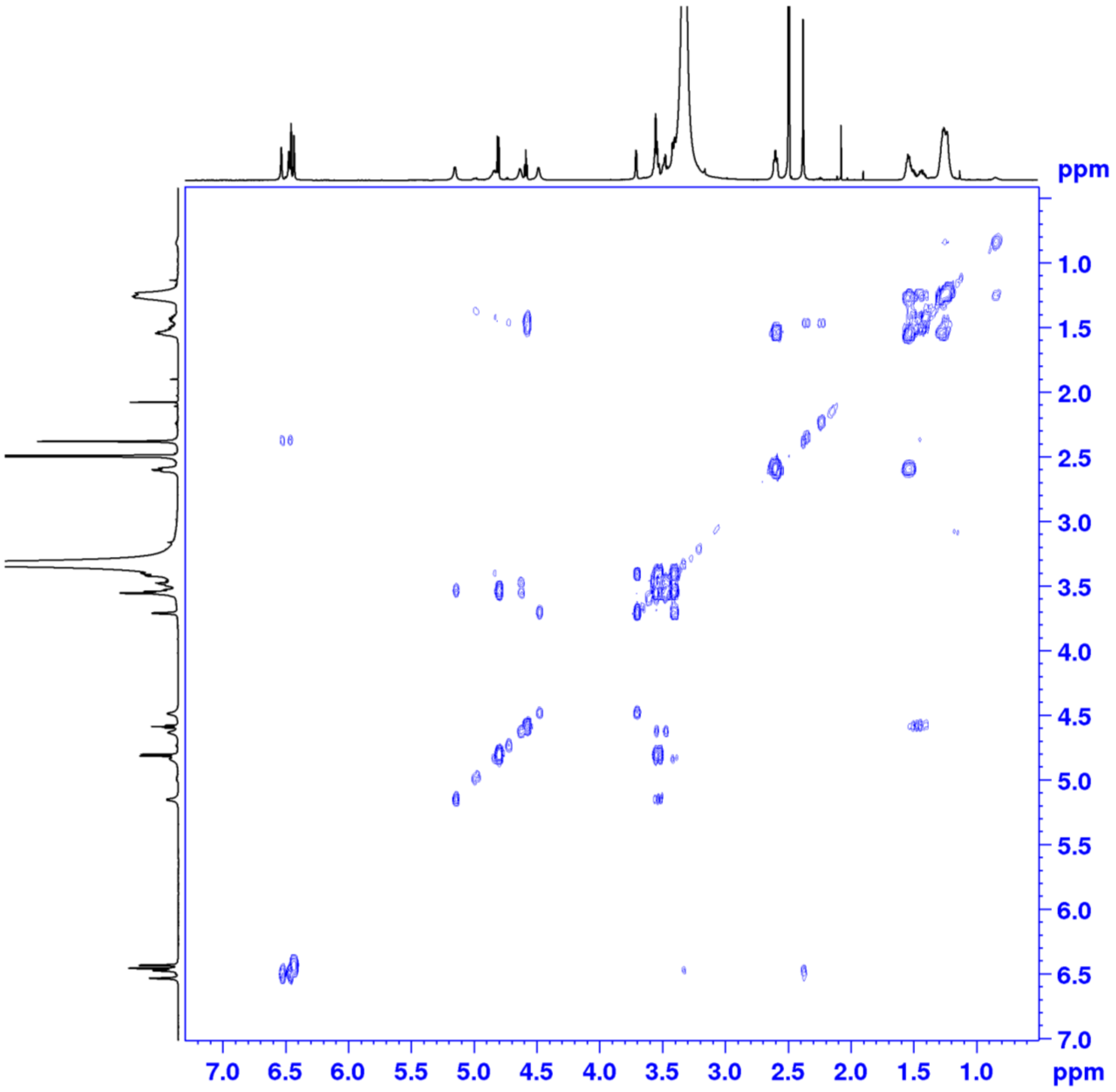

Figure S56. COSY NMR spectrum (600 MHz) of geministatin A ozonolysis product (**7**) in DMSO-d_6

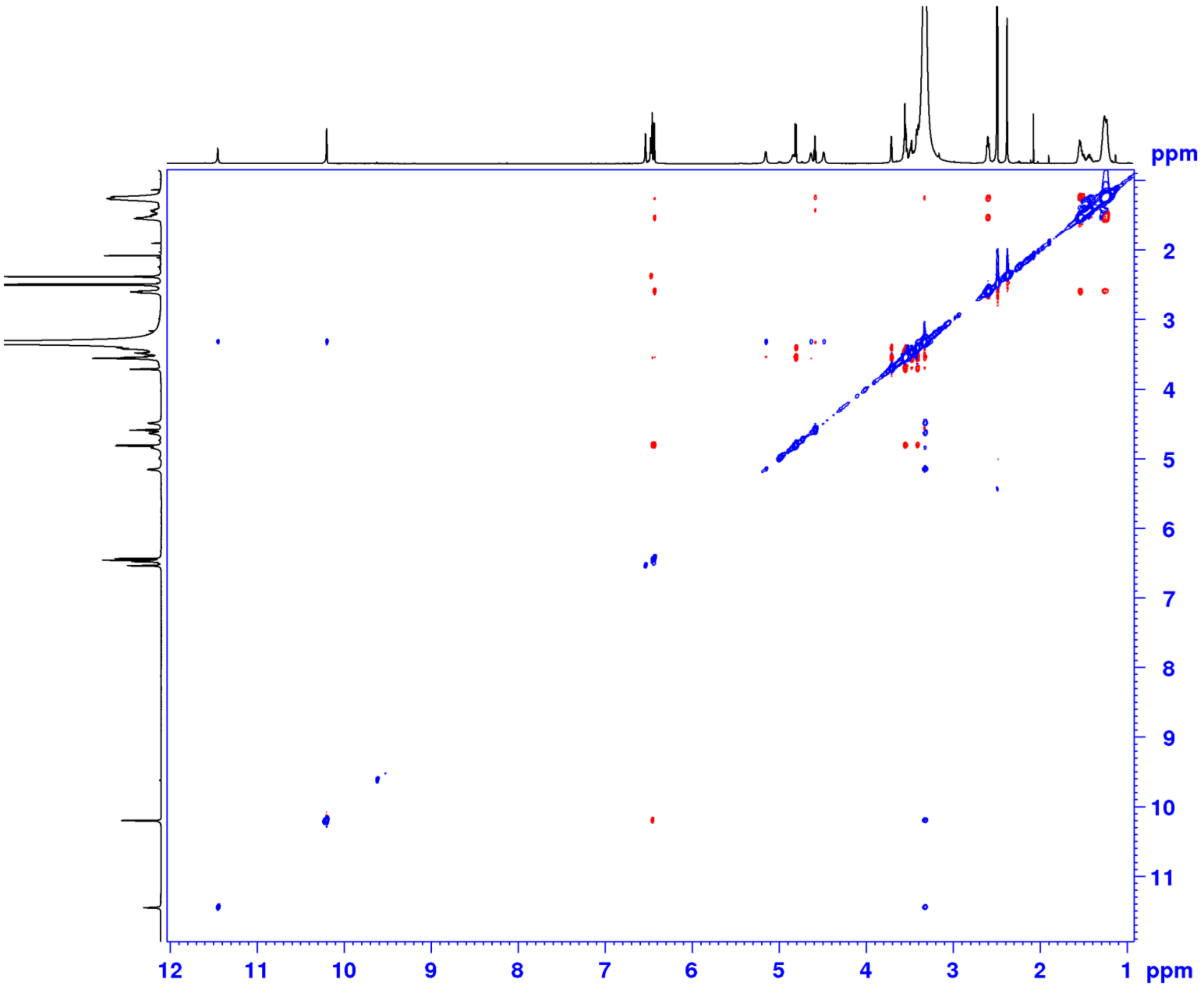

Figure S57. ROESY NMR spectrum (600 MHz) of geministatin A ozonolysis product (**7**) in DMSO-d_6
